# Macrophage-derived fibronectin suppresses antitumor immunity via tissue stiffening and immunosuppressive cell induction in cancer mouse models

**DOI:** 10.1038/s41467-026-73287-7

**Published:** 2026-05-22

**Authors:** Aitian Li, Ying Wang, Haiqing Bai, Xin Xie, Beibei Xu, Yunhan Wang, Shengjun Luo, Lei Zhang, Qitai Zhao, Shichao Duan, Huafang Zhao, Yacong Sun, Yu-Chieh Yuan, Xinxin Wang, Qinghe Qiao, Jiahui Cui, Chenyi Zhou, Huishang Wang, Lei Yang, Yang Yang, Longlong Si, Yi Zhang, Li Yang

**Affiliations:** 1 Biotherapy Center and Cancer Center, The First Affiliated Hospital of Zhengzhou University, Zhengzhou, Henan China; 2 Henan Academy of Innovations in Medical Science, Zhengzhou, Henan China; 3 Xellar Biosystems, Boston, MA USA; 4 CAS Key Laboratory of Quantitative Engineering Biology, Shenzhen Institute of Synthetic Biology, Shenzhen Institute of Advanced Technology, Chinese Academy of Sciences, Shenzhen, China; 5 Thoracic Surgery Department, The First Affiliated Hospital of Zhengzhou University, Zhengzhou, Henan China; 6 University of Chinese Academy of Sciences, Beijing, China; 7 School of Life Sciences, Zhengzhou University, Zhengzhou, Henan China

**Keywords:** Tumour immunology, Monocytes and macrophages, Extracellular matrix

## Abstract

Both tumor-associated macrophage (TAM) and tumor stiffness may support immunosuppression and limit immunotherapy response, particularly in non-small cell lung cancer (NSCLC). TAMs influence extracellular matrix (ECM) remodeling, but whether they also affect tumor stiffness, or are regulated by mechanical signals in turn, remains to be investigated. Here, we use single-cell transcriptomics of primary NSCLC samples to show that TAMs are associated with an immunosuppressive niche and are also a major source of the ECM component fibronectin (FN1). Mechanistically, macrophage-specific FN1 deficiency induces pro-inflammatory macrophages in a subcutaneous tumor mouse model, reduces ECM stiffness, increases lymphocyte infiltration into tumors, strengthens antitumor immunity, and enhances immune checkpoint blockade efficacy. Within TAMs, FN1-mediated cytoskeleton assembly and autophagy induction impair macrophage glycolysis by inhibiting the RAC1-mTOR axis, thereby limiting the antitumor activity of macrophages. Collectively, these findings highlight macrophage-derived FN1 as a mechanical cue for aggravating immunosuppression and as an intervention target to supplement immunotherapy in NSCLC.

## Abstract (web summary)

Tumor-associated macrophages and tumor stiffness may both support an immunosuppressive milieu in non-small cell lung cancer, but the underlying crosstalk remains unclear. Here, the authors identify macrophage-derived fibronectin as an immunosuppressive agent, both promoting extracellular matrix stiffness and inducing immunosuppressive immune cells.

## Introduction

Immunotherapy has become a common antitumor therapy administered following surgical interventions, radiotherapy, chemotherapy, or other traditional treatment methods^1^. Nevertheless, compared with the progress in hematological malignancies, the application of immunotherapy for treating solid tumors remains limited^2,3^. The tumor microenvironment (TME) contributes to the insufficient therapeutic effects of immunotherapy^4^. The TME supports tumor occurrence and growth and comprises a complex network of immune and non-immune cells, the extracellular matrix (ECM), and diverse signaling molecules^5^. The negative effects of the TME arise from the dense and fibrotic ECM, which restricts T-cell infiltration, and from immunosuppressive cells,  which inhibit the cytotoxic activity of T cells^6–8^.

Solid tumors create immune-exclusion niches in which stromal cells generate aberrant matrix deposits that form a physical barrier, limiting the recruitment of T cells into the tumor milieu^6^. Various physical characteristics of cancer are used as diagnostic and prognostic markers of tumor growth, including matrix deposition and tissue remodeling-mediated stiffening^9^. Increased stiffness activates signaling pathways that promote cancer cell proliferation, invasion, and metastasis^10^. A stiff ECM directly leads to CD8^+^ T-cell exhaustion^11^. While matrix stiffening was originally thought to be induced primarily by fibroblasts, macrophages also contribute to this effect by regulating the ECM^12^. Within the TME, localized tumor-associated fibroblast (CAF) and tumor-associated macrophage (TAM) stimulate the formation of immunologically rejected desmoplastic structures and limit T-cell infiltration^13^. TAMs can also upregulate the production of collagen by secreting TGF-β, activate stromal cells, and promote the formation of a fibrotic environment^14^. Additionally, TAMs can differentiate into CAFs through macrophage–myofibroblast transition^15^. Despite the growing body of research characterizing the mechanical properties associated with ECM remodeling, the role of macrophages in driving tumor stiffness and regulating mechanical signals remains largely unexplored.

TAMs are key drivers of immunosuppression in non-small cell lung cancer (NSCLC), with versatile roles in regulating tumor progression^16,17^. Monocyte-derived TAM (Mo-TAM) is a heterogeneous population of highly plastic cells among the prevailing immune infiltrates in NSCLC^18,19^. Unlike the antitumor macrophages that exhibit lymphocyte trafficking and pro-inflammatory responses, the pro-tumor macrophages generally display a restorative and tumor-supportive phenotype with metabolic reprogramming^16,20,21^. Moreover, although microenvironmental cues influence TAMs, the pathways by which mechanical properties regulate TAM polarization remain largely unknown.

Fibronectin (FN1) is among the most important adhesion molecules in the ECM, with vital roles in regulating cell adhesion, migration, and proliferation^22,23^. FN1 exists as a dimer or multimer on the cell surface or in the matrix and can bind ECM proteins, such as collagen, promoting their assembly and matrix formation, creating a fibrotic environment^22,24,25^. Macrophage-produced FN1 can attract fibroblasts and is considered a marker of TAMs in several cancer types^26,27^. However, whether macrophage-secreted FN1 contributes to tissue stiffening and how it skews TAM phenotype polarization remains elusive.

This study demonstrates that macrophage-derived FN1 promotes tumor matrix stiffening and establishes an immunosuppressive tumor microenvironment. Mechanistically, FN1 drives cytoskeletal assembly to sequester RAC1, thereby inhibiting mTOR signaling and inducing autophagy—processes that suppress glycolysis and sustain immunosuppressive macrophage function. In a subcutaneous tumor mouse model, macrophage-specific FN1 deletion reverses tumor stiffness, abrogates immunosuppressive macrophage function, and alleviates the overall immunosuppressive milieu, ultimately potentiating the efficacy of immune checkpoint blockade (ICB). Collectively, these findings identify macrophage-derived FN1 as a mechanobiological determinant of tumor immunosuppression, suggesting that FN1 inhibition may serve as a complementary immunotherapeutic strategy to enhance anti-tumor immunity in NSCLC.

## Results

### FN1+ macrophages are enriched in NSCLC and associated with an immunosuppressive niche

**Fig. 1:**
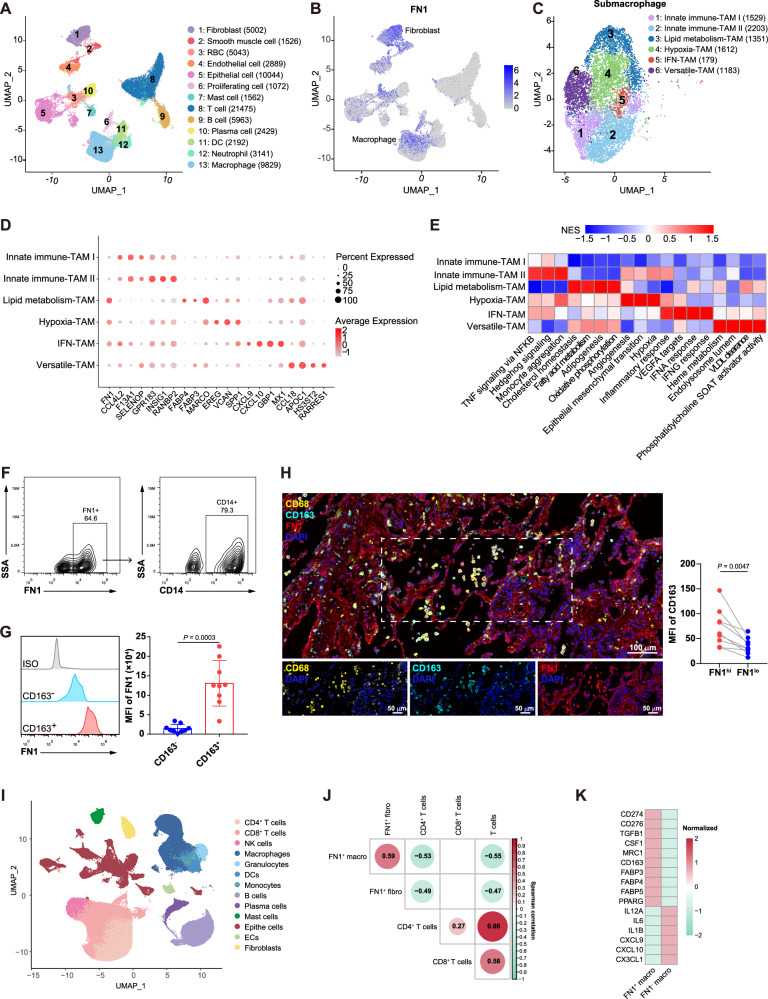
FN1 is enriched in tumor-associated macrophages of NSCLC. **A** Uniform Manifold Approximation and Projection (UMAP) plot of major cell lineages from human NSCLC tumor tissues, *n* = 10 biologically independent samples. (**B**) UMAP plot colored by FN1 (grey to blue). **C** UMAP plot of macrophage subsets. **D** Macrophage-specific markers. **E** Gene set variation analysis (GSVA) across macrophage subclusters. **F** Representative FACS plot of FN1 levels in tumor-infiltrating CD14^+^ macrophages measured by flow cytometry. **G** FN1 expression in CD163^+^ or CD163^-^ macrophages was measured by flow cytometry. Data are displayed as the mean ± SD, *n* = 9 biologically independent samples, two-tailed paired *t*-test. **H** Representative mIHC staining image of CD68, CD163, and FN1 from 9 NSCLC tumor tissues. Scale bar, 100 μm and 50 μm. Quantification of CD163 in CD68^+^ FN1-hi vs CD68^+^ FN1-lo TAMs. *n* = 9 biologically independent samples, two-tailed paired *t*-test. **I** UMAP plots of cells obtained from public scRNA-seq data, GSE131907. **J** Spearman correlation of the proportions of FN1^+^ macrophages or FN1^+^ fibroblasts and immune cells (CD4^+^ and CD8^+^ T cells) from public scRNA-seq data, GSE131907. **K** Heatmap of immunosuppressive, lipid metabolism-related, and pro-inflammatory genes in FN1^+^ and FN1^-^ macrophages from public scRNA-seq data, GSE131907. NSCLC non-small cell lung cancer, FACS fluorescence-activated cell sorting, mIHC multiplex Immunohistochemistry, scRNA-seq single-cell RNA sequencing, NES normalized enrichment score, MFI mean fluorescence intensity.

Monocytes express minimal FN1; however, once polarized, FN1 production increases, especially in alternatively activated macrophages^28^. To investigate the expression of FN1 in the TME of NSCLC, single-cell RNA sequencing (scRNA-seq) derived from ten cases of primary NSCLC was evaluated. Through unbiased clustering, 67,124 single cells were identified and organized into 12 main cell types (Fig. 1A and Supplementary Fig. 1A). In addition to the known expression of FN1 by fibroblasts, macrophages specifically and highly expressed FN1 (Fig. 1B).

An analysis of public scRNA-seq repositories for NSCLC was performed to confirm the relatively conserved expression of FN1 in TAMs (Supplementary Fig. 1B). The TAMs were divided into six cellular states, underscoring the cellular diversity and distinct molecular features (Fig. 1C–E). Clusters 1 and 2, comprising innate immune-associated TAMs, were highly enriched in the hedgehog, tumor necrosis factor (TNF), and nuclear factor (NK)- κB signaling pathways and expressed genes with characteristic anti-viral activity (*CCL4L2, F13A1, SELENOP*, and *GPR183*). Cluster 3 primarily included pathways and genes related to lipid metabolism (*FABP3, FABP4*, and *MARCO*). Cluster 4 was defined as hypoxia-associated TAMs due to the upregulation of genes associated with hypoxia, epithelial–mesenchymal transition (EMT), and angiogenic features (*EREG, VCAN*, and *SPP1*). Cluster 5 expressed genes encoding interferon (IFN)-response signatures (*CXCL9, CXCL10, GBP1*, and *MX1*). Cluster 6 comprised versatile TAMs expressing genes associated with the synthesis and degradation of myriad substances (*CCL18, APOC1, HS3ST2*, and *RARRES1*). FN1 expression was most enriched in lipid metabolism-associated TAMs (Cluster 3; Fig. 1D), exhibiting activation of oxidative phosphorylation and lipid metabolism (cholesterol and fatty acid metabolism) (Fig. 1E). Multiple studies have reported that macrophages with these metabolic characteristics possess enhanced immunosuppressive functions^20,29^.

To further evaluate the expression pattern of FN1, flow cytometry and multiplex immunohistochemistry (mIHC) were employed to verify that FN1 was highly expressed in macrophages, especially in the immunosuppressive phenotype (Fig. 1F–H). The results showed an abundance of FN1 in the tumor milieu (Fig. 1H). To investigate whether FN1 expression was consistent between human- and mouse-derived macrophages, public bulk RNA-seq datasets of THP-1-induced and bone marrow-derived macrophage (BMDM) were evaluated. The transcriptomic profiles appeared largely conserved across different species (Supplementary Fig. 1C, D). Human peripheral blood monocyte-derived macrophage (MDM) also exhibited prominent FN1 expression in an immunosuppressive phenotype (Supplementary Fig. 1E–G).

Taking into consideration that FN1 is related to a dense fibrotic environment, we further evaluated whether TAM-derived FN1 was associated with restricted T-cell infiltration. Two large-scale NSCLC scRNA-seq datasets were used to analyze the correlation between macrophage- or fibroblast-derived FN1 and the abundance of CD4^+^ and CD8^+^ T-cell infiltration. Consequently, a close negative correlation was elucidated between FN1^+^ macrophages and T-cell infiltration, compared to FN1^+^ fibroblasts (Fig. 1I, J and Supplementary Fig. 1H, I). This indicated that macrophage-derived FN1 exerted a suppressive effect on T-cell infiltration. Furthermore, these FN1^+^ macrophages also exhibited an immunosuppressive phenotype; however, FN1^-^ macrophages expressed more pro-inflammatory genes (Fig. 1K and Supplementary Fig. 1J). All these data provide insight into the enrichment of FN1^+^ macrophages in NSCLC, along with the immunosuppressive signature presented by them, which is negatively correlated with T-cell infiltration.

### Macrophage-specific FN1 deficiency remodels the TME

To explore the role of macrophage-derived FN1 in facilitating tumor progression, myeloid cell-specific FN1 knockout (FN1^ΔLyz2^) mice were generated by crossing FN1^fl/fl^ mice with Lyz2-Cre mice (Supplementary Fig. 2A). Excluding the FN1 highly expressed by macrophages, FN1 expression was rarely observed in other myeloid subpopulations, including dendritic cells (DC) or neutrophils (Fig. 1B and Supplementary Fig. 1B). Therefore, FN1^ΔLyz2^ mice achieved a specific knockout of FN1 in macrophages.

**Fig. 2:**
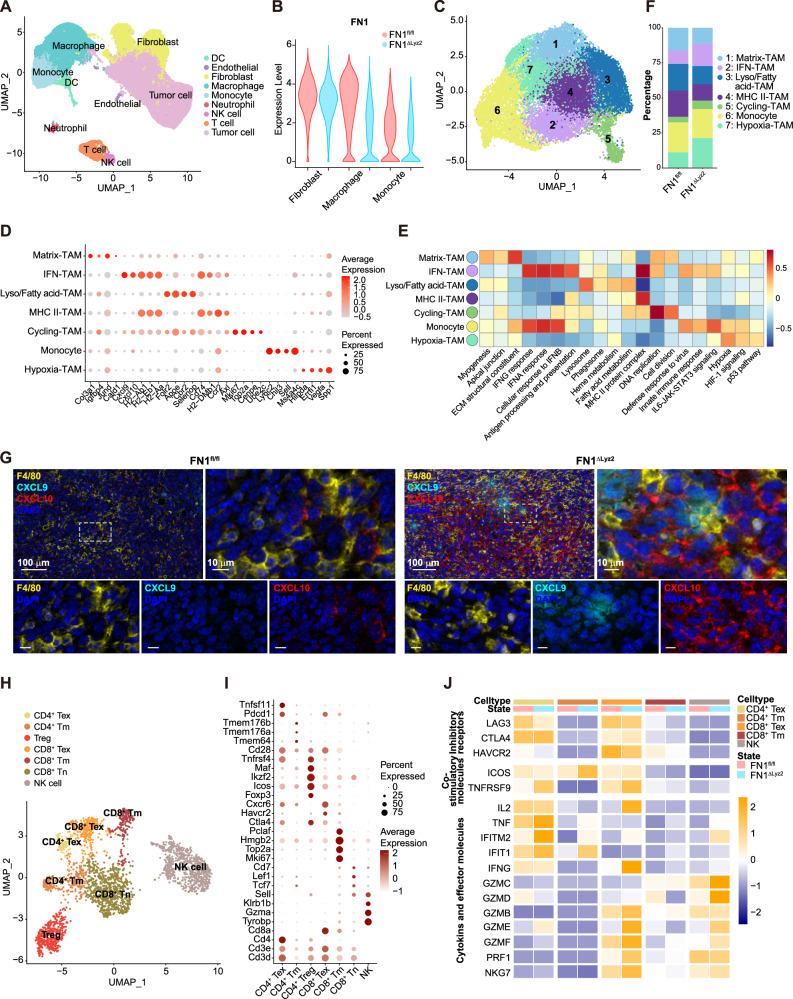
FN1 deficiency redirects macrophages toward the pro-inflammatory phenotype and reprograms the TME. **A** UMAP plot of main cell lineages in LLC tumors from FN1^fl/fl^ and FN1^ΔLyz2^ mice, *n* = 3 biologically independent samples. **B** Expression of FN1 in fibroblasts, macrophages, and monocytes. **C** UMAP plot of macrophage subsets. **D** Macrophage subcluster-specific markers. **E** GSVA across macrophage subpopulations. **F** Profiles of macrophage subpopulations. **G** Representative mIHC staining image of F4/80, CXCL9, and CXCL10 from tumor tissues of FN1^fl/fl^ and FN1^ΔLyz2^ mice. Scale bar, 100 μm and 10 μm. Experiment was repeated three times independently with similar results. **H** UMAP plot of T and NK cell subsets. **I** Expression levels of marker genes in T and NK cells. **J** Gene expression in T and NK cells of FN1^fl/fl^ and FN1^ΔLyz2^ mice, respectively. GSVA gene set variation analysis, mIHC multiplex immunohistochemistry, NK natural killer, ECM extracellular matrix.

The efficiency of the FN1 knockout in BMDMs (Supplementary Fig. 2B) was further confirmed using scRNA-seq of Lewis lung carcinoma (LLC) tissues from FN1^ΔLyz2^ mice and their littermates. In total, 53,566 cells from six samples (FN1^fl/fl^ = 3 and FN1^ΔLyz2^ = 3) were categorized into nine major clusters: monocytes, macrophages, DCs, neutrophils, T cells, natural killer (NK) cells, fibroblasts, tumor cells, and endothelial cells (Fig. 2A and Supplementary Fig. 2C). Consistent with the human scRNA-seq observations, FN1 was expressed primarily in macrophages and fibroblasts (Fig. 2B). Macrophages and monocytes from FN1^ΔLyz2^ mice showed lower FN1 levels than those from FN1^fl/fl^ mice; however, fibroblasts were unaffected, confirming the specific loss of FN1 in macrophages (Fig. 2B).

Further clustering of monocytes and macrophages created seven subpopulations with distinctive gene signatures comprising one monocyte group and six macrophage groups (Fig. 2C). The Matrix–TAM cluster exhibited prominent extracellular matrix structure constituents characterized by the upregulation of collagen and cell contraction genes (*Col3a1, Igfbp4*, and *Cald1*); the IFN–TAM cluster featured gene modules involved in IFN responses (*Cxcl9* and *Cxcl10*) and antigen processing and presentation (MHC class II molecules); the Lyso/Fatty acid–TAM cluster exhibited upregulation of several metabolic-related genes (*Folr2, Apoe*, and *Cbr2*) and was enriched in lysosome and fatty acid metabolism; the MHC II–TAM cluster was characterized by enriched MHC class II protein complex (*H2-AB1, H2-Eb1, H2-Aa, H2-Dmb1*, and *Cd74*) and monocyte recruitment (*Ccr2*), and lacked the IFN response gene; the Cycling–TAM cluster was enriched in cell cycle genes (*Mki67, Top2a, Cenpe*, and *Ube2c*) and featured the DNA replication program; the Hypoxia–TAM cluster exhibited upregulated genes involved in the hypoxia reaction (*Hilpda, Errfi1*, and *Spp1*); and the Monocyte cluster was identified by *Ly6c2, Chil3*, and *Sell*, and was related to the IFN response (Fig. 2D, E). Importantly, FN1^ΔLyz2^ mice showed a reduced proportion of Lyso/Fatty acid–TAM (Fig. 2F), suggesting a positive correlation between FN1 and macrophage lipid metabolism/lysosomal function. Macrophages with enriched lipid metabolism demonstrate enhanced immunosuppressive capabilities^30,31^. This provides mechanistic insight into how FN1-mediated immunosuppressive functions in TAMs through the lysosomal pathway. Besides, a smaller proportion of Matrix–TAMs and a higher frequency of IFN–TAMs were detected in the FN1^ΔLyz2^ mice than in their littermate controls (Fig. 2F). This suggested a softer matrix, more pro-inflammatory macrophages with high CXCL9 and CXCL10 levels (Fig. 2D) T cell recruitment-associated chemokines^32^, and enhanced tumor-infiltrating T cells in the FN1-deficient TME. The mIHC results showed higher CXCL9 and CXCL10 levels in the TME of FN1^ΔLyz2^ mice (Fig. 2G). These data highlight the potential role of TAM-derived FN1 in orchestrating macrophage reprogramming, ECM remodeling, and T-cell infiltration.

To further evaluate the effect of FN1 on macrophage reprogramming, three complementary experimental systems were employed: (1) THP-1-induced macrophages with FN1 knockdown via shRNA (Supplementary Fig. 2D, E), (2) MDMs treated with an RGD peptide—fibronectin blocker (FB)^33^, which competitively inhibits integrin-ECM interactions, including but not limited to fibronectin-integrin binding; and (3) BMDMs from FN1^fl/fl^ and FN1^ΔLyz2^ mice, to specifically dissect FN1-dependent mechanisms. Following FN1 blockade, levels of anti-inflammatory molecules (CD163, CD206, and TGF-β) decreased, while pro-inflammatory molecules (CD86, IL-6, IL-1β, TNF, CXCL9 and CXCL10) increased (Supplementary Fig. 2F–N). This confirmed that disrupting FN1 promotes macrophage reprogramming into a pro-inflammatory phenotype.

A stiff ECM reportedly promotes T-cell exhaustion^11^. Thus, the effect of FN1^ΔLyz2^ tumors on the function of tumor-killer cells was evaluated. The T and NK cells were assigned various clusters: three CD8^+^ T-cell clusters (exhausted, memory, and naïve CD8^+^ T-cells), three CD4^+^ T-cell clusters (exhausted, memory, and regulatory CD4^+^ T cells), and one NK cell cluster (Fig. 2H, I). Higher levels of TNF- and IFN-stimulated genes (*Ifitm2* and *Ifit1*) were expressed in CD4^+^ T cells, while IFN-γ (*Ifng*), perforin (*Prf1*), and granzyme (*Gzm*) were upregulated in the CD8^+^ T and NK cells of FN1^ΔLyz2^ mice compared with their littermate controls (Fig. 2J). To further evaluate the impact of FN1 on T cell function, murine CD8^+^ T cells were co-cultured with FN1^fl/fl^ and FN1^ΔLyz2^ BMDMs, respectively. Consistent with the results of scRNA-seq, CD8^+^ T cells in the FN1^ΔLyz2^ group exhibited higher levels of IFN-γ and perforin (Supplementary Fig. 3A). These data suggest that FN1 contributes to the polarization of pro-tumoral macrophages and TME remodeling, exacerbating an immunosuppressive tumor niche.

### FN1 reinforces ECM stiffness to impede T-cell infiltration

**Fig. 3:**
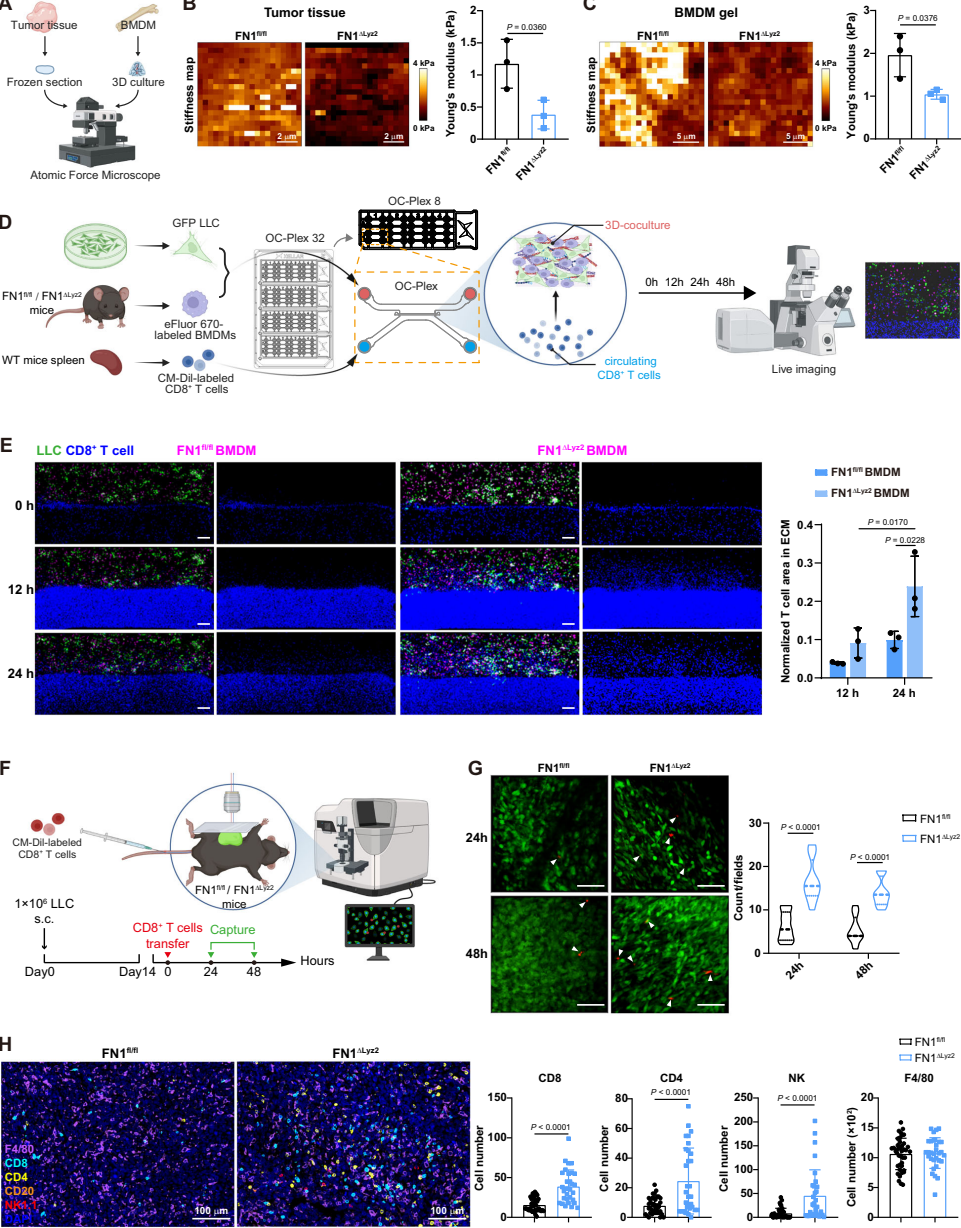
Macrophage-derived FN1 enforces tissue stiffness and excludes T cells. **A** Schematic diagram of elastic modulus measurement of tumor tissues and ECM. Created in BioRender. Li, Y. (2026) https://BioRender.com/vk6cajb. Representative AFM images and quantification of the stiffness in tumor tissues (**B) and** ECM from BMDMs (**C**) of FN1^fl/fl^ and FN1^ΔLyz2^ mice. *n* = 3 biologically independent samples. Data are displayed as the mean ± SD. two-tailed unpaired *t*-test. **D** Scheme of 3D tumor-immune cell co-culture system model. Created in BioRender. Li, Y. (2026) https://BioRender.com/vk6cajb. **E** Representative confocal images of Dil-labeled CD8^+^ T-cell infiltration in LLC–BMDM co-culture hydrogel matrix in the presence of CXCL9 (50 ng/mL) at 0 h, 12 h, and 24 h. Scale bar, 100 μm. Quantification of Dil-labeled CD8^+^ T-cell migration to the matrix in confocal images. *n* = 3 biologically independent samples. Data are displayed as the mean ± SD. two-way ANOVA. **F** Flow chart of Dil-labeled CD8^+^ T-cell infiltration observed by IVM in vivo. Created in BioRender. Li, Y. (2026) https://BioRender.com/vk6cajb. **G** Representative in vivo images of Dil-labeled CD8^+^ T-cell infiltration in GFP–LLC tumors at 24 h and 48 h after cell transfer. CD8^+^ T cells were indicated with white arrows. Scale bar, 100 μm. Quantification of Dil-labeled CD8^+^ T cell migration to tumors in live images. *n* = 3 biologically independent samples. two-way ANOVA. **H** Representative mIHC staining image of F4/80, CD4, CD8, NK1.1, CD20 and DAPI of tumor tissues from FN1^fl/fl^ and FN1^ΔLyz2^ mice. Scale bar, 100μm. Quantification of the single positive cell number of FN1^fl/fl^ and FN1^ΔLyz2^ tumor. *n* = 3 biologically independent samples. Each dot represents one ROI. Data are displayed as the mean ± SD. two-tailed unpaired *t*-test. AFM atomic force microscope, ECM extracellular matrix, BMDM bone marrow-derived macrophage, LLC Lewis lung carcinoma, IVM intravital microscopy, ROI region of interest.

To explore the role of FN1 in regulating ECM rigidity, the stiffness of tumor tissues in FN1^fl/fl^ and FN1^ΔLyz2^ mice was measured (Fig. 3A). The solid tumor tissues of FN1^ΔLyz2^ mice exhibited lower stiffness than the FN1^fl/fl^ mice (Fig. 3B and Supplementary Fig. 4A). Moreover, the atomic force microscopy (AFM) results revealed that FN1-deficient BMDMs possessed a weaker ability to stiffen the mechanical properties of collagen compared to control BMDMs (Fig. 3C and Supplementary Fig. 4B).

As FN1-expressing macrophages demonstrated the ability to remodel the ECM (Fig. 3A–C), and were negatively correlated with T-cell infiltration (Fig. 1J and Supplementary Fig. 1I), the disruption of FN1 in macrophages was hypothesized to accelerate CD8^+^ T-cell infiltration in the TME. To test this hypothesis, a triple-culture system was developed using a scalable OC-Plex organ chip platform to mimic T-cell infiltration in solid tumors. Each OC-Plex 32 plate contained four independent OC-Plex 8 devices within the carrier, serving as imaging adaptors. Each OC-Plex 8 device housed eight independent chips with two channels: a gel channel for hydrogel and cell seeding and a medium channel for perfusion and immune cell flow. Notably, the absence of physical barriers between the two channels allowed direct cell–cell interactions between the compartments and cell migration. This configuration, coupled with live-cell imaging capabilities, provided an optimal platform for reconstituting the TME ex vivo and assessing the infiltration potential of T cells. BMDMs from FN1^fl/fl^ or FN1^ΔLyz2^ mice alone or mixed with LLC cells were seeded into the hydrogel matrix (gel channel), and CD8^+^ T-cells were circulated (perfusion channel; Fig. 3D). Co-cultured BMDMs and LLC cells synergistically promoted cell proliferation. However, FN1^fl/fl^ BMDMs had a stronger effect on tumor cell proliferation (Supplementary Fig. 4C). Meanwhile, more CD8^+^ T-cells infiltrated the hydrogel matrix in the FN1-deleted group, regardless of whether tumor cells were co-cultured (Supplementary Fig. 4D, E). This may be attributed to the chemokines secreted by FN1-deficient macrophages and reduced matrix rigidity.

To assess the effect of ECM stiffness on T-cell infiltration, rather than being influenced by chemokines, we added a high concentration of CXCL9—a potent T cell-recruiting chemokine^34^—into the hydrogel matrix to minimize differences in the chemotactic capacity between the two groups. Regardless of whether CXCL9 was added, T cells infiltrated more in the FN1-deleted group than in the control (Fig. 3E and Supplementary Fig. 4D, E). However, tumor cells exhibited a striking exclusion of T cells, even under chemotactic conditions with CXCL9 (Supplementary Fig. 4D).

To further assess the effect of FN1 on CD8^+^ T-cell infiltration in vivo, Dil-labeled activated CD8^+^ T cells were transferred into FN1^fl/fl^ and FN1^ΔLyz2^ mice through the tail vein, and LLC tumor grafts were imaged using an intravital microscopy platform 24 and 48 h after transfusion (Fig. 3F). Rare Dil^+^CD8^+^ T-cells were recruited to the tumors of FN1^fl/fl^ mice, while the number of infused Dil^+^CD8^+^ T-cells increased within 24–48 h of transfusion in FN1^ΔLyz2^ mice (Fig. 3G). The infiltration of other immune cells (including CD4^+^ T and NK cells) also increased in FN1^ΔLyz2^ tumors, indicating the formation of an immune-activated milieu (Fig. 3H). These findings demonstrate that TAM-derived FN1 enhances ECM stiffness in solid tumors and prevents lymphocyte infiltration, restraining antitumor immunity.

### FN1-induced F-actin bundling impairs glycolysis in macrophages

**Fig. 4:**
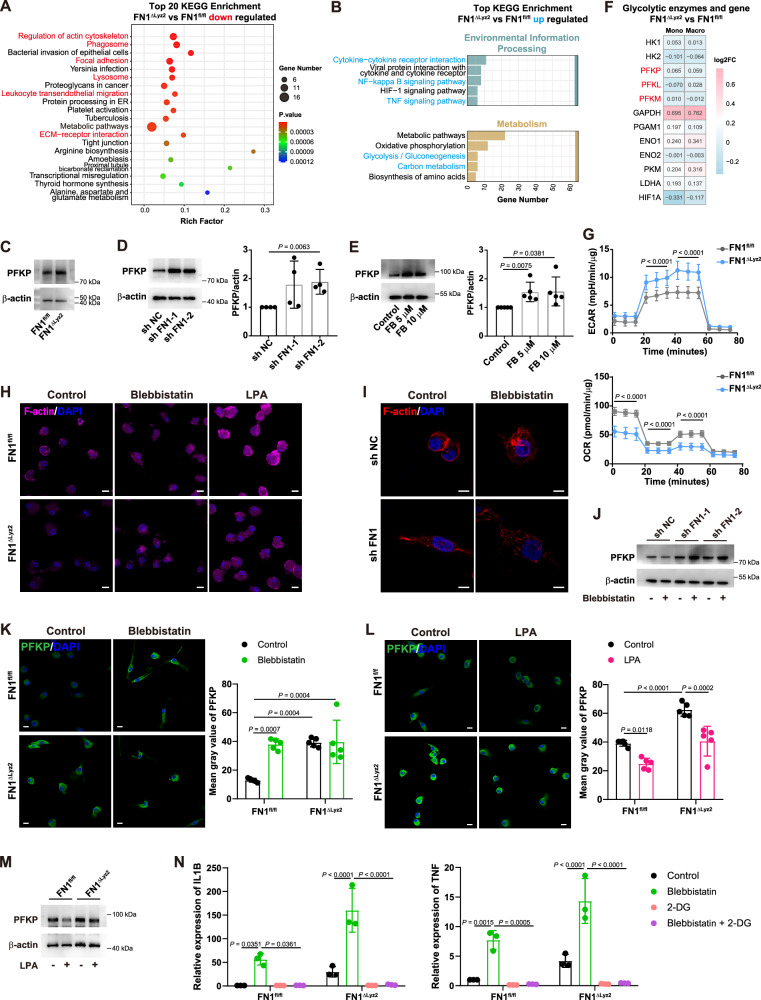
FN1 deficiency promotes glycolysis by reducing actin polymerization in macrophages. KEGG enrichment analysis of downregulated (**A**) and upregulated (**B**) differentially expressed genes from FN1^ΔLyz2^ macrophages compared to FN1^fl/fl^ macrophages. Hypergeometric test (**A**). **C** Representative western blotting image of PFKP expression in BMDMs from FN1^fl/fl^ and FN1^ΔLyz2^ mice. The experiment was repeated three times independently with similar results. **D** Representative western blotting image and relative analysis of PFKP expression in shNC and shFN1 THP-1-induced macrophages. *n* = 4 biologically independent samples. Data are displayed as the mean ± SD. two-tailed unpaired *t*-test. **E** Representative western blotting image and relative analysis of PFKP expression in MDMs treated with FB. *n* = 5 biologically independent samples. Data are displayed as the mean ± SD. two-tailed unpaired *t*-test. **F** Glycolysis-related gene expression in monocytes and macrophages from FN1^ΔLyz2^ mice compared to FN1^fl/fl^ mice, respectively. **G** ECAR and OCR in FN1^fl/fl^ and FN1^ΔLyz2^ BMDMs. *n* = 3 biologically independent samples. Data are displayed as the mean ± SD. two-way ANOVA. Representative immunofluorescence image of F-actin in FN1^fl/fl^ and FN1^ΔLyz2^ BMDMs treated with blebbistatin (40 μM) or LPA (20 μM) (**H**); shNC and shFN1 THP-1-induced macrophages treated with blebbistatin (40 μM) (**I**). Scale bar, 10 μm. The experiment was repeated three times independently with similar results. **J** Representative western blotting image of PFKP expression in shNC and shFN1 THP-1-induced macrophages treated with blebbistatin. The experiment was repeated three times independently with similar results. Representative immunofluorescence image of PFKP in FN1^fl/fl^ and FN1^ΔLyz2^ BMDMs treated with blebbistatin (**K**) and LPA (**L**). Scale bar, 10 μm. Quantification of mean fluorescence intensity. *n* = 5 biologically independent samples. Data are displayed as the mean ± SD. two-way ANOVA. **M** Representative western blotting image of PFKP expression in FN1^fl/fl^ and FN1^ΔLyz2^ BMDMs treated with LPA. The experiment was repeated three times independently with similar results. **N** qRT-PCR analysis of mRNA levels of IL1B and TNF in FN1^fl/fl^ and FN1^ΔLyz2^ BMDMs treated with 2-DG (10 mM), blebbistatin (40 μM), and 2-DG plus blebbistatin. *n* = 3 biologically independent samples. Data are displayed as the mean ± SD. two-way ANOVA. ECM extracellular matrix, BMDM bone marrow-derived macrophage, PFKP phosphofructokinase platelet, MDM monocyte-derived macrophage, ECAR extracellular acidification rate, OCR oxygen consumption rate, LPA lysophosphatidic acid, 2-DG 2-Deoxy-D-glucose.

To uncover the FN1-mediated intracellular pathways involved in TAM reprogramming, the TAM scRNA-seq data from FN1^fl/fl^ and FN1^ΔLyz2^ mice were screened. Kyoto Encyclopedia of Genes and Genomes (KEGG) and Gene Ontology (GO) analyses revealed that FN1^fl/fl^ TAMs were highly enriched in pathways involving the regulation of actin cytoskeleton organization, focal adhesion, and ECM-receptor interaction. In contrast, FN1-deficient TAMs were associated with pathways involved in glycolysis, carbon metabolism, chemokine activity, lymphocyte chemotaxis, inflammatory response, NF-κB, and TNF signaling pathways (Fig. 4A, B, and Supplementary Fig. 5A). KEGG pathway analysis of bulk RNA-seq data for shFN1 THP-1-induced macrophages revealed that the differentially expressed genes (DEG) were enriched in carbohydrate metabolism (Supplementary Fig. 5B, C). Hence, FN1 may induce cytoskeletal assembly, influencing cellular glycolysis and mediating an inflammatory phenotype.

Stiff ECM-induced stress fiber formation can reportedly protect the key rate-limiting metabolic enzyme of glycolysis—phosphofructokinase (PFK)—from degradation in epithelial cells^35^. Therefore, to evaluate the effect of FN1 on glycolysis, PFKP—the most abundant PFK type among the three isoforms (PFKP, PFKM, and PFKL)^36^,—was detected in different macrophage models, including BMDMs, THP-1-induced macrophages, and MDMs. PFKP expression was enhanced in macrophages when FN1 was blocked using FN1^ΔLyz2^ mice, shFN1, or an FN1 blocker (Fig. 4C–E). However, at the transcriptional level, PFKP was not upregulated after FN1 knockdown (Fig. 4F and Supplementary Fig. 5D–F). This suggests that FN1 may influence the post-transcriptional regulation of PFKP in macrophages.

Consistent with the elevated PFKP protein levels, extracellular acidification rate (ECAR), glycolysis, and the glycolytic metabolite lactate were also increased, accompanied by a decrease in oxygen consumption rate (OCR) in FN1-deficient macrophages (Fig. 4G and Supplementary Fig. 5G, H). Meanwhile, the loss of FN1 decreased actin filament (F-actin) organization (Fig. 4H, I). Similarly, blocking FN1 interfered with actin polymerization and increased PFKP activity (Supplementary Fig. 5I, J). Hence, blocking FN1 abrogated F-actin assembly and stimulated glycolysis in macrophages.

Treatment with blebbistatin—a specific myosin II inhibitor that interferes with F-actin formation^37^—enhanced PFKP generation (Fig. 4J, K). Similar results were observed in MDMs (Supplementary Fig. 5K, L). Consistent with the FN1-deficient macrophages, the mRNA expression of *Pfkp* did not increase, but lactate levels were upregulated in blebbistatin-treated MDMs (Supplementary Fig. 5M, N). In contrast, the F-actin agonist lysophosphatidic acid (LPA), which activates RHO signaling and stimulates stress fiber and focal adhesion formation^38^, attenuated PFKP expression in FN1^fl/fl^ and FN1^ΔLyz2^ BMDMs (Fig. 4L, M). Additionally, macrophages treated with blebbistatin exhibited higher levels of pro-inflammatory cytokines, including IL-1β and TNF (Supplementary Fig. 5O), consistent with the FN1-deficient macrophage results (Supplementary Fig. 2F, H, M, N). To further confirm the opposite relationship between macrophage glycolysis and immunosuppression, 2-deoxy-D-glucose (2-DG) was used to inhibit glycolysis. The elevated IL-1β and TNF in FN1^ΔLyz2^ BMDMs or with blebbistatin treatment significantly decreased in the 2-DG groups (Fig. 4N), demonstrating that glycolytic reprogramming of macrophages with blockade of FN1/F-actin axis promotes inflammatory cytokine production. Hence, FN1-induced F-actin bundling impaired glycolysis to mediate anti-inflammatory macrophages.

### FN1-induced actin polymerization impairs the RAC1-mTOR axis to promote autophagy-mediated glycolysis reduction

**Fig. 5:**
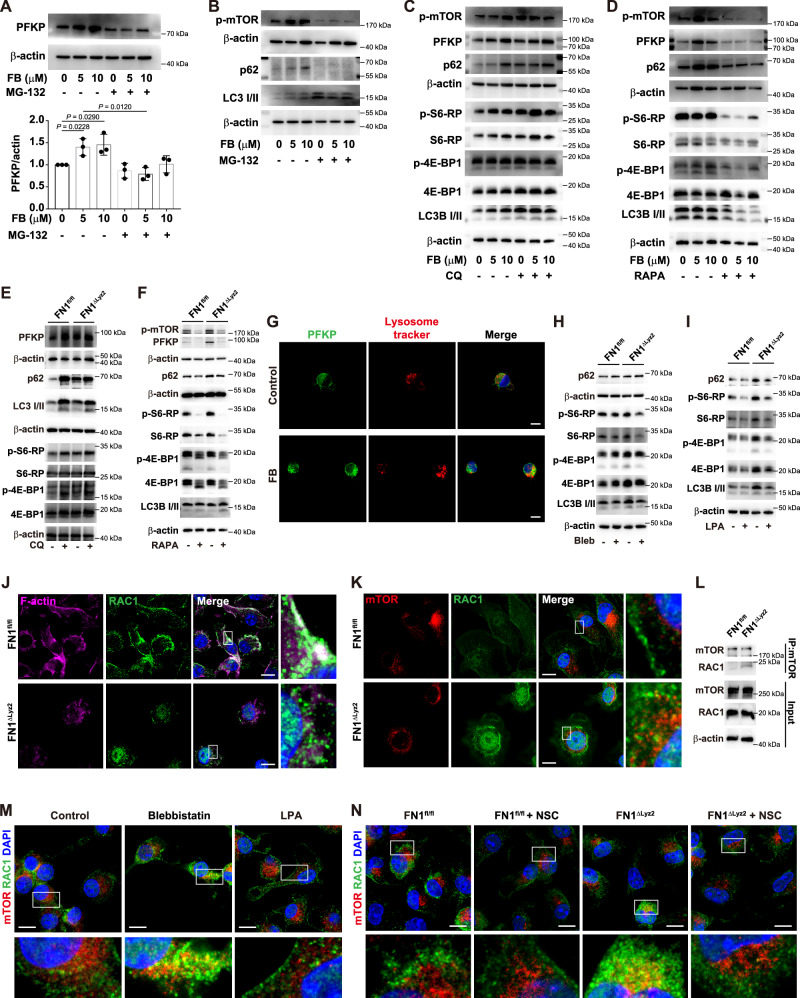
FN1 deficiency activates the RAC1-mTOR axis and attenuates autophagy to enhance glycolysis by reducing F-actin in macrophages. **A** Representative western blotting image and relative analysis of PFKP and β-actin in MDMs treated with MG-132 (5 μM). *n* = 3 biologically independent samples. Data are presented as the mean ± SD. two-tailed unpaired *t*-test. **B** Representative western blotting image of p-mTOR, p62, LC3I/II, and β-actin in MDMs treated with MG-132. The experiment was repeated three times independently with similar results. Representative western blotting image of PFKP, p-mTOR, p62, p-S6-RP, S6-RP, p-4E-BP1, 4E-BP1, LC3BI/II, and β-actin in MDMs treated with CQ (20 μM) (**C**) or RAPA (2 μM) (**D**). The experiment was repeated three times independently with similar results. Representative western blotting image of PFKP, p-mTOR, p62, p-S6-RP, S6-RP, p-4E-BP1, 4E-BP1, LC3BI/II, and β-actin in BMDMs from FN1^fl/fl^ and FN1^ΔLyz2^ mice treated with CQ (20 μM) (**E**) or RAPA (2 μM) (**F**). The experiment was repeated three times independently with similar results. **G** Representative immunofluorescence image of PFKP and lysosomes in MDMs treated with FB. Scale bar, 10 μm. The experiment was repeated three times independently with similar results. Representative western blotting image of p-mTOR, p62, p-S6-RP, S6-RP, p-4E-BP1, 4E-BP1, LC3BI/II, and β-actin in BMDMs from FN1^fl/fl^ and FN1^ΔLyz2^ mice treated with blebbistatin (40 μM) (**H**) or LPA (20 μM) (**I**). The experiment was repeated three times independently with similar results. **J** Representative immunofluorescence image of F-actin and RAC1 in BMDMs from FN1^fl/fl^ and FN1^ΔLyz2^ mice. Scale bar, 10 μm. The experiment was repeated four times independently with similar results. **K** Representative immunofluorescence image of mTOR and RAC1 in BMDMs from FN1^fl/fl^ and FN1^ΔLyz2^ mice. Scale bar, 10 μm. The experiment was repeated five times independently with similar results. **L** Representative immunoprecipitation and western blotting image of mTOR and RAC1 in BMDMs from FN1^fl/fl^ and FN1^ΔLyz2^ mice. Experiment was repeated three times independently with similar results. **M** Representative immunofluorescence image of mTOR and RAC1 in BMDMs from FN1^fl/fl^ mice treated with blebbistatin or LPA. Scale bar, 10 μm. The experiment was repeated five times independently with similar results. **N** Representative immunofluorescence image of mTOR and RAC1 in BMDMs from FN1^fl/fl^ and FN1^ΔLyz2^ mice treated with NSC-23766 (100 nM). Scale bar, 10 μm. The experiment was repeated three times independently with similar results. MDM monocyte-derived macrophage, BMDM bone marrow-derived macrophage, CQ chloroquine, RAPA rapamycin, LPA lysophosphatidic acid.

As PFKP is post-transcriptionally regulated, we hypothesized that the link between ECM stiffness, actin cytoskeleton organization, and glycolysis correlated with PFKP degradation. Common protein degradation pathways include the ubiquitin–proteasome and autophagy–lysosomal pathways. Given that PFKP undergoes proteasome-mediated degradation^39^, this was the first mechanism examined. Surprisingly, PFKP upregulation in FB-treated MDMs was reduced by MG-132—a well-known proteasome inhibitor (Fig. 5A). Similar results were observed for shFN1 THP-1-induced macrophages (Supplementary Fig. 6A). As most proteasome inhibitors, including MG-132, can enhance autophagy^40^, the effect of MG-132 on autophagy and PFKP expression was evaluated. Western blotting showed that p62 and p-mTOR levels decreased, while LC3II/I increased after MG-132 treatment, indicating that autophagy was enhanced. Meanwhile, p62 and p-mTOR levels increased in the FB-treated group, suggesting that blocking FN1 attenuated autophagy (Fig. 5B and Supplementary Fig. 6B). Thus, the increase in PFKP at the protein level after FN1 or F-actin ablation may have been due to decreased autophagy.

Subsequently, MDMs, THP-1, and BMDM models were treated with the autophagy–lysosome inhibitor chloroquine (CQ) and the autophagy agonist rapamycin (RAPA), respectively. Consistent with the MG-132 findings, the loss of FN1 significantly enhanced PFKP, p62, and p-mTOR levels, and CQ further improved these effects, while RAPA treatment markedly impaired PFKP, p62, and p-mTOR levels, even in FN1-deficient cells (Fig. 5C–F, Supplementary Fig. 6C–F). Confocal microscopy was further performed to validate these results. It was observed that PFKP was partially co-localized with lysosomes and autophagosomes. The loss of FN1 attenuated the colocalization of PFKP with lysosomes, while enhancing its colocalization with autophagosomes (Fig. 5G and Supplementary Fig. 6G, H). FN1-deficient BMDMs exhibited an increased number and larger size of LC3B puncta, indicating impaired autophagy (Supplementary Fig. 6H). These findings suggest that FN1 deficiency reduces PFKP degradation by inhibiting autophagy.

To determine whether FN1 enhanced autophagy by promoting actin skeleton organization, the changes in autophagy after interference with F-actin were evaluated. Blebbistatin increased the expression of p62 and p-mTOR, whereas it decreased that of LC3II/I (Fig. 5H, Supplementary Fig. 6I), indicating impaired autophagy. Similarly, treatment with Blebbistatin attenuated the colocalization of PFKP with lysosomes (Supplementary Fig. 6J). Meanwhile, LPA reversed p62 and p-mTOR expression and upregulated LC3II/I in FN1^fl/fl^ and FN1^ΔLyz2^ BMDMs or MDMs (Fig. 5I, Supplementary Fig. 6K). This indicated that FN1 promoted F-actin aggregation and autophagy. To further validate the extracellular FN1-triggered signals through its receptor integrin, we employed ATN-161, an integrin α5β1 and αvβ3 antagonist that blocks FN1-integrin binding^41^. ATN-161 treatment significantly increased PFKP, p62 and decreased LC3II/I (Supplementary Fig. 6L), indicating that integrin blockade recapitulated the metabolic and autophagy phenotypes induced by FN1 knockout. Thus, the F-actin organization induced by FN1-integrin interaction promotes the autophagic digestion of PFKP in macrophages, resulting in lower glycolysis.

The mTORC1 complex (comprising mTOR, Raptor, PRAS40, mLST8, and DEPTOR) serves as a key regulator of autophagy, wherein the activation of mTOR in particular inhibits various steps in this process^42^. The loss of FN1 significantly increased the phosphorylation of mTOR (Ser2448), RPS6/S6 (Ser235/236), and 4E-BP1 (Thr37/46)—substrates of the mTOR pathway, which were improved by CQ/Blebbistatin/ATN-161 treatment and reversed by RAPA/LPA treatment (Fig. 5C–F, H, I, Supplementary Fig. 6L), indicating that FN1-integrin interaction promoted F-actin organization to enhance autophagy through mTOR inactivation. To explore how F-actin organization attenuated mTOR phosphorylation, it was hypothesized that RAC1 may act as a critical intermediary, given its dual roles in actin cytoskeleton remodeling^43^ and mTOR signaling activation^44^. We found that RAC1 co-localized with F-actin, which was located primarily in the cytoplasmic membrane region of FN1^fl/fl^ BMDMs, but was released to the cytoplasm in the form of FN1^ΔLyz2^ BMDMs (Fig. 5J, Supplementary Fig. 7A). Correspondingly, there was little co-localization of RAC1 with mTOR in FN1^fl/fl^ BMDMs, whereas co-localization sites tended to increase significantly in the case of FN1^ΔLyz2^ BMDMs (Fig. 5K, Supplementary Fig. 7B). Immunoprecipitation further confirmed that there was more RAC1 binding to mTOR in the FN1 deletion group (Fig. 5L). Furthermore, the elevated RAC1-mTOR co-localization enrichment in the cytoplasm regions appeared under the Blebbistatin treatment. Nevertheless, the co-localization of RAC1 with mTOR decreased, while RAC1 showed increased localization in the cytoplasmic membrane regions following treatment with LPA (Fig. 5M, Supplementary Fig. 7C). Consistently, ATN-161 treatment decreased RAC1 co-localization with F-actin while increasing its co-localization with mTOR (Supplementary Fig. 7D). These facts supported the hypothesis that FN1-integrin interaction induced competitive binding of F-actin to RAC1, thereby impeding mTOR phosphorylation. To further ascertain whether the heterogeneously localized RAC1 is involved in the mTOR activation, the RAC1 GTPase inhibitor NSC-23766 was employed to inhibit RAC1 activation. Consequently, the colocalization signals of FN1^ΔLyz2^ BMDMs were significantly decreased and showed no difference with FN1^fl/fl^ BMDMs upon NSC-23766 treatment (Fig. 5N, Supplementary Fig. 7E, F). Meanwhile, the elevated p-mTOR in FN1^ΔLyz2^ BMDMs was decreased via RAC1 inhibition (Supplementary Fig. 7G). Therefore, these data suggest that FN1 can block the RAC1-dependent mTOR activation by trapping active RAC1 within the F-actin bundles, thereby promoting autophagic degradation of PFKP.

### Ablation of FN1 attenuates autophagy and enhances PFK to improve antitumor immunity

**Fig. 6:**
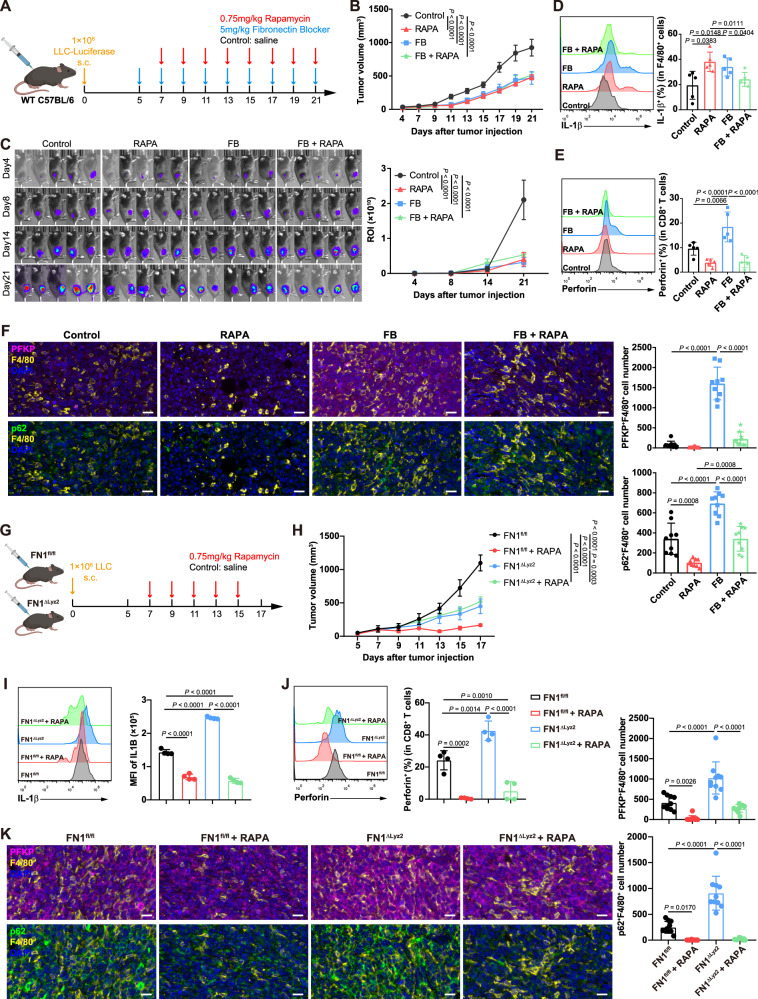
FN1 deficiency impairs autophagy and increases PFK in vivo. **A** Schematic diagram of the experimental approach; 1 × 10^6^ Luciferase-LLC cells were subcutaneously (s.c.) implanted into C57BL/6J mice. On Day 7, mice were administered FB (5 mg/kg), RAPA (0.75 mg/kg), or FB plus RAPA, intraperitoneally (i.p.) every two days. *n* = 5 biologically independent samples. Created in BioRender. Li, Y. (2026) https://BioRender.com/vk6cajb. **B** Tumor growth was measured every two days. Data are displayed as the mean ± SEM. two-way ANOVA. **C** Tumor growth monitored by bioluminescence imaging. Data are displayed as the mean ± SEM. two-way ANOVA. **D** Flow cytometry analysis of IL-1β expression in F4/80^+^ macrophages. *n* = 5 biologically independent samples. Data are displayed as the mean ± SD. two-tailed unpaired *t*-test. **E** Flow cytometry analysis of perforin expression in the CD8^+^ T-cells of tumor tissues. *n* = 5 biologically independent samples. Data are displayed as the mean ± SD. one-way ANOVA. **F** Representative mIHC image of PFKP and p62 expression in F4/80^+^ macrophages of tumor tissues. Scale bar, 20 μm. Quantification of cell number in each group. *n* = 3 biologically independent samples. Each dot represents one ROI. Data are displayed as the mean ± SD. one-way ANOVA. **G** Schematic diagram of the experimental approach; 1 × 10^6^ Luciferase-LLC cells were s.c. implanted into FN1^fl/fl^ and FN1^ΔLyz2^ mice. On Day 7, mice were administered RAPA (0.75 mg/kg, i.p.) every two days. *n* = 4 biologically independent samples. Created in BioRender. Li, Y. (2026) https://BioRender.com/vk6cajb. **H** Tumor growth was measured every two days. Data are displayed as the mean ± SEM. two-way ANOVA. Flow cytometry analysis of IL-1β expression in F4/80^+^ macrophages (**I**), and perforin expression in CD8^+^ T-cells (**J**) in tumor tissues, *n* = 4 biologically independent samples. Data are displayed as the mean ± SD. one-way ANOVA. **K** Representative mIHC image of PFKP and p62 expression in F4/80^+^ macrophages of tumor tissues. Scale bar, 20 μm. Quantification of cell number in each group. *n* = 3 biologically independent samples. Each dot represents one ROI. Data are displayed as the mean ± SD. one-way ANOVA. s.c. subcutaneously, i.p. intraperitoneally, FB fibronectin blocker, IL interleukin, ROI region of interest.

To determine whether FN1-induced mechanical changes and functional characteristics in anti-inflammatory macrophages occurred in vivo, C57BL/6 J mice were subcutaneously (s.c.) injected with LLC, followed by intraperitoneal (i.p.) injections of RAPA (0.75 mg/kg) or FB (5 mg/kg)^45,46^ alone or in combination every two days (Fig. 6A). Tumor growth was reduced in all treatment groups; however, RAPA combined with FB did not further impair tumor progression compared with the FB group (Fig. 6B, C). Depletion of F4/80^+^ macrophages was also observed in all treatment groups (FB, RAPA, and FB + RAPA), with no significant differences among them (Supplementary Fig. 8A, B). Consistent with in vitro findings, blockade of FN1 induced a higher proportion of IL-1β^+^ and TNF^+^ macrophages in tumors and the spleen. These effects were attenuated in the RAPA combination group (Fig. 6D, Supplementary Fig. 8C, D). Additionally, the proportion of perforin^+^CD8^+^ T and granzyme B^+^CD8^+^ T-cells increased in FB-treated mice, whereas RAPA significantly decreased the antitumor response (Fig. 6E, Supplementary Fig. 8E). Consistently, blocking FN1 upregulated PFKP and p62 in macrophages; this was significantly inhibited by RAPA treatment (Fig. 6F). These results suggest that FN1 is an upstream activator of autophagy that limits glycolysis and inflammation in macrophages, promoting the formation of an immunosuppressive TME.

To further verify these results, FN1^fl/fl^ and FN1^ΔLyz2^ mice were s.c. injected with LLC; RAPA (0.75 mg/kg) was administered every two days from Day 7 after cell implantation (Fig. 6G). Tumor growth was slower in FN1^ΔLyz2^ mice than in FN1^fl/fl^ mice (Fig. 6H). However, RAPA administration did not further reduce the tumor size in FN1^ΔLyz2^ mice, but it markedly influenced the FN1^fl/fl^ group (Fig. 6H). Similar to FB-treated mice, higher expression of IL-1β was observed in FN1^ΔLyz2^ macrophages compared to their littermate controls, which was reduced by RAPA (Fig. 6I). In addition, the proportions of perforin^+^, granzyme B^+^, and TNF^+^CD8^+^ T-cells were significantly increased in FN1^ΔLyz2^ tumors. This was reversed in the RAPA group, accompanied by reduced PD1^+^CD8^+^ T-cells in all RAPA and FN1^ΔLyz2^ groups (Fig. 6J, Supplementary Fig. 8F). Hence, FN1-deficient macrophages systemically increased the cytotoxic effects of T cells, which were blunted by activated autophagy. Furthermore, mIHC results showed higher PFKP and p62 expression in FN1^ΔLyz2^ macrophages than in FN1^fl/fl^ tissues; this was also reduced in the RAPA-treated group (Fig. 6K). These results support the idea that ablating FN1 attenuates autophagy and enhances PFK to improve antitumor immunity.

### Blocking FN1 synergizes with immune checkpoint blockade therapy to enhance antitumor responses

**Fig. 7:**
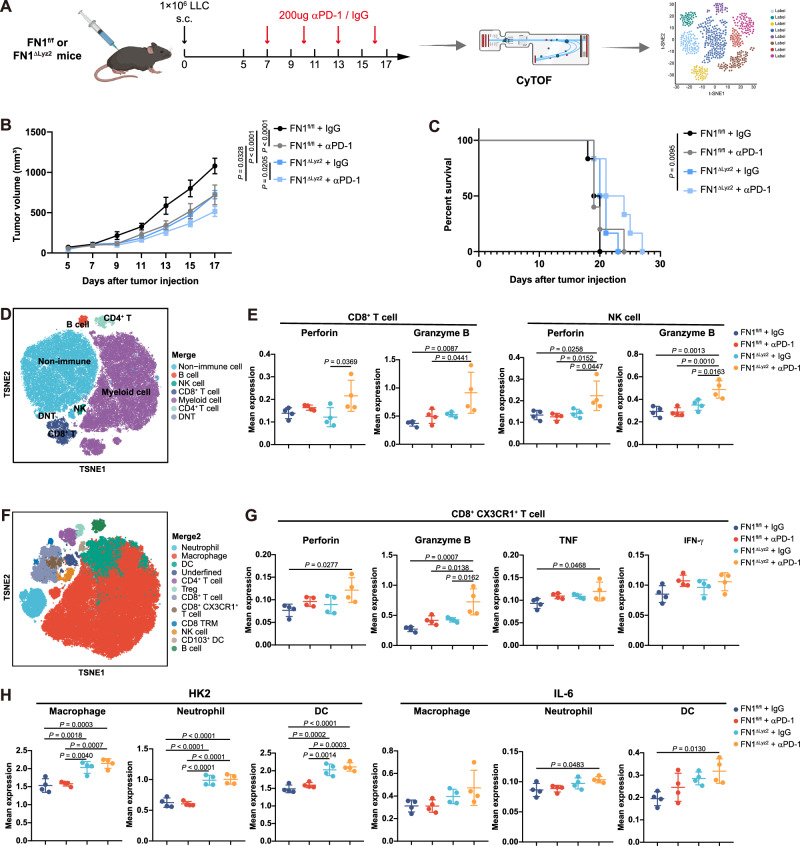
FN1 deficiency improves antitumor response and efficacy of ICB therapy. **A** Schematic diagram of the experimental approach; 1 × 10^6^ Luciferase-LLC cells were s.c. implanted into FN1^fl/fl^ and FN1^ΔLyz2^ mice. On Day 7, mice were administered anti-PD-1 antibody intraperitoneally (200 μg per mouse) every three days. *n* = 4 biologically independent samples. Created in BioRender. Li, Y. (2026) https://BioRender.com/vk6cajb. **B** Tumor growth was measured every two days. *n* = 5 biologically independent samples for FN1^fl/fl^ + anti-PD-1 group, *n* = 6 biologically independent samples for other groups. Data are displayed as the mean ± SEM. two-way ANOVA. **C** Survival curve of mice under different treatments. *n* = 5 biologically independent samples for FN1^fl/fl^ + anti-PD-1 group, *n* = 6 biologically independent samples for other groups. log-rank test. **D** tSNE plots of immune and non-immune cells from mass cytometry data. **E** Mass cytometry analysis of perforin and granzyme B expression in CD8^+^ T and NK cells. *n* = 4 biologically independent samples. Data are displayed as the mean ± SD. one-way ANOVA. **F** tSNE plots of immune cells from mass cytometry data. **G** Mass cytometry analysis of perforin, granzyme B, TNF, and IFN-γ expression in CX3CR1^+^CD8^+^ T-cells. *n* = 4 biologically independent samples. Data are displayed as the mean ± SD. one-way ANOVA. **H** Mass cytometry analysis of IL-6 and HK2 expression in macrophages, neutrophils, and DCs, respectively. *n* = 4 biologically independent samples. Data are displayed as the mean ± SD. one-way ANOVA. ICB, immune checkpoint blockade; s.c. subcutaneously, PD-1 programmed cell death protein 1, TNF tumor necrosis factor, IFN-γ interferon-gamma, IL interleukin, HK2 hexokinase 2, DC dendritic cell.

As FN1-deficient macrophages reduce ECM stiffness and recruit CD8^+^ T-cells to the tumor niche, the effect of blocking FN1 on ICB therapy efficacy was evaluated. FN1^fl/fl^ and FN1^ΔLyz2^ LLC xenograft mouse models were administered an anti-PD-1 antibody or IgG (i.p., 200 ug per mouse). After euthanasia, cytometry by the time of flight (CyTOF) was employed to analyze tumor tissue samples (Fig. 7A). FN1^ΔLyz2^ mice treated with the anti-PD-1 antibody exhibited superior antitumor efficacy and longer survival without weight loss (Fig. 7B, C, 9A). Six immune cell populations were identified: myeloid, B, CD4^+^ T, CD8^+^ T, double-negative T, and NK cells (Fig. 7D, Supplementary Fig. 9B). Higher proportions of CD8^+^ T and NK cells and lower percentages of non-immune cells were observed in the PD-1 blockade FN1^fl/fl^, FN1^ΔLyz2^, and non-treated FN1^ΔLyz2^ mouse groups (Supplementary Fig. 9C). FN1^ΔLyz2^ mice treated with the anti-PD-1 antibody displayed the highest perforin and granzyme B expression in CD8^+^ T and NK cells (Fig. 7E). Thus, FN1 deficiency markedly enhanced the cytotoxic efficacy of PD-1 blockade. Moreover, in non-immune cells, the expression of Ki67 decreased in FN1^ΔLyz2^ mice with PD-1 blockade treatment, indicating that tumor proliferation was impaired (Supplementary Fig. 9D). The increased expression of the “don’t eat me” molecules PD-L1 and CD47 in FN1^fl/fl^ mice treated with the anti-PD-1 antibody was inversed in FN1^ΔLyz2^ mice with or without anti-PD-1 therapy (Supplementary Fig. 9D).

The subpopulations of immune cells were further evaluated (Fig. 7F, Supplementary Fig. 9E). A larger proportion of CX3CR1^+^CD8^+^ T-cells—a powerful cytotoxic CD8^+^ T-cell subset^47^—infiltrated the TME of the PD-1 blockade or FN1^ΔLyz2^ groups (Supplementary Fig. 9F). The highest levels of IFN-γ, TNF, perforin, and granzyme B in CX3CR1^+^CD8^+^ T-cells were observed in FN1^ΔLyz2^ mice treated with the anti-PD-1 antibody (Fig. 7G). Hence, macrophage-derived FN1 integrated with the stiff ECM to augment CD8^+^ T-cell exclusion and exhaustion in the TME.

Myeloid cells were further subdivided into three groups: neutrophils, macrophages, and DCs (Fig. 7F, Supplementary Fig. 9E). The inflammatory cytokine IL-6 and the first enzyme of glycolysis, hexokinase 2 (HK2), were highly expressed in all myeloid cell subsets of FN1^ΔLyz2^ mice, particularly FN1^ΔLyz2^ mice treated with anti-PD-1 therapy (Fig. 7H). This suggested that ablation of FN1 signaling in macrophages increased cellular glycolysis and produced more pro-inflammatory cytokines. This pathway may be conserved in other myeloid cells.

Collectively, these data demonstrated that FN1-deficient macrophages could reshape the NSCLC TME from a pro-tumoral to antitumoral status by reducing tissue stiffness and recruiting cytotoxic T and NK cells, creating an overall antitumor immune response in synergy with ICB therapy.

## Discussion

Stiff ECM forms a physical barrier that limits lymphocyte infiltration and mediates tumor cell proliferation^6,48^. Meanwhile, the doom driven by immunosuppressive cells collaborates to promote tumor progression and limit the efficacy of immunotherapy. TAMs, as a core member of the immunosuppressive milieu, have also been evaluated for their ability to remodel the ECM^13–15^. However, the direct effect of TAMs on tumor stiffness and its connection to immunosuppressive function remain poorly understood. Here, we report that TAM-derived FN1 contributes to ECM stiffness, drives immune suppression, and promotes the depletion of tumor-killing cells in the TME, thus providing new insights into the role of TAMs in tumor immune escape.

FN1 is an important macromolecular protein in the ECM network, which works as a “gel” to interact with other cells and components. Past research revealed that FN1 promotes fibrin binding and ECM remodeling in the premetastatic niche^24,49^. In this study, we identified FN1^+^ macrophages assembling the ECM by secreting FN1 and inducing the expression of collagen-associated genes. Recent studies have reported the vital role of TAMs in promoting the immune-excluded desmoplastic structure and decreasing T-cell infiltration, including macrophage–myofibroblast transition and deposition of collagen^13,15,50^. Thus, our findings suggest that TAM-derived FN1 increases the stiffness of tumor tissues and hinders the infiltration of effector cells into the TME.

FN1 is also considered an indicator of M2-like macrophages^26–28,51^; FN1 inhibits NF-κB signaling in macrophages by activating the integrin-mediated focal adhesion kinase (FAK)-phosphatidylinositol-3 kinase γ (PI3Kγ) pathway^52^. In the current study, FN1 loss increased essential inflammatory factors in macrophages, including CXCL9 and CXCL10. This facilitated the recruitment of tumor-killing cells to reshape the antitumor immune microenvironment. The strong pro-inflammatory response was consistent with increased glycolysis in FN1-deficient macrophages. As reported, classically activated M1-like macrophages exhibit an inflammatory response that is highly dependent on glycolysis^20^. Mechanistically, we revealed that FN1 promotes the aggregation of the actin cytoskeleton, inducing autophagy-mediated degradation of the rate-limiting enzyme PFK, which limits glycolysis and the secretion of inflammatory factors by macrophages. This is consistent with recent reports that macrophages can be reprogrammed to an anti-inflammatory phenotype through stiffness sensing and efferocytosis on stiff ECM^53^. Moreover, activation of the LPA–autophagy axis facilitates the M2 differentiation of decidual macrophages during normal pregnancy^54^. These results suggest that FN1 deficiency in macrophages has the potential to transform “cold” tumors into “hot” tumors, enhancing immune cell infiltration and cytotoxicity. Further research is needed to verify whether this phenomenon exists in other solid tumors, especially liver cancer and gliomas, where tissue-resident macrophages are abundant.

Similar stiffness-induced autophagy mechanisms have also been described in other TME cell types. Specifically, Hupfer et al. demonstrated that CAFs sense ECM stiffness through the integrin αV-focal adhesion kinase module, leading to AMPKα stabilization at focal adhesions and subsequent autophagy induction to support cancer cell growth^55^. In our study, CyTOF analysis revealed elevated glycolytic enzymes and inflammatory cytokines in macrophages, neutrophils, and DCs from FN1^ΔLyz2^ mice, suggesting that stromal cells may share conserved stiffness-autophagy adaptation mechanisms. These provide a more comprehensive framework for understanding how ECM stiffness regulates autophagy and metabolism across distinct cellular populations, further highlighting the therapeutic potential of targeting shared stiffness-sensing pathways across TME cell types, offering new avenues for intervention in mechanobiology-driven disease processes.

Autophagy regulates the phenotype and function of macrophages. A study on glioma found that exosomes produced by tumor cells under hypoxic conditions significantly promoted macrophage autophagy and polarization toward M2^56^. In the HCC microenvironment, tumor cells activate the TLR2 signaling pathway to restrict NF-κB activity via selective autophagy in TAMs^57^. Defects in LC3-associated phagocytosis induce pro-inflammatory gene expression in TAMs and trigger STING-mediated type I interferon responses that promote T-cell antitumor immunity^58^. In our study, the loss of FN1 mediated the disassembly of the F-actin bundles and reduced autophagy (as reflected by the upregulated p-mTOR and p62) and was associated with pro-inflammatory macrophages. mTOR is central to cellular metabolism, promoting anabolic processes and inhibiting catabolic processes such as autophagy^59^. While no direct reports implicating F-actin in the regulation of mTOR exist, Li et al. demonstrated that GTPase-RAC1 binds to lysosomal mTOR, thereby promoting its phosphorylation^44^. Moreover, RAC1 plays a critical role in actin dynamics^43,60^. Our results revealed the mechanism by which FN1 engaged in trapping RAC1 within the F-actin bundles. This resulted in decreased mTOR phosphorylation, thereby relieving mTOR-mediated autophagy inhibition. mTOR-induced HIF-1α expression causes macrophages to undergo a metabolic switch to glycolysis^61,62^. However, scRNA-seq analysis in the current study did not reveal increased mRNA expression of HIF-1α or PFK in FN1-deficient macrophages. Considering the upregulated glycolysis and the inconsistency between PFKP at the protein and transcript levels in three macrophage models, FN1 may influence the post-transcriptional regulation of glycolytic enzymes. In contrast to ubiquitin-mediated proteasome degradation in epithelial cells^35^, this study reveals macrophages regulate glycolysis by degrading PFK through autophagy.

Our data showed that rapamycin (RAPA) alone suppressed tumor growth; this effect arises because rapamycin, as an mTOR inhibitor, can interfere with mRNA transcription, protein synthesis, and cell cycle transition, inducing autophagy and apoptosis in tumor cells, and inhibiting pro-oncogenic pathways (e.g., EGFR/MET), thereby exerting antitumor effects^63,64^. However, RAPA combined with FN1 blockade did not further impair tumor progression compared with the monotherapy groups (Fig. 6B, C, H). This suggests that inhibiting FN1 may have an opposite effect on activating autophagy, which needs further exploration. In addition, RAPA treatment alone significantly suppressed T cell functionality, which might be because rapamycin itself can inhibit the activation and proliferation of CD8^+^ T cells and reduce the differentiation of T cells into effector phenotypes, thus can be used for immunosuppression after organ transplantation^65,66^. This highlights a “double-edged sword”: while rapamycin kills tumor cells, it simultaneously impairs antitumor immunity, underscoring the need for cell-specific targeting.

Previous studies demonstrated that tumor-stiff niches promote CD8^+^ T-cell exhaustion^11^, and an immunosuppressive milieu restrains antitumor immunity^67^. Thus, FN1 depletion enhances antitumor immunity both by reducing ECM stiffness and impairing the suppressive niche. scRNA-seq analysis revealed enhanced antitumor responses of CD4, CD8, and NK cells in FN1^ΔLyz2^ tumors. In addition, administering an anti-PD-1 antibody to FN1^ΔLyz2^ mice slowed tumor growth and improved survival. These results indicate that FN1 depletion in macrophages enhances the efficacy of ICB therapy.

Our research provides valuable insights into the regulatory role of TAM-derived FN1 in facilitating tumor ECM stiffness and immunosuppressive function, thereby promoting tumor progression. However, several issues still need to be addressed. Previous studies have indicated that myeloid cells exhibit high levels of glycolysis in the TME, and tumor cells also utilize the Warburg effect to supply energy^68,69^. Our findings suggest that FN1 deficiency upregulates glycolysis in macrophages. Whether this contributes to enhanced glucose competition between TAMs and tumor cells, thereby reducing their tumor-promoting effects, warrants further exploration. While FN1 deficiency in macrophages enhanced ICB efficacy, the increased glycolysis does lead to a hypoxic environment, which may also explain why tumor progression was not fully reversed. This may be due to the hypoxic environment, caused by elevated glycolysis, which limits the antitumor response^70^. Whether hypoxic restriction and lactate accumulation can be counterbalanced by a combination of drugs that reduce hypoxic TAMs remains to be investigated.

In summary, macrophage-derived FN1 plays a key role in ECM stiffening and shaping the immunosuppressive milieu in NSCLC. By linking ECM regulation to TAM metabolism and function, this study explored the extracellular and intracellular effects of macrophage-derived FN1, suggesting that targeting FN1 may be a promising strategy for enhancing immunotherapy efficacy.

## Methods

### NSCLC specimens

Sixteen patients with NSCLC were enrolled in this study at The First Affiliated Hospital of Zhengzhou University. The samples were used after obtaining informed consent from the patients. The study was approved by the Scientific Research and Clinical Trial Ethics Committee of the First Affiliated Hospital of Zhengzhou University (No. 2019-KY-256) and patients provided written informed consent. Clinical information related to the patients is summarized in Supplementary Table 1. Ten samples were used for scRNA-seq and immunohistochemistry analyses, and nine were subjected to flow cytometry.

### Mice

The FN1^fl/fl^ and FN1^ΔLyz2^ mice were purchased from Cyagen (Suzhou, China). Macrophage-specific FN1 knockout mice (FN1^ΔLyz2^, heterozygous for Lyz2-Cre) were generated by crossing FN1^fl/fl^ mice (Cyagen, S-CKO-02465) with Lyz2-cre mice (Cyagen, C001003), and their F1 heterozygotes were crossed with FN1^fl/fl^ mice. To minimize environmental variables, we utilized littermate controls generated by crossing FN1^fl/fl^ with FN1^ΔLyz2^ mice (heterozygous for Lyz2-Cre), yielding litters with approximately 50% FN1^fl/fl^ and 50% FN1^ΔLyz2^ pups. All mice were genotyped by PCR and agarose gel electrophoresis using genomic DNA extracted from tail biopsy tissues. All experiments employed age-matched (6–8 weeks old) and sex-matched littermates housed in the same facility. C57BL/6 mice (Charles River, 219) aged 6–8 weeks were purchased from Charles River (Beijing, China). Experimental mice and control mice were housed separately during the experiment. All mice were housed at 21 ± 2 °C and 40–60% humidity, on a 12 h light/dark cycle in a specific pathogen-free facility, and received humane care according to animal welfare guidelines throughout the study. All animal experiments were approved by the Laboratory Animal Welfare Ethics Committee of Zhengzhou University Laboratory Animal Center (No. ZZU-LAC20211224 [18]).

### Cells

CD14^+^ monocytes were isolated from the peripheral blood mononuclear cells (PBMCs) of healthy donors using anti-CD14 microbeads (Miltenyi Biotec, 130-050-201). Mouse BMDMs were obtained by flushing the femurs. Mouse CD8^+^ T cells were dissociated from spleens using the MojoSort Mouse CD8 T Cell Isolation Kit (BioLegend, 480035) through magnetic cell sorting. The human monocyte THP-1 cell line (National Collection of Authenticated Cell Cultures, TCHu57) was cultured in Roswell Park Memorial Institute (RPMI)-1640 medium supplemented with 10% fetal bovine serum (FBS, Lonsera, S711-001S) and 100 U/mL penicillin/streptomycin. The LLC cell line (Wuhan Pricella Biotechnology Co., Ltd, CL-0140) was transfected with a lentivirus generated by co-packaging pCDH-EF1-Luc2-P2A-copGFP (Addgene, 72485) with the packaging plasmid psPAX2 (Addgene, 12260) and envelope plasmid pMD2.G (Addgene, 12259). LLC was maintained in Dulbecco’s Modified Eagle’s medium (DMEM)-high glucose supplemented with 10% FBS and 100 U/mL penicillin/streptomycin. Cells were cultured at 37 °C with 5% CO_2_.

### LLC mouse model

Luciferase-LLC (1 × 10^6^ cells) were subcutaneously implanted into FN1^fl/fl^, FN1^ΔLyz2^, and C57BL/6 mice. After seven days, FN1^fl/fl^ and FN1^ΔLyz2^ mice were injected with anti-PD-1 (Selleck, A2122, https://www.selleckchem.com/)/IgG antibody (Selleck, A2123) (200 μg per mouse) every three days. C57BL/6 mice were injected with FB (5 mg/kg; Santa Cruz Biotechnology, sc-202156)/RAPA (0.75 mg/kg; Selleck, S1039)/PBS every two days. FN1^fl/fl^ and FN1^ΔLyz2^ mice were injected with RAPA/PBS. Tumor growth was monitored by measuring body weight and tumor size, and via bioluminescence imaging. The average radiance (p/sec/cm²/sr) within the region of interest was analyzed using Living Image v4.4 software. Tumor size was measured using digital calipers, and tumor volume was calculated using the formula: volume = (length × width²)/2. Tumor growth and overall animal health were monitored every 2–3 days. In accordance with the guidelines of the Laboratory Animal Welfare Ethics Committee of Zhengzhou University Laboratory Animal Center, the maximum allowable tumor size was 1500 mm³, and this limit was not exceeded in any of the experiments. Mice were humanely euthanized if tumor volume exceeded the ethical limit, if body weight loss exceeded 20%, or if animals showed signs of ulceration, impaired mobility, or severe distress. All euthanasia procedures were performed using CO₂ inhalation followed by cervical dislocation in accordance with animal welfare guidelines.

### Preparation of cell suspensions from human and mouse tumor tissues

Freshly resected human tumor samples were placed on ice and transported to the laboratory within 2 h for dissection and processing. The tissue samples were thoroughly washed with pre-cooled PBS to remove visible blood clots. Tumor samples were mechanically minced into small pieces with a sterile scalpel and further dissociated into single-cell suspensions using a Tumor Dissociation Kit (Miltenyi Biotec, 130-095-929) following the manufacturer’s instructions. The suspensions were filtered through a 70 μm strainer and centrifuged at 300 × g for 10 min at 4 °C. Cell count and viability were estimated using a fluorescence Cell Analyzer (Countstar Rigel S2) with AO/PI reagent after removal of erythrocytes. Finally, the suspensions were washed twice with PBS and diluted to the appropriate concentrations for flow cytometry, library construction, and sequencing. Mouse tumor tissues were disrupted into pieces and incubated with a tumor dissociation kit (Miltenyi Biotec, 130-096-730) for 30 min at 37 °C. Subsequently, the suspensions were filtered through a 70 μm strainer and centrifuged at 500 × *g* for 5 min at 4 °C. The cell suspension was prepared using a procedure similar to that described above.

### TAM polarization

Human CD14^+^ monocytes from healthy donors, THP-1 cells, and mouse BMDMs were used for TAM polarization^71–74^. CD14^+^ monocytes were maintained in 10% FBS RPMI-1640 medium containing 20 ng/mL macrophage colony-stimulating factor (M-CSF; PeproTech, 300-25-100UG) on Days 0, 1, 3, and 5, and changed to medium containing 20 ng/mL IL-4 (PeproTech, 200-04-20UG) on Day 7. THP-1 cells were induced in complete medium containing 100 ng/mL PMA (Selleck, S7791) for 24 h, washed with PBS, and transferred to complete medium for 48 h. The cells were subsequently induced in medium containing 40 ng/mL IL-4 for 24 h. BMDMs were cultured in 10% FBS RPMI-1640 containing 10 ng/mL M-CSF (PeproTech, 315-02-100UG) on Days 0 and 3 and changed to medium containing 10 ng/mL IL-4 (PeproTech, 214-14-100UG) and IL-13 (PeproTech, 210-13-10UG) on Day 5. Subsequently, agonists and antagonists, including Fibronectin inhibitor (FB, Santa Cruz Biotechnology, sc-202156, 5 μM or 10 μM), Blebbistatin (Selleck, S7099, 20 μM or 40 μM), Lysophosphatidic acid (LPA, Santa Cruz Biotechnology, sc-201053, 20 μM), 2-deoxy-D-glucose (2-DG, Selleck, S4701, 10 mM), MG-132 (Selleck, S2619, 5 μM), Chloroquine (CQ, Selleck, S6999, 20 μM), Rapamycin (RAPA, Selleck, S1039, 2 μM), NSC-23766 (Selleck, S8031, 100 nM) and ATN-161 (Selleck, S8454,10μM) were added to the medium for 24 h.

### scRNA-seq

The scRNA-seq libraries of human samples were generated using the SeekOne Digital Droplet Single Cell 5′ library preparation kit (SeekGene, K00501)^75^. Briefly, more than 10,000 live cells were added to the sample wells in a SeekOne DD Chip S3 with a reverse transcription reagent.

Barcoded hydrogel beads and partitioning oil were added to generate emulsified droplets, and reverse transcription was performed. The obtained cDNA was purified and amplified by PCR. Following fragmentation, end-repair, and A-tailed indexed PCR were performed to amplify the cDNA. Purified libraries were sequenced on an Illumina NovaSeq 6000 platform.

The scRNA-seq libraries of mouse tumors were generated using the MobiCube Single-Cell 3’RNA-seq Kit (Mobidrop, SOP-05-1001 v2.1)^76^. Briefly, the cell suspension was loaded into the MobiNova instrument, where gel beads containing barcode information were mixed with the cell and enzyme and encapsulated in oil surfactant droplets to form GEMs (Gel Beads-In-Emulsions). The gel beads were dissolved to release the barcode sequences and reverse transcribed. The cDNA was amplified using PCR. The products of all GEMs were pooled to construct a standard sequencing library. Purified libraries were sequenced on an Illumina NovaSeq 6000 platform.

### scRNA-seq data analysis

Human single-cell data analysis was performed using SeekSoul Tools v1.2.1. Mouse single-cell data analysis was performed using the MobiVision v3.0 software. FASTP was used to process raw sequencing data^77^. After quality control, data were aligned to the reference genome to generate a cell–gene expression matrix. The data were further analyzed using Seurat v.4.0. Human cells with fewer than 200 or more than 5000 genes were removed; cells affected by mitochondrial genes were deleted using MAD-variance normalization^78^. Mouse cells with fewer than 500 genes or a proportion of mitochondrial genes over 0.25% were removed. After filtering low-quality cells, human samples were pooled, and the batch effect was eliminated using the CCA method. The UMI values were normalized using ScaleData/LogNormalize. Subsequently, a nonlinear dimension reduction was performed using RunPCA and RunUMAP. FindAllMarkers was used to identify DEGs and cluster-specific marker genes. The scaled expression data of these marker genes were used to create a bubble chart. Enrichment analysis of the DEGs was performed using GO (http://www.geneontology.org) and KEGG. GSVA was performed using the H, C2, and C5 genes from the MSigDB.

### Public scRNA-seq data analysis

The log2 (TPM) expression data from GSE131907 were obtained and used to construct Seurat (v5.1.0) objects with min.cells = 3 and min.features = 200. Quality control (QC) was performed by retaining cells with the following parameters: mitochondrial gene content ≤20%, nCount_RNA between 100–150,000, and nFeature_RNA between 200–10,000. For each batch, genes expressed in fewer than 0.1% of the cells were removed. Highly variable genes were selected using FindVariableFeatures (selection.method = “vst”, nfeatures = 4000), followed by data scaling (ScaleData), PCA (RunPCA, npcs = 50), UMAP (RunUMAP, dims = 1:20), neighbor graph construction (FindNeighbors, dims = 1:20), and clustering (FindClusters, resolution = 2.0). Cell annotations were assigned based on the original metadata, SingleR (v2.0.0), and canonical marker genes. Low-quality and doublet cells were excluded. Additionally, the GSE148071 dataset (pre-QC) was processed similarly. Seurat objects were created, normalized using NormalizeData, and variable features were selected (nfeatures = 600). Dimensionality reduction and clustering were performed using PCA (npcs = 50), UMAP (dims = 1:30), FindNeighbors, and FindClusters (resolution = 0.8). Cell types were defined with SingleR and known marker genes. Macrophages and fibroblasts with FN1 expression >0 were classified as FN1-positive (FN1^+^). Proportions of FN1^+/-^ macrophages/fibroblasts or the expression level of FN1 in macrophages/fibroblasts and immune cells (CD4^+^/ CD8^+^ T cells) were calculated per sample. Spearman correlation analysis was performed on both these cell types using the corr.test function from the psych package, and visualized using corrplot. Additionally, mean expression levels of immunosuppressive, pro-inflammatory, and lipid metabolism-associated genes were compared between the FN1^+^ and FN1^-^ macrophages. These were ultimately visualized using the ComplexHeatmap package.

### Bulk RNA-seq

Total RNA was extracted using the TRIzol reagent (TaKARa, 9109). RNA library preparation was carried out using the BGI Optimal series dual-module mRNA library construction kit (BGI), following the manufacturer’s protocols. After assessing the library quality, RNA-seq was conducted by the Wuhan BGI Technology Service Company using the MGISEQ-200 platform. Transcript analysis was performed using Dr. Tom software (version 2.0; Biosys.bgi.com).

### Mass cytometry

Tumor tissues were dissociated and digested into single-cell suspensions as described above, which were subsequently labeled with 194Pt for 5 minutes to distinguish live and dead cells. After Fc blocking (PLT Tech, 02.02.115), the cells were stained with a metal-labeled surface marker antibody cocktail containing 28 antibodies (CD45-89Y, CD3ε−115Ιn, Gr-1(Ly-6G/Ly-6C)-141Pr, CD11c-142Nd, CD69-145Nd, CD279(PD-1)-146Nd, CD161(NK-1.1)-147Sm, Ly-6C-148Nd, CXC3CR1-149Sm, CD44-150Nd, CD62L-154Sm, CD80-155Gd, CD25(IL-2Rα)-157Gd, CD19-158Gd, F4/80-159Tb, CD115(CSF-1R)-160Gd, CD274(PD-L1)-161Dy, CD103-164Dy, CD64(FcγRI)-165Ho, Ly-6G-166Er, CD47-169Tm, CD86-170Er, CD192(CCR2)-174Yb, TCR β chain-175Lu, MHC II(I-A/I-E)-176Yb, CD4-197Au, CD8a-198Pt, CD11b-209Bi) at room temperature for 30 min. After washing with cell-staining buffer (Standard BioTools, 201068) and DNA intercalator-Ir (Standard BioTools, 201192B), overnight staining was performed in Fix and Perm Buffer (Standard BioTools, 201067). Intracellular marker staining was performed using an intracellular or nuclear antibody mix containing 13 antibodies (Ki-67-139La, Granzyme B Recombinant-143Nd, TNF-144Nd, IL-1β-151Eu, IFN-γ-152Sm, IL-4-153Eu, CD206(MMR)-162Dy, IL-6-163Dy, Arginase1-167Er, FOXP3-168Er, Hexokinase II-171Yb, Perforin-172Yb, and IL-10-173Yb) for 30 min. Sample barcoding was performed for 30 min using unique barcode isotope combinations. The cells were resuspended in DI water and analyzed using a CyTOF machine (Helios, Standard BioTools). The antibody information is provided in Supplementary Data 1.

### Mass cytometry data analysis

Raw data were debarcoded using a doublet-filtering scheme^79^, and normalized through the bead normalization method^80^. Data were gated manually using the bead normalization method. The X-shift clustering algorithm^81^ was applied to partition cells into distinct phenotypes based on marker expression levels. The cell type in each cluster was annotated according to its marker expression patterns. The dimensionality reduction algorithm t-SNE was used to visualize high-dimensional data in two dimensions and to display the distribution of each cluster and marker expression.

### Sample preparation and force indentation acquisition by AFM

Tissue and ECM stiffness were tested following the methodology detailed in reference^82^. For AFM measurements, OCT-embedded frozen tumor tissues were sectioned at a thickness of 20 μm. The sections were then transferred to AFM-compatible glass bottom dishes coated with CellTak (Corning, 354242) and immersed in PBS containing a proteinase inhibitor. For ECM measurements, macrophages were resuspended at 2 × 10^6^ cells/mL in a mixture prepared on ice with a volume ratio of 1:1:8 of 3.7% Sodium Bicarbonate Solution (pH 9.5), 1 M HEPES (Sigma, H0887), and Collagen I solution (Corning, CLS354249, concentration range 8–11 mg/mL). The pH of the mixture was confirmed to be between 7.0 and 7.4 using pH indicator paper. The cell suspension was supplemented with IL-4 (10 ng/mL) and IL-13 (10 ng/mL). A 10 μL aliquot of this mixture was seeded onto AFM-compatible glass-bottom dishes. The dishes were inverted and incubated at room temperature to allow solidification, followed by the addition of culture medium. After 48 h of incubation, cells were fixed with paraformaldehyde. The following day, the samples were quantified using AFM. The cantilevers of the AFM probes (NP-O, Bruker) were glued to a polystyrene bead (diameter: 8 μm, Beijing Dk Nano Technology Co., Ltd.) using an epoxy adhesive. The cantilevers were tapped onto the tumor sections or the flat top surface of the collagen hydrogel, and three 10 μm × 10 μm stiffness maps (20 × 20 raster series) were constructed for each sample. The indentations were performed at a loading force of 2 nN and a constant speed of 2 μm/s (NanoWizard IV, JPK). Young’s modulus was determined by fitting a force–distance curve to the Hertz–Sneddon model.

### CD8^+^ T-cell activation

Naive CD8^+^ T cells were purified from the spleen of mice using the MojoSort Mouse CD8 T Cell Isolation Kit (Biolegend, 480035). CD8^+^ T cells were activated with anti-CD3 (5 μg/mL; Selleck, A2104) and anti-CD28 (5 μg/mL, Selleck, A2108) for 24 h using 10% FBS RPMI-1640 medium containing 100 IU/mL recombinant IL-2 (PeproTech, 212-12-20UG).

### Cell-Tracker staining

CD8^+^ T cells were fluorescently labeled using freshly prepared dyes from CellTracker CM-DiI (553/570 nm; Invitrogen, A66432). To prepare the dye, 36 μL of DMSO was added to a CellTracker stock vial. The cells in the suspension were centrifuged and resuspended in pre-warmed PBS (PBS/2% FBS) at a maximum concentration of 1 × 10^6^ cells/mL. Subsequently, 5 μL of CellTracker dye per mL of cell suspension was added, and the cells were incubated for 30 min at 37 °C. Following incubation, the cells were centrifuged, the supernatant was removed, and the cells were washed 2–3 times with an appropriate amount of pre-warmed buffer solution. The labeled cells were resuspended in the desired volume.

Macrophages were fluorescently labeled using freshly prepared dyes from eFluor 670 (633/670 nm; Invitrogen, 65-0840-85). The eFluor working solution was prepared by adding 1 μL of eFluor 670 to 1 mL of PBS and preheating in a 37 °C water bath. The macrophages were centrifuged at 300 × g for 5 min, resuspended in the eFluor 670 working solution, incubated in a 37 °C incubator for 5 min, and incubated for 15 min at 4 °C. Next, the cells were centrifuged at 300 × g for 5 min, the eFluor 670 working solution was aspirated, and the cells were washed once with macrophage medium (RPMI-1640 supplemented with penicillin/streptomycin and 10% heat-inactivated FBS).

### In vitro 3D co-culture chip

Tumor cells and macrophages were co-seeded or separately seeded into the OC-Plex^83^ using a high concentration of collagen type I rat tail (Corning, CLS354249) as a hydrogel matrix for 3D cell culture. Briefly, tumor cells (2 × 10^6^ cells/mL) and macrophages (2 × 10^6^ cells/mL) were co-suspended in 100% Collagen I, incubated on ice, and supplemented with IL-4 (10 ng/mL), IL-13 (10 ng/mL), or CXCL9 (50 ng/mL). For co-seeding, 1.4 μL of the cell–gel mixture was seeded into the gel channel of the organ chip. The device was placed in a Petri dish containing 10 mL of molecular-grade biowaste and 50 μL of PBS to prevent gel deformation, followed by incubation for 60 min at 37 °C with 5% CO_2_ to allow polymerization. After incubation, 60 μL of macrophage medium was added to the perfusion channel of each OC-Plex chip. The OC-Plex devices were placed in a humidified incubator, and the medium was refreshed every 2–3 days. Activated CD8^+^ T cells were added to the perfusion channels.

Imaging was performed using a Nikon turntable confocal microscope (CSUW1, Tokyo, Japan) with a 10× air objective. Three 5% overlapping fields of view and 15 Z-slices (step size, 14 μm) were acquired per chip to cover the entire ECM channel height. LLC cells were acquired using the GFP channel (excitation wavelength 488/40 nm, emission wavelength 507/40 nm), macrophages using the CY5 channel (excitation wavelength 647/40 nm, emission wavelength 660/40 nm), and T cells using the CY3 channel (excitation wavelength 550/40 nm, emission wavelength 570/40 nm). The fluorescence images acquired in each channel were processed using software for automatic stitching and 2D projection and subsequently used for corresponding image analysis.

### Intravital microscopy

The mice were injected with CellTracker CM-Dil-labeled CD8^+^ T cells via the tail vein. After 24 and 48 h, the mice were anesthetized, and tumors were imaged by intravital microscopy (IVIM Technology, IVM-CMS3)^84^.

### Cell surface and intracellular cytokine staining for flow cytometry

Cells were stained with fluorescence-labeled antibodies (e.g., CD14, CD163, CD86, CD11b, F4/80, CD274, CD45, CD8, PD-1) and the Zombie Aqua Fixable Viability Kit (BioLegend, 423101) for 20 min at 4 °C in the dark and then measured with a DxFLEX instrument (Beckman Coulter). Following the fixation/permeabilization steps using an intracellular staining perm wash buffer (BioLegend, 421002), intracellular staining was performed using fluorescence-labeled antibodies (e.g., CD206, IL-6, TNF, IL-1β, IFN-γ, perforin, granzyme B) or the validated primary antibody (Anti-Fibronectin antibody, Abcam, ab32419) and fluorescent secondary antibody (Alexa Fluor 488, Abcam, ab150077), and Rabbit monoclonal IgG (Abcam, ab172730) was used as the isotype control. Data were analyzed using the FlowJo software v10.8.1. Imaging flow cytometry analysis was performed by ImageStream Mk II (Amnis) according to the manufacturer’s instructions, and the data were analyzed using IDEAS 3.0.

### FN1-knockdown in THP-1 cells

Plasmids of FN1 shRNA were constructed by Shanghai Genechem Co., Ltd. THP-1 cells were infected with lentivirus harboring FN1 shRNA (shNC: 5′-TTCTCCGAACGTGTCACGT-3′, shFN1-1:5′-GTTGTTATGACAATGGAAA-3′, shFN1-2:5′-CAGCACAACTTCGAATTAT-3′) for 48 h and purified by puromycin (MedChemExpress, HY-B1743A, 2 μg/mL). Transfection was performed using jetPRIME Versatile DNA/siRNA (Polyplus, 114-15) according to the manufacturer’s protocol.

### Immunofluorescence staining

Live cells were incubated with Lysosome tracker-Red (Beyotime Biotechnology, C1046) for 1 h at 37 °C. Cell slides were fixed in 4% paraformaldehyde for 30 min, blocked with 3% goat serum and 0.1% Triton X-100 (Solarbio, T8200) for 30 min at 37 °C, incubated with a primary antibody against PFKP (Abcam, ab119796, 1:100)/LC3B (CST, 43566, 1:10000) or RAC1 (Absin, abs100465, 1:200)/mTOR (CST, 2983, 1:200) overnight at 4 °C, and then with a secondary antibody (Jackson, Alexa Fluor 488-AffiniPure Donkey Anti-Mouse IgG, 1:1000; Alexa Fluor 594-AffiniPure Donkey Anti-Rabbit IgG, 1:1000) for 1 h. Phalloidin-iFluor 647 Conjugate (AAT Bioquest, 1:1000) was used for F-actin staining according to the manufacturer’s protocol. After washing, the cells were sealed with an antifade mounting medium containing DAPI (Beyotime Biotechnology, P0131). Confocal images were acquired using a Zeiss LSM880 microscope at 63× or 100× objective magnification and a Leica STELLARIS 5 microscope at 63× objective magnification. Median filtering was applied to confocal images for noise reduction using ImageJ 1.54p. Colocalization analysis was also performed using ImageJ software. Subsequently, Pearson’s correlation coefficient (R value) was calculated to assess the extent of colocalization across a minimum of three images.

### Multiplex immunohistochemistry staining

Multiplex immunohistochemistry staining was performed using the PANO 7-plex IHC kit (Panovue, 10217100100)^85^. Paraffin-embedded tissue sections of NSCLC and mouse tumor samples were dehydrated and de-paraffinized. After antigen retrieval, the slides were blocked and incubated with primary antibodies (CD4, Cell Signaling Technology, 25229, 1:100; CD8, Cell Signaling Technology, 98941, 1:200; CD20, Abcam, ab64088 1:100; NKR-P1C, Abcam, ab289542, 1:100; F4/80, Cell Signaling Technology, 70076, 1:200; CXCL9, Invitrogen, 701117, 1:100; CXCL10, Invitrogen, 701225, 1:300; PFKP, Abcam, ab119796, 1:200; p62, Abcam, ab109012, 1:200), horseradish peroxidase-conjugated secondary antibodies, and tyramide signal amplification (TSA) sequentially. After each round of TSA, the slides were microwave-treated. Cell nuclei were stained with DAPI (Sigma-Aldrich, D9542-1MG) after all serial staining steps and mounted. Staining was performed using a VS200 MTL microscope (Olympus, Tokyo, Japan). The number of positive cells was counted using QuPath v0.3.0.

### Seahorse assay

The extracellular acidification rate (ECAR) of macrophages was measured according to the manufacturer’s protocol. The cells were seeded in an XF96 microplate (Agilent Technologies) at 5 × 10^4^ cells/well and allowed to adhere overnight. Cells were washed/incubated with Seahorse XF RPMI Medium (Agilent) supplemented with 2 mM l-glutamine in a non-CO_2_ incubator at 37 °C for 1 h. ECAR was assessed using an XF96 Extracellular Flux Analyzer (Agilent). Glucose (10 mM), oligomycin (1 μM), and 2-DG (100 mM) were sequentially injected into the plates (Seahorse XF Glycolysis Stress Test Kit, Agilent, 103020-100). For OCR, cells were washed/incubated with Seahorse XF RPMI Medium (Agilent) supplemented with 1 mM pyruvate, 2 mM l-glutamine, and 10 mM glucose in a non-CO_2_ incubator at 37 °C for 1 h. Oligomycin (1.5 μM), FCCP (0.5 μM), antimycin A, and rotenone (0.5 μM) were sequentially injected into the plates (Seahorse XF Cell Mito Stress Test kit, Agilent, 103015-100). The data were analyzed using Wave v2.6. software.

### Immunoblotting and immunoprecipitation

Cells were lysed using radioimmunoprecipitation assay (RIPA) buffer supplemented with a protease inhibitor cocktail (Sigma-Aldrich, P8340) and phosphatase inhibitor cocktail 2 (Sigma-Aldrich, P5726). The lysate was centrifuged at 12,000 × *g* for 10 min at 4 °C, and the supernatant was collected and boiled as the final lysate. Subsequently, the protein was separated via 7.5%, 10%, or 12.5% SDS-PAGE, and then transferred to NC membranes. The same were blocked with 5% BSA for 3 h and incubated with primary antibodies overnight at 4 °C, followed by incubation with horseradish peroxidase-conjugated secondary antibodies for 1 h. Proteins were detected via the ECL detection system. The antibody information is provided in Supplementary Data 1.

For immunoprecipitation, cells were lysed according to the manufacturer’s protocol (Absin, abs955). The lysate was centrifuged at 12,000 × *g* for 10 min at 4 °C. Thereafter, mTOR antibody (CST, 2983, 1:100) and isotype control (CST, 2279, 1:100) were added and incubated overnight at 4 °C. Protein A/G was added subsequently, and the mixture was incubated for 3 h. The resultant immune complexes were pelleted by centrifugation and washed three times with wash buffer. The pellet was resuspended in SDS sample buffer, heated at 95–100 °C for 5 min, then briefly centrifuged, followed by SDS-PAGE and western blotting.

### ELISA

The levels of CXCL9, CXCL10, and FN1 in the macrophage culture medium were detected using ELISA kits according to the manufacturer’s instructions (Multi Sciences, EK1143, EK168, and Reddot Biotech, RDR-FN-Hu).

### RT-qPCR

Total RNA from macrophages was isolated using TRIzol reagent (TaKARa, 9109). Total RNA (1 μg) was reverse transcribed to cDNA following the manufacturer’s protocol (Vazyme, R333-01). PCR (RT-qPCR) was performed using real-time PCR SYBR (Vazyme, Q712-02) on a CFX96 Real-Time System (Bio-Rad). The primer sequences for specific genes are shown in Supplementary Table 2.

### Statistics

Comparisons between two groups were performed using unpaired or paired two-tailed Student’s *t*-tests. One-way analysis of variance (ANOVA) was used to compare the means of three or more independent groups. Tukey’s multiple comparisons test was used to compare all treatment groups when the overall *p*-value was <0.05. Two-way ANOVA was used when the experiment had two variables. Sidak’s multiple comparison test was used to compare all treatment groups when the overall *p*-value was <0.05. Survival analysis was performed using the log-rank test for comparison. Statistical analyses were performed using GraphPad Prism 8.0.1 and GraphPad Prism 7.0. All quantitative data are presented as the mean ± standard deviation (SD) or mean ± standard error of the mean (SEM), as indicated in the figure legends. The statistical details of the experiments, including the statistical test used, the value of n, and what n represents, can be found in the figure legends. All *p*-values below 0.05 were considered significant.

### Reporting summary

Further information on research design is available in the Nature Portfolio Reporting Summary linked to this article.

## Supplementary information

Supplementary Information

Description of Additional Supplementary Files

Supplementary Data 1

Reporting Summary

Transparent Peer Review file

## Source data

Source Data

## Data Availability

Single-cell RNA-seq and bulk RNA sequencing data have been deposited in the Genome Sequence Archive at the National Genomics Data Center, China National Center for Bioinformation/Beijing Institute of Genomics, Chinese Academy of Sciences. Human data are accessible through GSA-human: HRA006716, HRA009508 and HRA009520. According to Chinese regulatory requirements, all human sequencing data must be subject to controlled access. Applicants can follow the steps in the document (https://ngdc.cncb.ac.cn/gsa-human/document/GSA-Human_Request_Guide_for_Users_us.pdf) to apply for access. Mouse data are accessible through GSA: CRA020886. The western blotting images and microscopic data reported in this paper have been deposited in Figshare (10.6084/m9.figshare.28211138). All data are included in the Supplementary Information or available from the authors, as are unique reagents used in this Article. The raw numbers for charts and graphs are available in the Source Data file whenever possible. Source data are provided with this paper.
