# Supplementary material for: Macrophage-derived fibronectin suppresses antitumor immunity via tissue stiffening and immunosuppressive cell induction in cancer mouse models
Source: Nat Commun. 2026 May 22;17:7296. doi: 10.1038/s41467-026-73287-7 (PMC13402297; doi:10.1038/s41467-026-73287-7)
Supplement: Supplementary file 1 — Supplementary Information [file 41467_2026_73287_MOESM1_ESM.pdf]

Supplementary Information

Macrophages-derived fibronectin suppresses anti-tumor immunity via tissue stiffening and immunosuppression induction

First authors: Aitian Li, Ying Wang, Haiqing Bai, Xin Xie

Corresponding authors: Li Yang, Yi Zhang

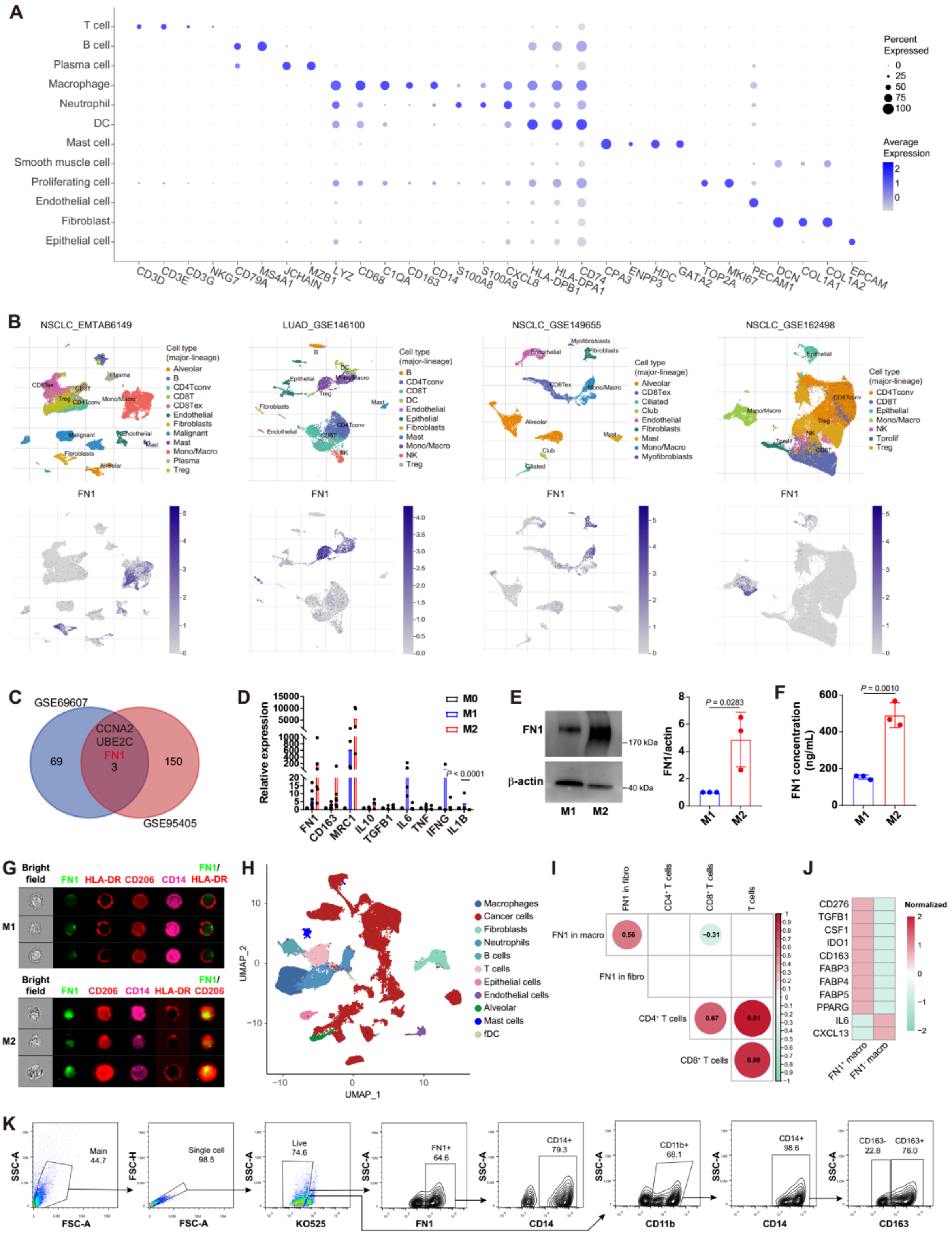

Supplementary Figure 1. FN1⁺ macrophages exert an immunosuppressive phenotype in NSCLC. (A) Expression levels of marker genes among different cell types. (B) UMAP plots of cells from public scRNA-seq data in TISCH2. UMAP plots were overlaid with FN1. (C) Common genes in two bulk RNA-seq datasets. (D) qRT-PCR analysis of *FN1, CD163, MRC1, IL10, TGFB1, IL6, TNF, IFNG*, and *IL1B* in M0-like, M1-like, and M2-like MDMs. $n \geq 3$ biologically independent samples. (E) Representative western blotting image and relative analysis of FN1 in M1-like and M2-like MDMs. n = 3 biologically independent samples. (F)

ELISA analysis of FN1 secretion in the culture medium of M1-like and M2-like MDMs. *n* = 3 biologically independent samples. (G) Imaging flow cytometry analysis of FN1, CD14, CD163, CD206, and HLA-DR in M1-like and M2-like MDMs. Experiment was repeated three times independently with similar results. (H) UMAP plots of cells from public scRNA-seq data GSE148071. (I) Spearman correlation between the expression of FN1 in macrophages or fibroblasts and the proportions of immune cells ($CD4^+$ and $CD8^+$ T cells) from public scRNA-seq data GSE148071. (J) Heatmap of immunosuppressive, lipid metabolism-related, and proinflammatory genes in both $FN1^+$ and $FN1^-$ macrophages from public scRNA-seq data GSE148071. (K) Cell gate protocol for tumor-infiltrating $CD14^+$ macrophages in Fig. 1F, 1G. Data are displayed as the mean (D) and mean ± SD (E, F), two-tailed unpaired *t*-test (D, E, F). scRNA-seq, single-cell RNA sequencing; qRT-PCR, quantitative reverse transcription polymerase chain reaction; MRC1, mannose receptor C-type 1; IL, interleukin; TGFB1, transforming growth factor beta 1; TNF, tumor necrosis factor; IFNG, interferon gamma; MDM, monocyte-derived macrophage.

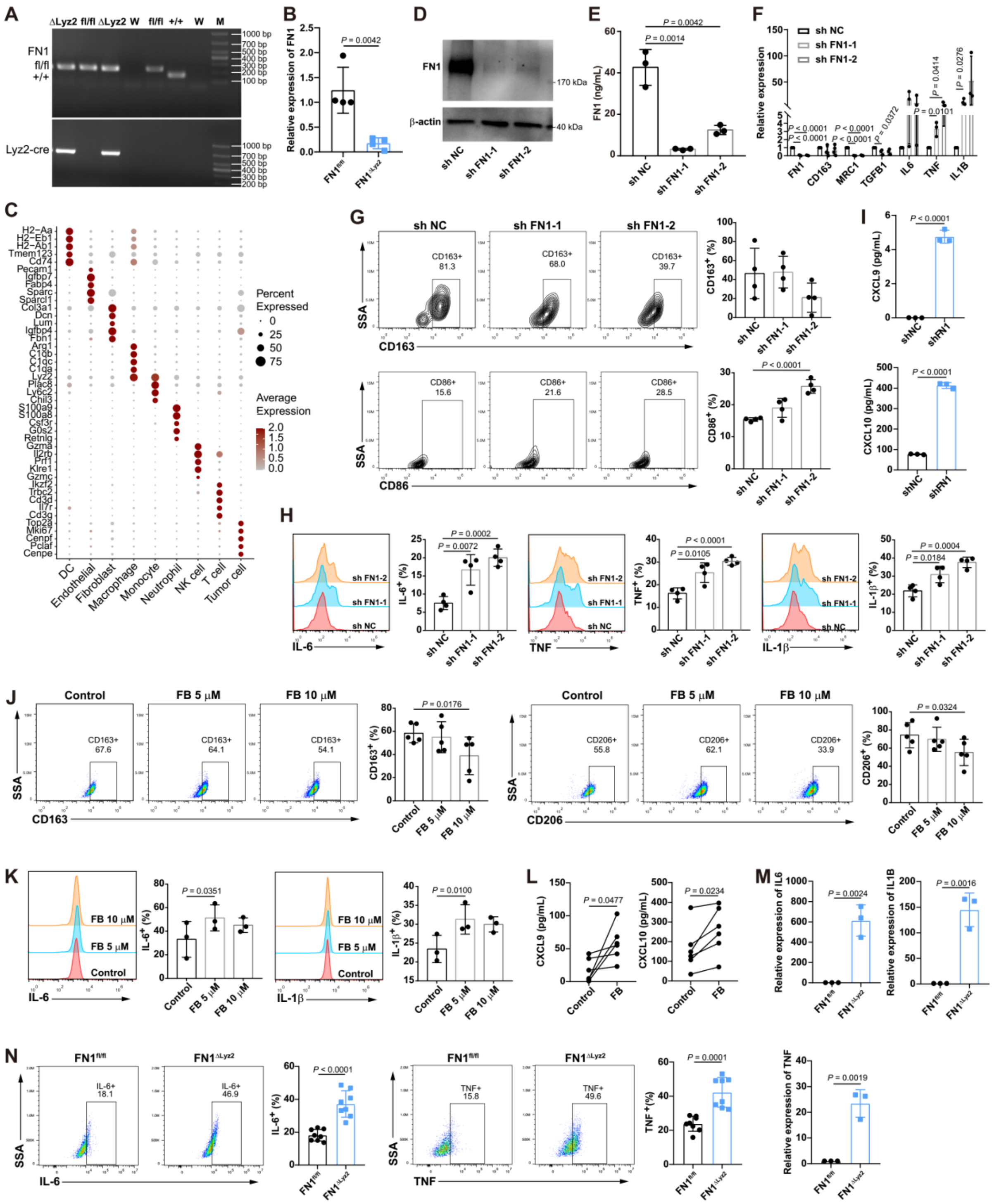

Supplementary Figure 2. FN1 deficiency reprograms immunosuppressive macrophages into pro-inflammatory phenotype. (A) Genotyping of wildtype (+/+), homozygous knock-in (fl/fl), and myeloid-specific depletion of FN1 (ΔLyz2) mice. (B) qRT-PCR analysis of FN1 expression in BMDMs from $FN1^{fl/fl}$ and $FN1^{\Delta Lyz2}$ mice. $n = 4$ biologically independent samples. (C) Expression levels of marker genes among different cell types. Representative western blotting image (D) and ELISA (E) analyses of FN1 expression in shNC and shFN1 THP-1-induced macrophages. $n = 3$ biologically independent samples. (F) qRT-PCR analysis of *FN1, CD163, MRC1, TGFB1, IL6, TNF*, and *IL1B* in shNC and shFN1 THP-1-induced macrophages.

n ≥ 3 biologically independent samples. Representative contour plots/histogram and quantification of CD163, CD86 (G), IL-6, IL-1β, and TNF (H) expression in shNC and shFN1 THP-1-induced macrophages. *n* = 4 biologically independent samples. (I) ELISA analysis of CXLC9 and CXCL10 secretion in the culture medium of shNC/shFN1 THP-1-induced macrophages. *n* = 3 biologically independent samples. Representative dot plots/histogram and quantification of CD163, CD206 (J), IL-6, and IL-1β (K) expression in FB-treated MDMs. *n* = 3 or 5 biologically independent samples. (L) ELISA analysis of CXLC9 and CXCL10 secretion in the culture medium of FB-treated MDMs. *n* = 6 biologically independent samples. (M) qRT-PCR analysis of *IL6, IL1B* and *TNF* expression in $FN1^{fl/fl}$ and $FN1^{\Delta Lyz2}$ BMDMs. *n* = 3 biologically independent samples. (N) Representative dot plots and quantification of IL-6 and TNF expression in $FN1^{fl/fl}$ and $FN1^{\Delta Lyz2}$ BMDMs. *n* = 8 biologically independent samples. Data are displayed as the mean ± SD, two-tailed unpaired (B, E–I, M, N) or paired (J–L) *t*-test. qRT-PCR, quantitative reverse transcription polymerase chain reaction; BMDM, bone marrow-derived macrophage; IL, interleukin; TGFB1, transforming growth factor beta 1; TNF, tumor necrosis factor; FB, fibronectin blocker; MDM, monocyte-derived macrophage.

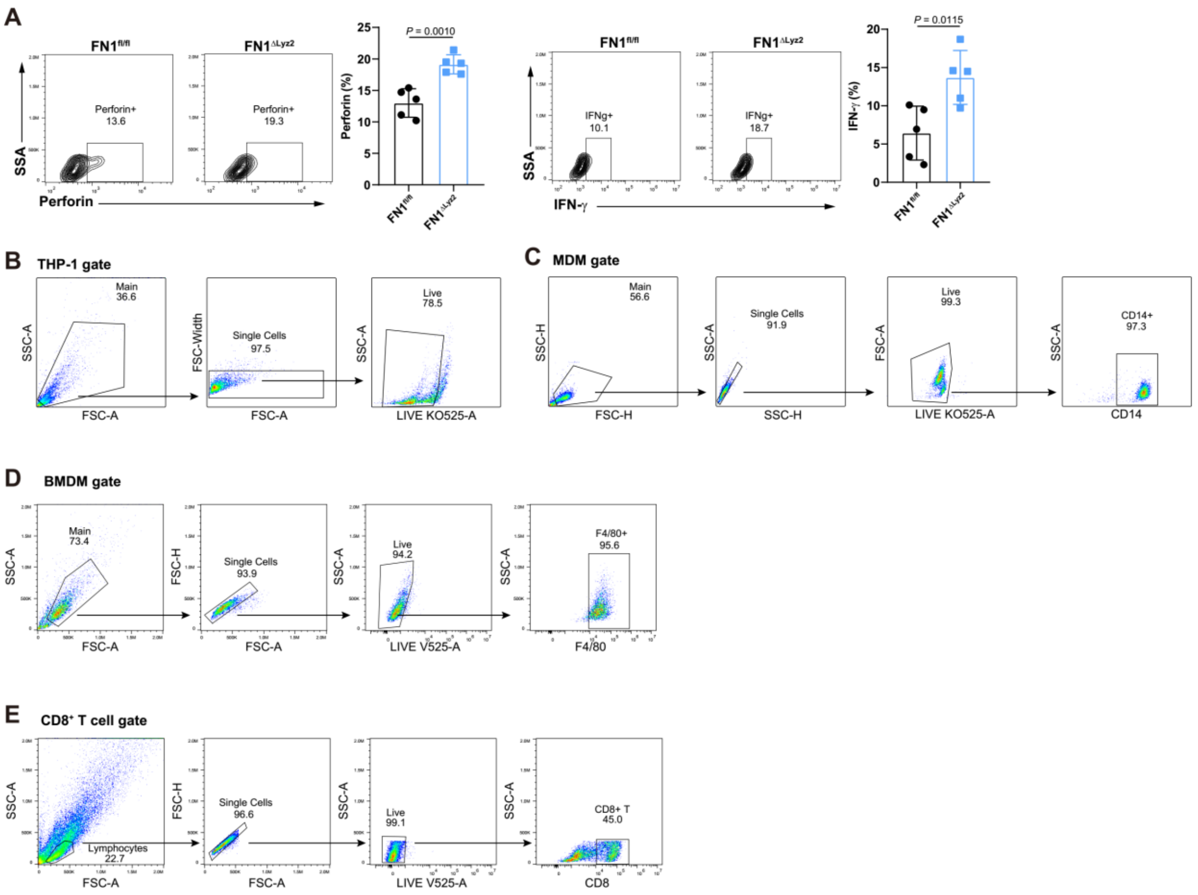

Supplementary Figure 3. FN1 deficiency promotes T-cell cytotoxicity. (A) Representative contour plots and quantification of perforin and IFN-γ expression in $CD8^+$ T cells co-cultured with $FN1^{fl/fl}$ and $FN1^{\Delta Lyz2}$ BMDMs in a ratio of 1:2 for 24h. n = 5 biologically independent samples. Cell gate protocol for THP-1-induced macrophages in Sup Fig. 2G, 2H (B), MDMs in Sup Fig. 2J, 2K (C), BMDMs in Sup Fig. 2N (D) and $CD8^+$ T-cells in Sup Fig. 3A (E). Data are displayed as the mean ± SD, two-tailed unpaired t-test. BMDM, bone marrow-derived macrophage; IFN-γ, interferon gamma; MDM, monocyte-derived macrophage.

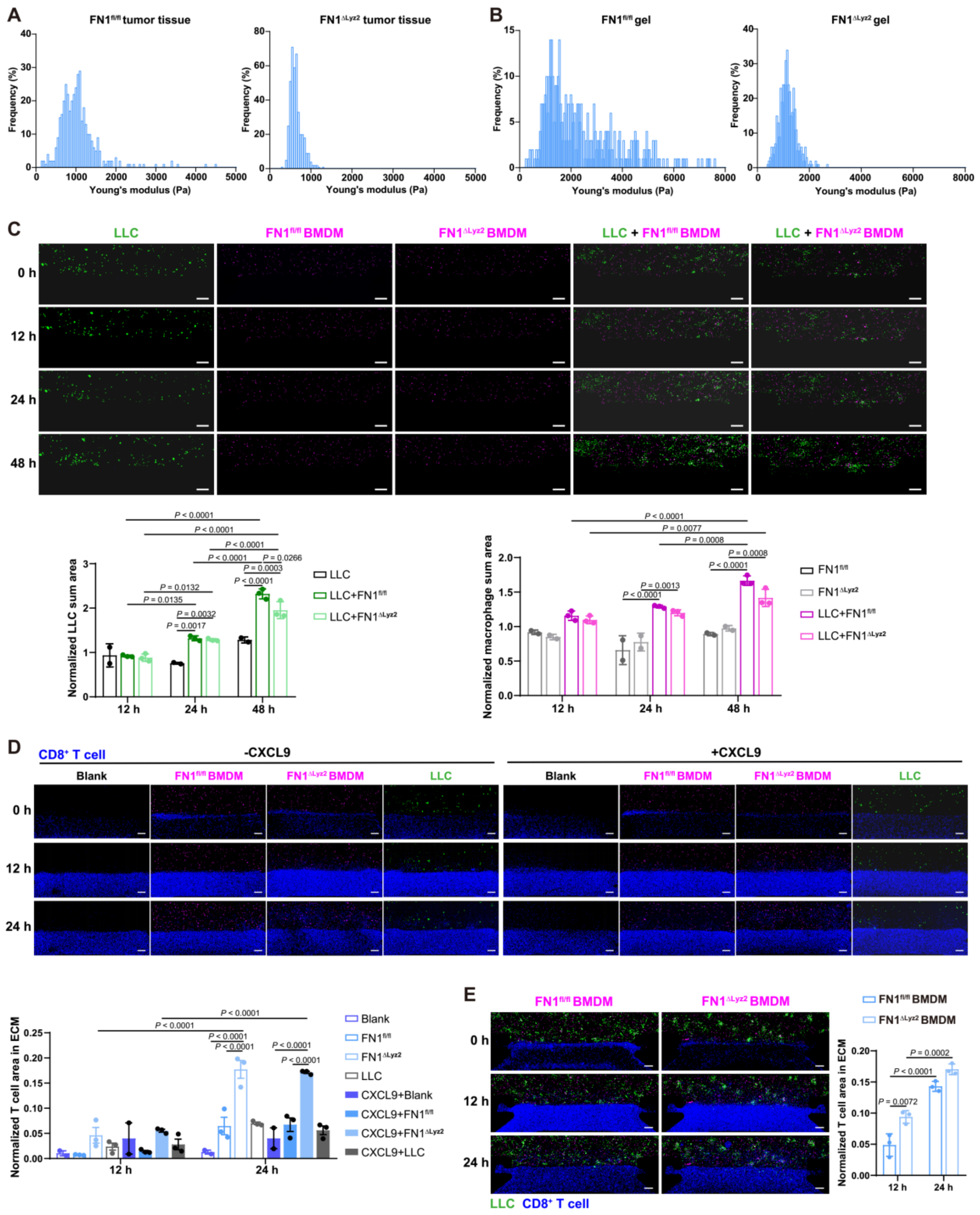

Supplementary Figure 4. FN1 deficiency promotes T-cell infiltration. Stiffness of tumor tissues (A) and BMDM-cultured matrix (B) from $FN1^{fl/fl}$ and $FN1^{\Delta Lyz2}$ mice. (C) Representative confocal images and analysis of cell proliferation of 3D-cultured LLC and BMDMs from $FN1^{fl/fl}$ and $FN1^{\Delta Lyz2}$ mice. *n* = 3 biologically independent samples. Scale bar, 100μm. (D) Representative confocal images and analysis of infiltrated $Dil^{+}CD8^{+}$ T-cells in hydrogel matrix of BMDMs from $FN1^{fl/fl}$ and $FN1^{\Delta Lyz2}$ mice or LLC with or without CXCL9 (50 ng/mL). *n* = 3 biologically independent samples. Scale bar, 100μm. (E) Representative confocal images and analysis of infiltrating $Dil^{+}CD8^{+}$ T-cells in LLC–$FN1^{fl/fl}$/$FN1^{\Delta Lyz2}$ BMDM

co-culture hydrogel matrix. *n* = 3 biologically independent samples. Scale bar, 100μm. Data are displayed as the mean ± SD, two-way ANOVA. BMDM, bone marrow-derived macrophage; LLC, lewis lung carcinom.

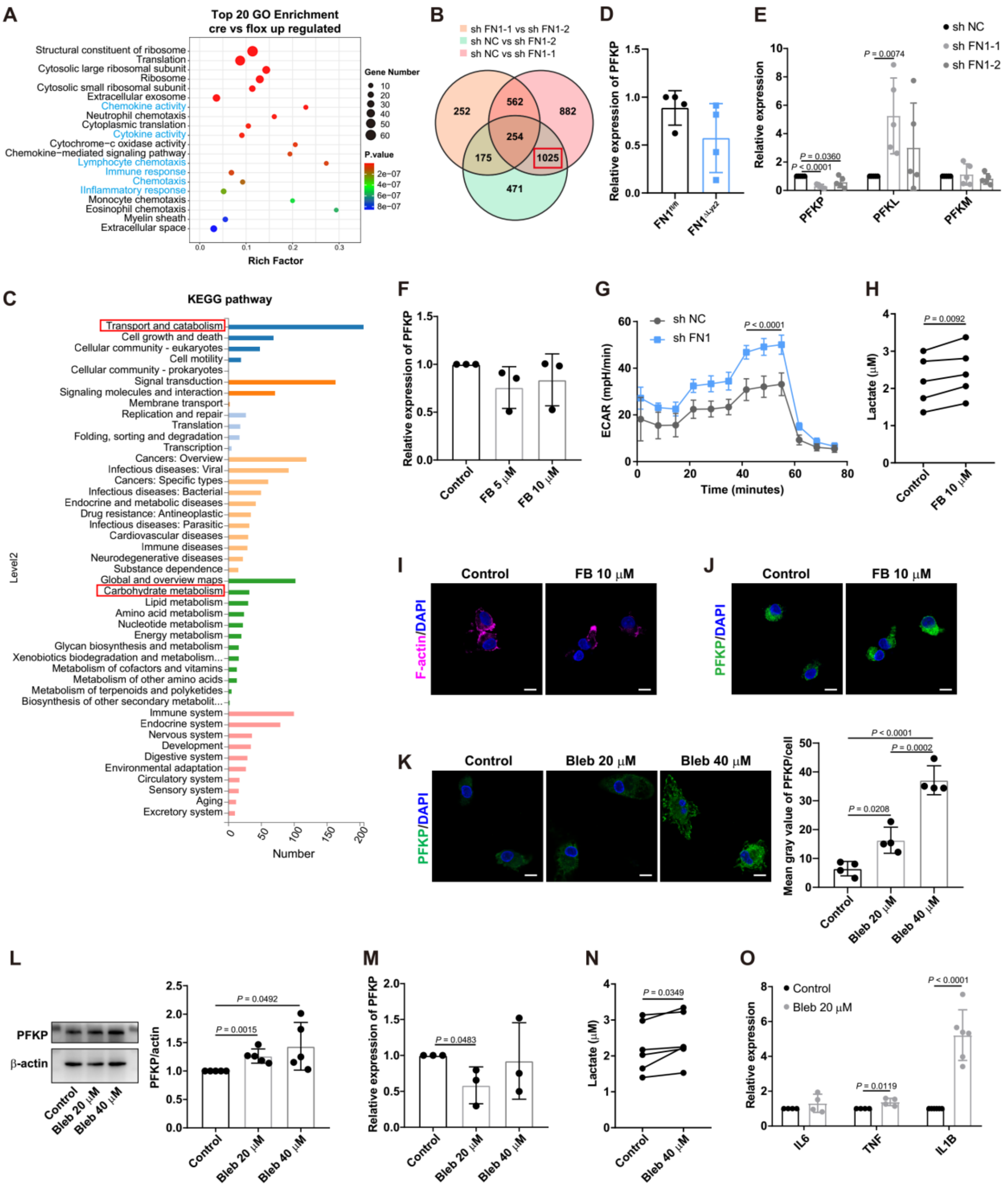

Supplementary Figure 5. FN1 inhibition increases glycolysis by decreasing macrophage actin polymerization *in vitro*. (A) GO enrichment analysis of differentially expressed genes (DEGs) in tumor-infiltrating macrophages from FN1$^{fl/fl}$ and FN1$^{\Delta Lyz2}$ mice. (B) DEGs in shNC/shFN1 THP-1-induced macrophages. (C) KEGG enrichment analysis of DEGs in shNC/shFN1 THP-1-induced macrophages. (D) qRT-PCR analysis of *PFKP* expression in BMDMs from FN1$^{fl/fl}$ and FN1$^{\Delta Lyz2}$ mice. *n* = 4 biologically independent samples. (E) qRT-PCR analysis of *PFKP, PFKL*, and *PFKM* expression in shNC/shFN1 THP-1-induced macrophages. *n* = 5 biologically independent samples. (F) qRT-PCR analysis of *PFKP*

expression in FB-treated MDMs. *n* = 3 biologically independent samples. (G) ECAR in shNC/shFN1 THP-1-induced macrophages. *n* = 5 biologically independent samples. (H) ELISA analysis of lactate levels in the culture medium of FB-treated MDMs. *n* = 5 biologically independent samples. Representative immunofluorescence image of F-actin (I) and PFKP (J) in FB-treated MDMs. Scale bar, 10 μm. Experiment was repeated three times independently with similar results. (K) Representative immunofluorescence image and quantification of PFKP expression in blebbistatin-treated MDMs. Scale bar, 10 μm. *n* = 4 biologically independent samples. (L) Representative western blotting image and relative analysis of PFKP expression in blebbistatin-treated MDMs. *n* = 5 biologically independent samples. (M) qRT-PCR analysis of PFKP expression in blebbistatin-treated MDMs. *n* = 3 biologically independent samples. (N) ELISA analysis of lactate levels in the culture medium of blebbistatin-treated MDMs. *n* = 6 biologically independent samples. (O) qRT-PCR analysis of *IL6, TNF,* and *IL1B* expression in blebbistatin-treated MDMs. $n \geq 4$ biologically independent samples. Data are displayed as the mean ± SD, hypergeometric test (A), two-tailed unpaired (D–F, L, M and O) or paired (H and N) *t*-test, one-way (K) or two-way ANOVA (G). GO, gene ontology; DEGs, differentially expressed genes; PFKP, phosphofructokinase, platelet; PFKL, phosphofructokinase liver; PFKM, phosphofructokinase muscle; FB, fibronectin blocker; BMDM, bone marrow-derived macrophage; MDM, monocyte-derived macrophage; ECAR, extracellular acidification rate; F-actin, actin filament; IL, interleukin; TNF, tumor necrosis factor.

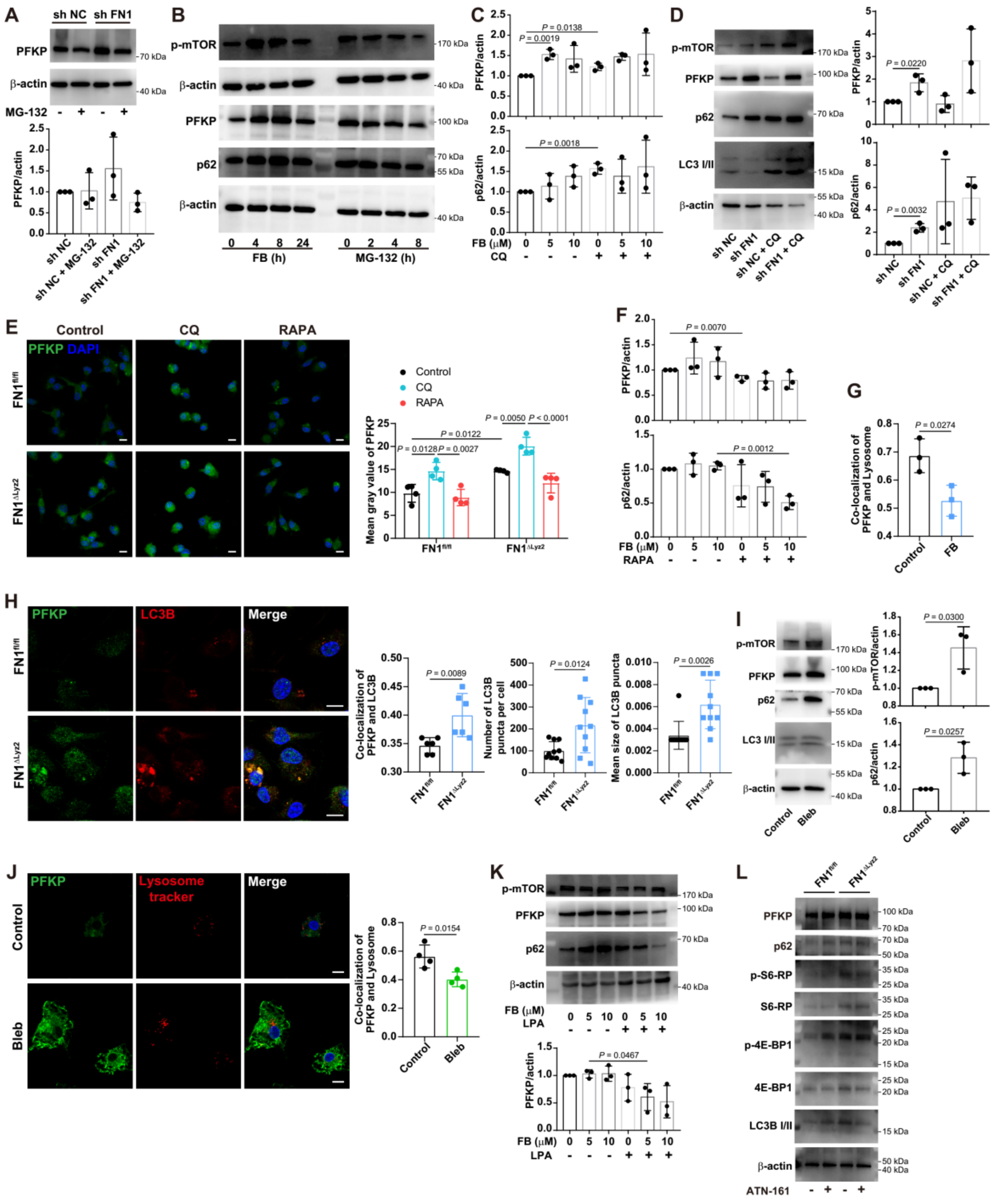

Supplementary Figure 6. Blockade of FN1-integrin interaction increases PFK by inhibiting autophagy through reduced F-actin. (A) Representative western blotting image and relative analysis of PFKP and β-actin expression in shNC and shFN1 THP-1-induced macrophages with MG-132 treatment. *n* = 3 biologically independent samples. (B) Representative western blotting image of p-mTOR, PFKP, p62, and β-actin expression in FB-treated MDMs for 0, 4, 8, and 24 h, as well as after MG-132 usage for 0, 2, 4, and 8 h. Experiment was repeated three times independently with similar results. (C) Quantification of PFKP and p62 expression in Fig. 5C. *n* = 3 biologically independent samples. (D) Representative western blotting image and relative analysis of p-mTOR, PFKP, p62, LC3I/II,

and β-actin expression in shNC and shFN1 THP-1-induced macrophages with CQ treatment. *n* = 3 biologically independent samples. (E) Representative immunofluorescence image and quantification of PFKP expression in $FN1^{fl/fl}$ and $FN1^{\Delta Lyz2}$ BMDMs treated with CQ or RAPA. Scale bar, 10 μm. *n* = 4 biologically independent samples. (F) Quantification of PFKP and p62 expression in Fig. 5D. *n* = 3 biologically independent samples. (G) Quantification of co-localization of PFKP and lysosomes within the confocal image in Fig. 5G. *n* = 3 biologically independent samples. (H) Representative immunofluorescence image and co-localization analysis of PFKP and LC3B, and quantification of the number and mean size of LC3B puncta in $FN1^{fl/fl}$ and $FN1^{\Delta Lyz2}$ BMDMs. Scale bar, 10 μm. *n* = 3 biologically independent samples. Each dot represents one field. (I) Representative western blotting image and relative analysis of PFKP, p-mTOR, p62, LC3I/II, and β-actin in blebbistatin-treated MDMs. *n* = 3 biologically independent samples. (J) Representative immunofluorescence image and co-localization analysis of PFKP and lysosomes in blebbistatin-treated MDMs. Scale bar, 10 μm. *n* = 4 biologically independent samples. (K) Representative western blotting image and relative analysis of p-mTOR, PFKP, p62 and β-actin in FB-treated MDMs with LPA usage. *n* = 3 biologically independent samples. (L) Representative western blotting image of PFKP, p62, p-S6-RP, S6-RP, p-4E-BP1, 4E-BP1, LC3BI/II, and β-actin expression in $FN1^{fl/fl}$ and $FN1^{\Delta Lyz2}$ BMDMs treated with ATN-161 (10μM). Experiment was repeated three times independently with similar results. Data are displayed as the mean ± SD, two-tailed unpaired *t*-test (A, C, D and F–K) or two-way ANOVA (E). PFKP, phosphofructokinase platelet; FB, fibronectin blocker; MDM, monocyte-derived macrophage; CQ, chloroquine; RAPA, rapamycin; LPA, lysophosphatidic acid; BMDM, bone marrow-derived macrophage.

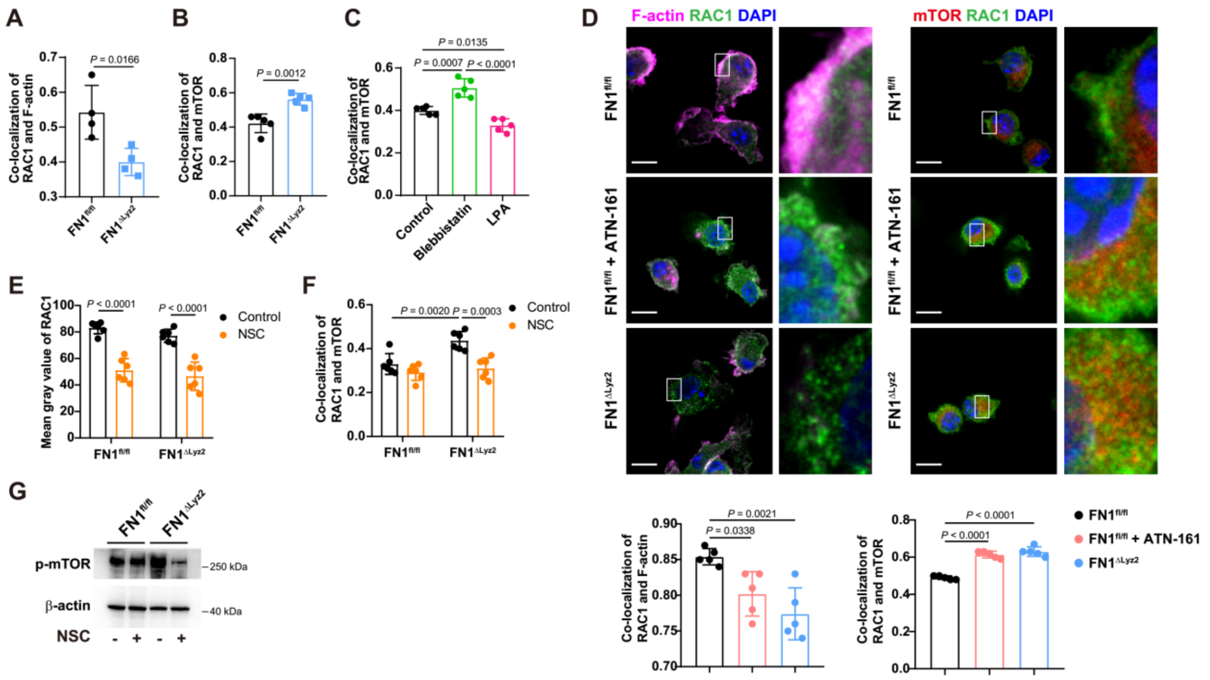

Supplementary Figure 7. Lack of FN1 increases PFK by activating the RAC1-mTOR axis and inhibiting autophagy through reduced F-actin. (A) Quantification of co-localizaition of RAC1 and F-actin within the confocal image in Fig. 5J. *n* = 4 biologically independent samples. Quantification of co-localization of RAC1 and mTOR within the confocal image in Fig. 5K (B) and Fig. 5M (C). *n* = 5 biologically independent samples. (D) Representative immunofluorescence image and quantification of co-localizaition of F-actin and RAC1, mTOR and RAC1 in FN1$^{fl/fl}$ and FN1$^{\Delta Lyz2}$ BMDMs treated with ATN-161 (10 μM). Scale bar, 10 μm. n = 5 biologically independent samples. Quantification of RAC1 expression (E), and the co-localization of RAC1 and mTOR (F) within the confocal image in Fig. 5N. *n* = 3 biologically independent samples. Each dot represents one field. (G) Representative western blotting image of p-mTOR and β-actin in FN1$^{fl/fl}$ and FN1$^{\Delta Lyz2}$ BMDMs treated with NSC-23766. Experiment was repeated three times independently with similar results. Data are displayed as the mean ± SD, two-tailed unpaired *t*-test (A and B), one-way (C and D) or two-way (E and F) ANOVA. F-actin, actin filament; BMDM, bone marrow-derived macrophage.

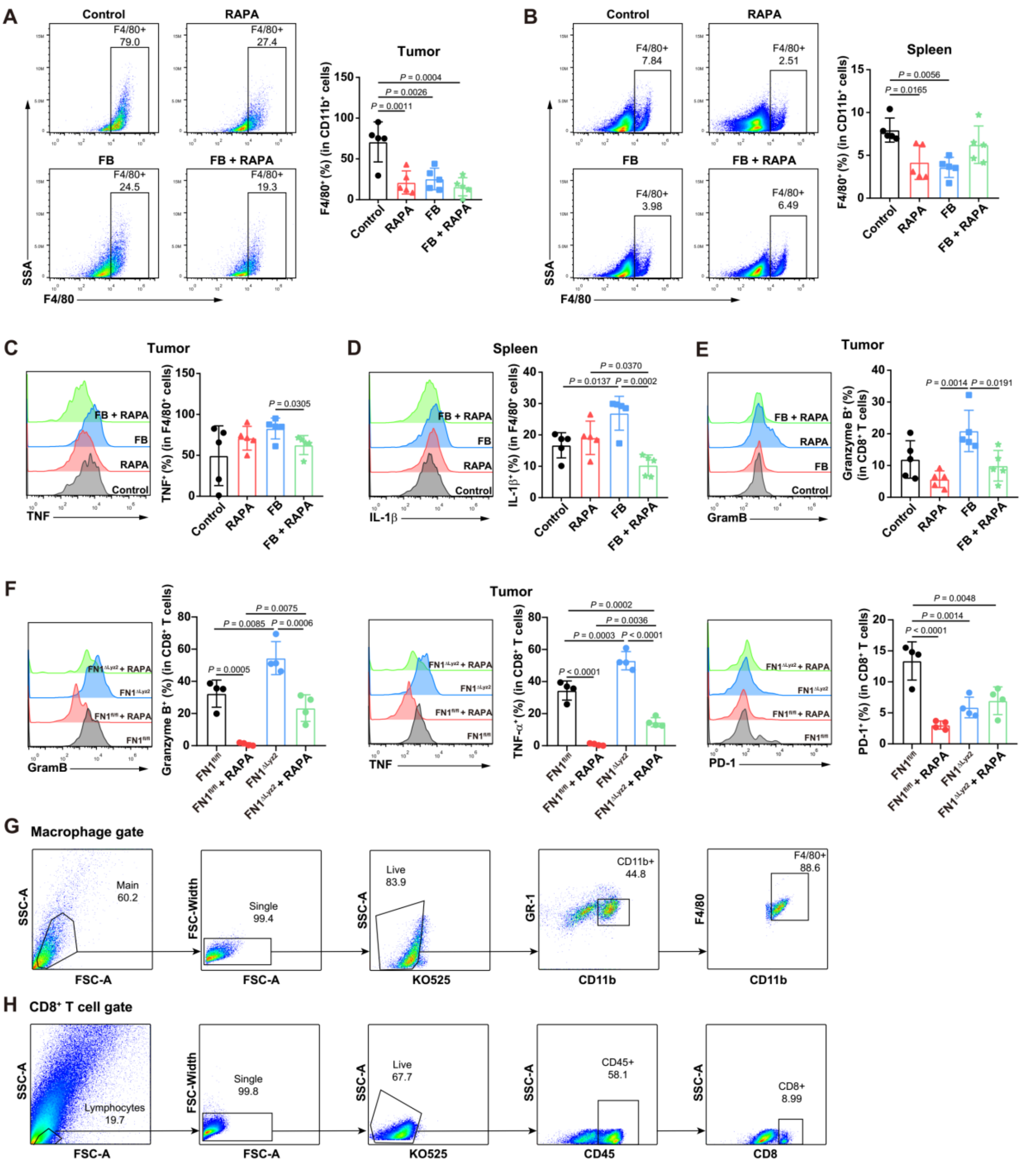

Supplementary Figure 8. Loss of FN1 attenuates autophagy and the immunosuppressive microenvironment *in vivo*. Representative dot plots and proportion of F4/80$^+$ macrophages among myeloid cells from tumors (A) and spleens (B) of C57BL/6 mice. n = 5 biologically independent samples. Histogram and quantification of TNF expression in F4/80$^+$ macrophages in tumor tissues (C), IL-1β expression in F4/80$^+$ macrophages in spleens (D), and granzyme B expression in CD8$^+$ T-cells in tumors (E). n = 5 biologically independent samples. (F) Histogram and quantification of granzyme B, TNF and PD-1 expression in CD8$^+$ T-cells in tumors from FN1$^{fl/fl}$ and FN1$^{\Delta Lyz2}$ mice with RAPA usage. n = 4 biologically independent samples. Cell gate protocol for F4/80$^+$ macrophages in Fig. 6D, 6I, Sup Fig. 8A–8D (G) and CD8$^+$ T-cells in Fig. 6E, 6J, Sup Fig. 8E, 8F (H). Data are displayed as the mean ± SD, two-tailed unpaired t-test (C), one-way ANOVA (A, B and D–F). IL, interleukin; TNF, tumor

necrosis factor; PD-1, programmed death-1, RAPA, rapamycin.

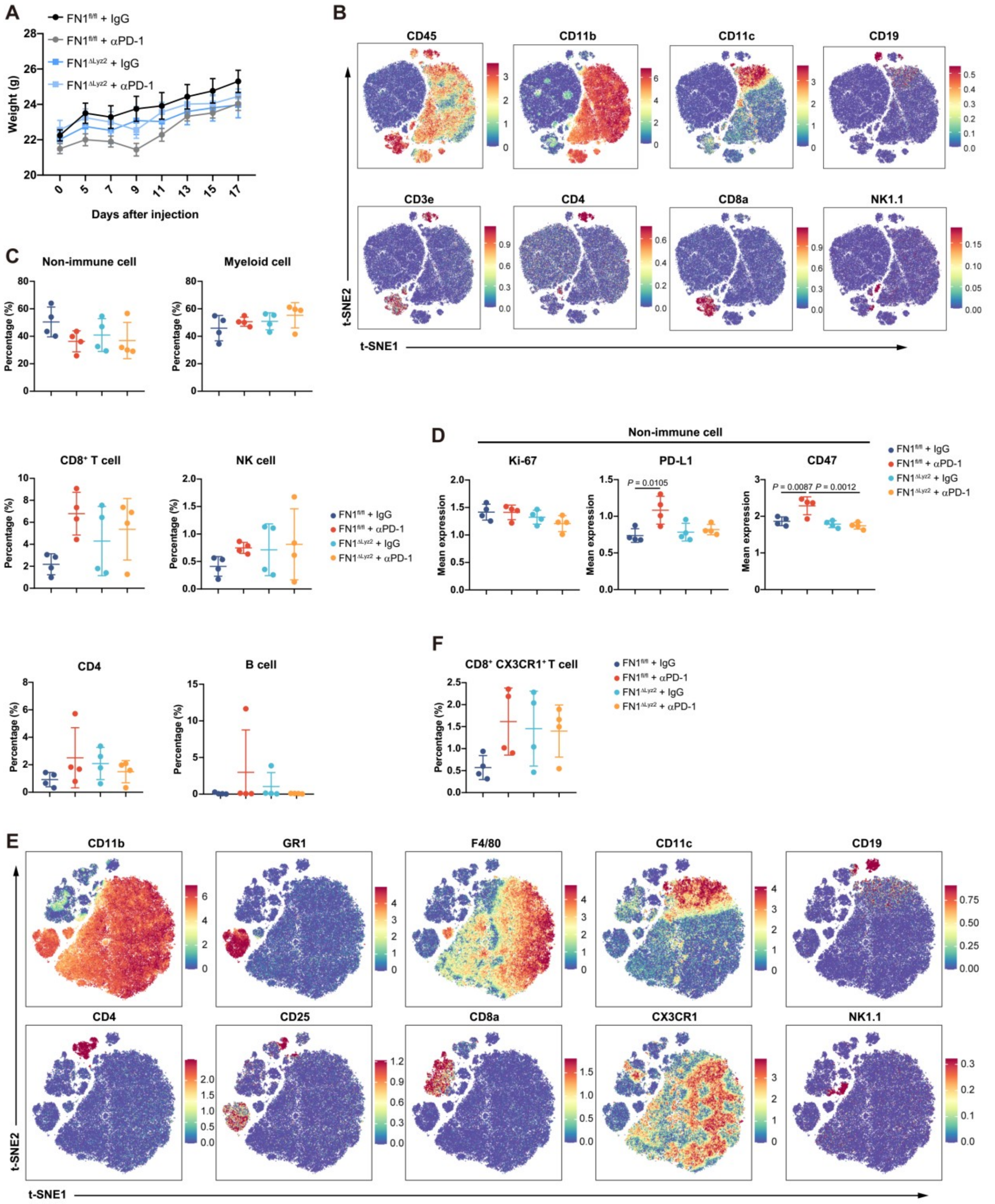

Supplementary Figure 9. Inhibition of FN1 improves the efficacy of anti-PD-1 therapy in NSCLC. (A) Mice weight measured every two days. n = 5 biologically independent samples for FN1$^{fl/fl}$ + anti-PD-1group, n = 6 biologically independent samples for other groups. (B) tSNE plots showing the overlay with marker genes. (C) Statistical analysis of the proportion of main cell clusters in tumors. n = 4 biologically independent samples. (D) Mass cytometry analysis of Ki-67, PD-L1, and CD47 expression in non-immune cells. n = 4 biologically independent samples. (E) tSNE plots showing the overlay with marker genes of subsets. (F)

Mass cytometry analysis of the proportion of $CD8^+CX3CR1^+$ T-cell clusters in tumors. $n = 4$ biologically independent samples. Data are displayed as the mean ± standard error of the mean (SEM) (A) and mean ± SD (C, D and F), one-way ANOVA. PD-L1, programmed death-ligand 1; PD-1, programmed death-1.

Stratification group		n
Age		42–78
Sex	Male	4
	Female	12
Diagnosis	Lung squamous cell carcinoma	4
	Lung adenocarcinoma	12
AJCC stage	I	11
	II	1
	III	4

Supplementary Table 1. Summary of clinical characteristics of patients with cancer. Samples were obtained from the First Affiliated Hospital of Zhengzhou University (n = 16). AJCC, American Joint Committee on Cancer.

Gene	Forward (5' to 3')	Reverse (5' to 3')	Species
FN1	AGGAAGCCGAGGTTTTAACTG	AGGACGCTCATAAGTGTCACC	Human
MRC1	GTGATGGGACCCCTGTAACG	CTGCCCAGTACCCATCCTTG	Human
IL10	TACCACCTCCCGAAAATGTCA	CCCAGTCTGAATGCTCATCTG	Human
PFKP	GTGGATTCGAGGCCTACCTG	GTTGGACACAGTAGCGGGAA	Human
PFKL	GCTGGGACAGACTGTTTCTTTC	GCCCTCATAGCCCTCGTAGA	Human
PFKM	GTCTCGCCAGTTAGTCAGGA	TGGTGGCTTTGGTATGAGCA	Human
CD163	TGTGGCCTGCATAGAGAGTG	TTCCCCAAAATGAGCAGAAC	Human
IL1B	ATGATGGCTTATTACAGTGGCAA	GTCGGAGATTCGTAGCTGGA	Human
TGFB1	GCCAGAGTGGTTATCTTTTGATG	AGTGTGTTATCCCTGCTGTCAC	Human
IFNG	TCGGTAACTGACTTGAATGTCCA	TCGCTTCCCTGTTTTAGCTGC	Human
TNF	GGCAGTCAGATCATCTTCTCGAA	GAAGGCCTAAGGTCCACTTGTGT	Human
IL6	TACCCCCAGGAGAAGATTCC	TTTTCTGCCAGTGCCTCTTT	Human
GAPDH	GCACCGTCAAGGCTGAGAAC	TGGTGAAGACGCCAGTGGA	Human
FN1	TCTCCTCCCATCCACTCA	TGCCTCTTGCTCTTCCC	Mouse
PFKP	CACCTATAAGCGTCTTGCC	GTGCCCCTACATTGATGAC	Mouse
IL6	AGCCCACCAAGAACGATAG	GGTTGTCACCAGCATCAGT	Mouse
IL1B	AGTTGACGGACCCCAAA	TCTTGTTGATGTGCTGCTG	Mouse
TNF	CGCTGAGGTCAATCTGC	GGCTGGGTAGAGAATGGA	Mouse
GAPDH	CCTCCTCCAATTCAACCCT	CACCGACCTTCACCATTTT	Mouse

Supplementary Table 2. Primer sequences used for qPCR. qPCR, quantitative polymerase chain reaction; IL, interleukin; GAPDH, glyceraldehyde 3-phosphate dehydrogenase
