# Supplementary material for: Macrophage-derived fibronectin suppresses antitumor immunity via tissue stiffening and immunosuppressive cell induction in cancer mouse models
Source: Nat Commun. 2026 May 22;17:7296. doi: 10.1038/s41467-026-73287-7 (PMC13402297; doi:10.1038/s41467-026-73287-7)
Supplement: Supplementary file 2 — Description of Additional Supplementary Files [file 41467_2026_73287_MOESM2_ESM.pdf]

<u>Description of Additional Supplementary Files</u>

Supplementary Data 1: List of antibodies
