# Supplementary material for: Macrophage-derived fibronectin suppresses antitumor immunity via tissue stiffening and immunosuppressive cell induction in cancer mouse models
Source: Nat Commun. 2026 May 22;17:7296. doi: 10.1038/s41467-026-73287-7 (PMC13402297; doi:10.1038/s41467-026-73287-7)
Supplement: Supplementary file 4 — Reporting Summary [file 41467_2026_73287_MOESM4_ESM.pdf]

nature portfolio

Corresponding author(s): Li Yang, Yi Zhang

Last updated by author(s): Mar 20, 2026

Reporting Summary

Nature Portfolio wishes to improve the reproducibility of the work that we publish. This form provides structure for consistency and transparency in reporting. For further information on Nature Portfolio policies, see our Editorial Policies and the Editorial Policy Checklist.

Statistics

For all statistical analyses, confirm that the following items are present in the figure legend, table legend, main text, or Methods section.

n/a	Confirmed	
☐	☒	The exact sample size (*n*) for each experimental group/condition, given as a discrete number and unit of measurement
☐	☒	A statement on whether measurements were taken from distinct samples or whether the same sample was measured repeatedly
☐	☒	The statistical test(s) used AND whether they are one- or two-sided<br>*Only common tests should be described solely by name; describe more complex techniques in the Methods section.*
☒	☐	A description of all covariates tested
☒	☐	A description of any assumptions or corrections, such as tests of normality and adjustment for multiple comparisons
☐	☒	A full description of the statistical parameters including central tendency (e.g. means) or other basic estimates (e.g. regression coefficient) AND variation (e.g. standard deviation) or associated estimates of uncertainty (e.g. confidence intervals)
☐	☒	For null hypothesis testing, the test statistic (e.g. *F*, *t*, *r*) with confidence intervals, effect sizes, degrees of freedom and *P* value noted<br>*Give P values as exact values whenever suitable.*
☒	☐	For Bayesian analysis, information on the choice of priors and Markov chain Monte Carlo settings
☒	☐	For hierarchical and complex designs, identification of the appropriate level for tests and full reporting of outcomes
☐	☒	Estimates of effect sizes (e.g. Cohen's *d*, Pearson's *r*), indicating how they were calculated

Our web collection on statistics for biologists contains articles on many of the points above.

Software and code

Policy information about availability of computer code

Data collection	No special or proprietary software was used.
Data analysis	The following software was used in this study: ImageJ v1.54p, GraphPad Prism v8.0.1/v7.0, FlowJo v10.8.1, SeekSoul Tools v1.2.1, MobiVision v3.0, Seurat v.4.0, Seurat v5.1.0, SingleR v2.0.0, Living Image v4.4, Dr.Tom v2.0, Wave v2.6., DEAS 3.0., QuPath v0.3.0., ZEN version 3.6.

For manuscripts utilizing custom algorithms or software that are central to the research but not yet described in published literature, software must be made available to editors and reviewers. We strongly encourage code deposition in a community repository (e.g. GitHub). See the Nature Portfolio guidelines for submitting code & software for further information.

Data

Policy information about availability of data

All manuscripts must include a data availability statement. This statement should provide the following information, where applicable:

- Accession codes, unique identifiers, or web links for publicly available datasets
- A description of any restrictions on data availability
- For clinical datasets or third party data, please ensure that the statement adheres to our policy

Single-cell RNA-seq and bulk RNA sequencing data have been deposited in the Genome Sequence Archive at the National Genomics Data Center, China National Center for Bioinformation/Beijing Institute of Genomics, Chinese Academy of Sciences. Human data are accessible through GSA-human: HRA006716 (https://ngdc.cncb.ac.cn/gsa-human/browse/HRA006716), HRA009508 (https://ngdc.cncb.ac.cn/gsa-human/browse/HRA009508) and HRA009520 (https://ngdc.cncb.ac.cn/gsa-human/browse/HRA009520). According to Chinese regulatory requirements, all human sequencing data must be subject to controlled access. Applicants can

follow the steps in the document (https://ngdc.cncb.ac.cn/gsa-human/document/GSA-Human_Request_Guide_for_Users_us.pdf) to apply for access. Mouse data are accessible through GSA: CRA020886 (https://ngdc.cncb.ac.cn/gsa/browse/CRA020886) and are publicly available as of the date of publication. The western blotting images and microscopic data reported in this paper have been deposited in Figshare (https://doi.org/10.6084/m9.figshare.28211138). All data are included in the Supplementary Information or available from the authors, as are unique reagents used in this Article. The raw numbers for charts and graphs are available in the Source Data file whenever possible. Source Data are provided with this paper.

Research involving human participants, their data, or biological material

Policy information about studies with human participants or human data. See also policy information about sex, gender (identity/presentation), and sexual orientation and race, ethnicity and racism.

Reporting on sex and gender	Information about human participants has been listed in the Supplementary Table 1. Neither sex nor age-related issues are applied to this analysis.
Reporting on race, ethnicity, or other socially relevant groupings	Data on race, ethnicity and other socially relevant groupings were not collected in this study.
Population characteristics	Sixteen patients with NSCLC were enrolled in this study at The First Affiliated Hospital of Zhengzhou University. Information about human participants has been listed in the Supplementary Table 1.
Recruitment	For analyze the characteristics of macrophages in tumor microenvironment, patients with cancer included both females (n = 12) and males (n = 4) ranging in age from 42 to 78 years were recruited in the First Affiliated Hospital of Zhengzhou University. Patients or their guardians signed the informed consent. No bias in the selection.
Ethics oversight	The study was approved by the Scientific Research and Clinical Trial Ethics Committee of the First Affiliated Hospital of Zhengzhou University (No. 2019-KY-256).

Note that full information on the approval of the study protocol must also be provided in the manuscript.

Field-specific reporting

Please select the one below that is the best fit for your research. If you are not sure, read the appropriate sections before making your selection.

[x] Life sciences [ ] Behavioural & social sciences [ ] Ecological, evolutionary & environmental sciences

For a reference copy of the document with all sections, see nature.com/documents/nr-reporting-summary-flat.pdf

Life sciences study design

All studies must disclose on these points even when the disclosure is negative.

Sample size	No statistical analysis was computed to determine sample size for all experiments. Sample size was determined based on our experiences and previous published works (Yang L, Dong Y, Li Y, Wang D, Liu S, Wang D, Gao Q, Ji S, Chen X, Lei Q, Jiang W, Wang L, Zhang B, Yu JJ, Zhang Y (2019). "IL-10 derived from M2 macrophage promotes cancer stemness via JAK1/STAT1/NF-kB/Notch1 pathway in non-small cell lung cancer." Int J Cancer, 145(4):1099-1110. Cao, H., C. Ni, L. Han, R. Wang, R. Blasig, R. Haseloff, Y. Qin, J. Lan, X. Lou, P. Ma, X. Yao, L. Wang, F. Wang, L. Zhu, N. Lei, I. E. Blasig and Z. Qin (2022). "Claudin-12 Deficiency Inhibits Tumor Growth by Impairing Transendothelial Migration of Myeloid-Derived Suppressor Cells." Cancer Res 82(13): 2472-2484.). Sample sizes are described in greater detail in the Figures or Figure Legends.
Data exclusions	No data were excluded from the analyses.
Replication	All experiments were independently replicated at least three times to ensure reproducibility. For key findings, biological replicates (n=3-5) and technical replicates (n=2-3 per biological sample) were performed. All attempts at replication were successful.
Randomization	For in vitro experiments, cells were seeded into plates with consistent cell density and uniform spatial distribution. Treatment groups were randomly assigned to well positions. For in vivo experiments, mice were stratified by baseline weight (±10%) and sex before random assignment to treatment groups.
Blinding	The investigators were not blinded during the animal experiments. Blinding was not performed because the sex of mice required identification for housing purpose. Daily monitoring of mice reduced the ability for blinding. For downstream experimentation on samples derived from in vivo experiments the investigator organizing the experimental groups and involved in sample collection was not blinded; colleagues aiding in data collection were blinded. For in vitro experiments, the investigators were not blinded for group allocation as the same investigator both planned and performed the experiment. For the rest of the experiments, blinding was not necessary because the readout was automated i.e. qPCR, or run through R i.e. scRNAseq, and the experimenter needs to know the corresponding conditions.

Behavioural & social sciences study design

All studies must disclose on these points even when the disclosure is negative.

Study description	*Briefly describe the study type including whether data are quantitative, qualitative, or mixed-methods (e.g. qualitative cross-sectional, quantitative experimental, mixed-methods case study).*
Research sample	*State the research sample (e.g. Harvard university undergraduates, villagers in rural India) and provide relevant demographic information (e.g. age, sex) and indicate whether the sample is representative. Provide a rationale for the study sample chosen. For studies involving existing datasets, please describe the dataset and source.*
Sampling strategy	*Describe the sampling procedure (e.g. random, snowball, stratified, convenience). Describe the statistical methods that were used to predetermine sample size OR if no sample-size calculation was performed, describe how sample sizes were chosen and provide a rationale for why these sample sizes are sufficient. For qualitative data, please indicate whether data saturation was considered, and what criteria were used to decide that no further sampling was needed.*
Data collection	*Provide details about the data collection procedure, including the instruments or devices used to record the data (e.g. pen and paper, computer, eye tracker, video or audio equipment) whether anyone was present besides the participant(s) and the researcher, and whether the researcher was blind to experimental condition and/or the study hypothesis during data collection.*
Timing	*Indicate the start and stop dates of data collection. If there is a gap between collection periods, state the dates for each sample cohort.*
Data exclusions	*If no data were excluded from the analyses, state so OR if data were excluded, provide the exact number of exclusions and the rationale behind them, indicating whether exclusion criteria were pre-established.*
Non-participation	*State how many participants dropped out/declined participation and the reason(s) given OR provide response rate OR state that no participants dropped out/declined participation.*
Randomization	*If participants were not allocated into experimental groups, state so OR describe how participants were allocated to groups, and if allocation was not random, describe how covariates were controlled.*

Ecological, evolutionary & environmental sciences study design

All studies must disclose on these points even when the disclosure is negative.

Study description	*Briefly describe the study. For quantitative data include treatment factors and interactions, design structure (e.g. factorial, nested, hierarchical), nature and number of experimental units and replicates.*
Research sample	*Describe the research sample (e.g. a group of tagged Passer domesticus, all Stenocereus thurberi within Organ Pipe Cactus National Monument), and provide a rationale for the sample choice. When relevant, describe the organism taxa, source, sex, age range and any manipulations. State what population the sample is meant to represent when applicable. For studies involving existing datasets, describe the data and its source.*
Sampling strategy	*Note the sampling procedure. Describe the statistical methods that were used to predetermine sample size OR if no sample-size calculation was performed, describe how sample sizes were chosen and provide a rationale for why these sample sizes are sufficient.*
Data collection	*Describe the data collection procedure, including who recorded the data and how.*
Timing and spatial scale	*Indicate the start and stop dates of data collection, noting the frequency and periodicity of sampling and providing a rationale for these choices. If there is a gap between collection periods, state the dates for each sample cohort. Specify the spatial scale from which the data are taken*
Data exclusions	*If no data were excluded from the analyses, state so OR if data were excluded, describe the exclusions and the rationale behind them, indicating whether exclusion criteria were pre-established.*
Reproducibility	*Describe the measures taken to verify the reproducibility of experimental findings. For each experiment, note whether any attempts to repeat the experiment failed OR state that all attempts to repeat the experiment were successful.*
Randomization	*Describe how samples/organisms/participants were allocated into groups. If allocation was not random, describe how covariates were controlled. If this is not relevant to your study, explain why.*
Blinding	*Describe the extent of blinding used during data acquisition and analysis. If blinding was not possible, describe why OR explain why blinding was not relevant to your study.*

Did the study involve field work? ☐ Yes ☐ No

Field work, collection and transport

Field conditions	*Describe the study conditions for field work, providing relevant parameters (e.g. temperature, rainfall).*
Location	*State the location of the sampling or experiment, providing relevant parameters (e.g. latitude and longitude, elevation, water depth).*
Access & import/export	*Describe the efforts you have made to access habitats and to collect and import/export your samples in a responsible manner and in compliance with local, national and international laws, noting any permits that were obtained (give the name of the issuing authority, the date of issue, and any identifying information).*
Disturbance	*Describe any disturbance caused by the study and how it was minimized.*

Reporting for specific materials, systems and methods

We require information from authors about some types of materials, experimental systems and methods used in many studies. Here, indicate whether each material, system or method listed is relevant to your study. If you are not sure if a list item applies to your research, read the appropriate section before selecting a response.

Materials & experimental systems

n/a	Involved in the study	
☐	☒	Antibodies
☐	☒	Eukaryotic cell lines
☒	☐	Palaeontology and archaeology
☐	☒	Animals and other organisms
☒	☐	Clinical data
☒	☐	Dual use research of concern
☒	☐	Plants

Methods

n/a	Involved in the study	
☒	☐	ChIP-seq
☐	☒	Flow cytometry
☒	☐	MRI-based neuroimaging

Antibodies

Antibodies used

PerCP/Cyanine5.5 anti-mouse F4/80 (BioLegend Cat# 123127; RRID: AB_893484); 1:100
Brilliant Violet 605 anti-mouse F4/80 Antibody (BioLegend Cat# 123133; RRID: AB_2562305); 1:100
Alexa Fluor 700 anti-mouse/human CD11b (BioLegend Cat# 101222; RRID: AB_493705); 1:100
PE anti-mouse CD206 (MMR) (BioLegend Cat# 141706; RRID: AB_10895754); 1:100
Brilliant Violet 421 anti-mouse CD86 (BioLegend Cat# 105032; RRID: AB_10898329); 1:100
Brilliant Violet 605 anti-mouse CD274 (B7-H1, PD-L1) (BioLegend Cat# 124321; RRID: AB_2563635); 1:100
APC anti-mouse IL-6 (BioLegend Cat# 504508; RRID: AB_10694868); 1:100
Brilliant Violet 785 anti-mouse TNF-a (BioLegend Cat# 506341; RRID: AB_2565951); 1:100
PE anti-mouse CD45 (BioLegend Cat# 103106; RRID: AB_312970); 1:100
FITC anti-mouse CD8a (BioLegend Cat# 100804; RRID: AB_312764); 1:100
APC anti-mouse CD279 (PD-1) (BioLegend Cat# 135210; RRID: AB_2159183); 1:100
Brilliant Violet 605 anti-mouse IFN-g (BioLegend Cat# 505840; RRID: AB_2561438); 1:100
APC anti-mouse Perforin (BioLegend Cat# 154304; RRID: AB_2721462); 1:100
Brilliant Violet 421 anti-human/mouse Granzyme B Recombinant (BioLegend Cat# 396413; RRID: AB_2810603); 1:100
APC anti-human CD206 (BioLegend Cat# 321110; RRID: AB_571884); 1:100
APC/Cyanine7 anti-human CD14 (BioLegend Cat# 325620; RRID: AB_830693); 1:100
Brilliant Violet 650 anti-human CD14 (BioLegend Cat# 301835; RRID: AB_11204241); 1:100
PE anti-human CD163 (BioLegend Cat# 326506; RRID: AB_893269); 1:100
APC anti-human IL-6 (BioLegend Cat# 501112; RRID: AB_389222); 1:100
FITC anti-human IL-1b (BioLegend Cat# 511705; RRID: AB_1236434); 1:100
Brilliant Violet 605 anti-human TNF-a Antibody (BioLegend Cat# 502936; RRID: AB_2563884); 1:100
CXCL9 Recombinant Rabbit Monoclonal Antibody (11H1L14) Invitrogen Cat# 701117; RRID: AB_2532396;
CXCL10 Recombinant Rabbit Monoclonal Antibody (10H11L3) Invitrogen Cat# 701225; RRID: AB_2532429; 1:300
Anti-CD68 antibody (SP251) Abcam Cat# ab192847; RRID: AB_3076701; 1:300
Anti-CD163 antibody (EPR19518) Abcam Cat# ab182422; RRID: AB_2753196; 1:300
F4/80 (D2S9R) XP Rabbit mAb Cell Signaling Technology Cat# 70076; RRID: AB_2799771; 1: 200
Anti-Fibronectin antibody (F1) Abcam Cat# ab32419; RRID: AB_732379; 1:100 for FC; 1:1000 for WB; 1: 200 for mIHC
Anti-PFKP antibody (OTI1D6) Abcam Cat# ab119796; RRID: AB_10903593; 1:100 for IF; 1:1000 for WB; 1: 200 for mIHC
Anti-PFKP antibody (EPR17314) Abcam Cat# ab204131; RRID: AB_2941850; 1:2000 for WB
Anti-SQSTM1/p62 antibody (EPR4811) Abcam Cat# ab109012; RRID: AB_2810880; 1:10000 for WB; 1: 200 for mIHC
Anti-CD20 antibody (SP32) Abcam Cat# ab64088; RRID: AB_1139386; 1:100
CD8α (D4W2Z) XP Rabbit mAb Cell Signaling Technology Cat# 98941; RRID: AB_2756376; 1:200

CD4 (D7D2Z) Rabbit mAb Cell Signaling Technology Cat# 25229; RRID: AB_2798898; 1:100
Anti-NKR-P1C antibody (EPR22990-31) Abcam Cat# ab289542; RRID: AB_3094493; 1:100
b-Actin (8H10D10) Mouse mAb Cell Signaling Technology Cat# 3700S; RRID: AB_2242334; 1:2000
LC3A/B (D3U4C) XP Rabbit mAb Cell Signaling Technology Cat# 12741; RRID: AB_2617131; 1:1000
LC3B (E7X4S) XP Rabbit mAb Cell Signaling Technology Cat# 43566; RRID: AB_2910544; 1:10000 for IF; 1:1000 for WB
LC3B (E5Q2K) XP Mouse mAb Cell Signaling Technology Cat# 83506; RRID: AB_2800018; 1:400 for IF
Phospho-mTOR (Ser2448) (D9c2) XP Rabbit mAb Cell Signaling Technology Cat# 5536; RRID: AB_10691552; 1:1000
mTOR (7C10) Rabbit mAb Cell Signaling Technology Cat# 2983; RRID: AB_2105622; 1:1000 for WB; 1:200 for IF
Phospho-4E-BP1 (Thr37/46) (236B4) Rabbit mAb Cell Signaling Technology Cat# 2855; RRID: AB_2097838; 1:1000
4E-BP1 (53H11) Rabbit mAb Cell Signaling Technology Cat# 9644; RRID: AB_2097841; 1:1000
Phospho-S6 Ribosomal Protein (Ser235/236) (D57.2.2E) XP Rabbit mAb Cell Signaling Technology Cat# 4858; RRID: AB_916156; 1:1000
S6 Ribosomal Protein (5G10) Rabbit mAb Cell Signaling Technology Cat# 2217; RRID: AB_ 331355; 1:1000
Mouse anti-RAC1 Monoclonal antibody(1301CT276.121.104) Absin Bioscience Cat# abs100465; ; 1:1000 for WB; 1:200 for IF
Anti-mouse CD3ε-InVivo (145-2C11) Selleck Cat# A2104
Anti-mouse CD28-InVivo (PV-1) Selleck Cat# A2108
Anti-mouse PD-1 (CD279)-InVivo (RMP1-14) Selleck Cat# A2122; RRID: AB_364424
Rat IgG2a isotype control-InVivo (2A3) Selleck Cat# A2123; RRID: AB_3644245;
Rabbit anti-IL-1Beta Polycolon Antibody Absin Bioscience Cat# abs120224; 1:200
Goat Anti-Rabbit IgG H&L (Alexa Fluor 488) Abcam Cat# ab150077; RRID: AB_2630356;

Validation

PerCP/Cyanine5.5 anti-mouse F4/80 (BioLegend Cat# 123127; RRID: AB_893484); 1:100 Validated by manufacturer in BALB/c mouse peritoneal macrophages
Brilliant Violet 605 anti-mouse F4/80 Antibody (BioLegend Cat# 123133; RRID: AB_2562305); 1:100 Validated by manufacturer in
Alexa Fluor 700 anti-mouse/human CD11b (BioLegend Cat# 101222; RRID: AB_493705); 1:100 Validated by manufacturer in C57BL/6 mouse bone marrow cells
PE anti-mouse CD206 (MMR) (BioLegend Cat# 141706; RRID: AB_10895754); 1:100 Validated by manufacturer in thioglycollate-elicited BALB/c peritoneal macrophages
Brilliant Violet 421 anti-mouse CD86 (BioLegend Cat# 105032; RRID: AB_10898329); 1:100 Validated by manufacturer in LPS-stimulated (3 days) mouse splenocytes
Brilliant Violet 605 anti-mouse CD274 (B7-H1, PD-L1) (BioLegend Cat# 124321; RRID: AB_2563635); 1:100 Validated by manufacturer in C57BL/6 mouse splenocytes
APC anti-mouse IL-6 (BioLegend Cat# 504508; RRID: AB_10694868); 1:100 Validated by manufacturer in thioglycollate-elicited C57BL/6 mouse peritoneal macrophages
Brilliant Violet 785 anti-mouse TNF-a (BioLegend Cat# 506341; RRID: AB_2565951); 1:100 Validated by manufacturer in PMA + Ionomycin-stimulated C57BL/6 mouse splenocytes
PE anti-mouse CD45 (BioLegend Cat# 103106; RRID: AB_312970); 1:100 Validated by manufacturer in C57BL/6 mouse splenocytes
FITC anti-mouse CD8a (BioLegend Cat# 100804; RRID: AB_312764); 1:100 Validated by manufacturer in C57BL/6 mouse splenocytes
APC anti-mouse CD279 (PD-1) (BioLegend Cat# 135210; RRID: AB_2159183); 1:100 Validated by manufacturer in Con-A and IL-2 stimulated C57BL/6 splenocytes (3 days)
Brilliant Violet 605 anti-mouse IFN-g (BioLegend Cat# 505840; RRID: AB_2561438); 1:100 Validated by manufacturer in PMA +ionomycin-stimulated C57BL/6 mouse splenocytes
APC anti-mouse Perforin (BioLegend Cat# 154304; RRID: AB_2721462); 1:100 Validated by manufacturer in C57BL/6 splenocytes
Brilliant Violet 421 anti-human/mouse Granzyme B Recombinant (BioLegend Cat# 396413; RRID: AB_2810603); 1:100 Validated by manufacturer in human peripheral blood mononuclear cells
APC anti-human CD206 (BioLegend Cat# 321110; RRID: AB_571884); 1:100 Validated by manufacturer in GM-CSF- stimulated (day-3) human monocytes
APC/Cyanine7 anti-human CD14 (BioLegend Cat# 325620; RRID: AB_830693); 1:100 Validated by manufacturer in human peripheral blood monocytes
Brilliant Violet 650 anti-human CD14 (BioLegend Cat# 301835; RRID: AB_11204241); 1:100 Validated by manufacturer in human peripheral blood monocytes
PE anti-human CD163 (BioLegend Cat# 326506; RRID: AB_893269); 1:100 Validated by manufacturer in human peripheral blood monocytes
APC anti-human IL-6 (BioLegend Cat# 501112; RRID: AB_389222); 1:100 Validated by manufacturer in LPS-stimulated (6 hours) human peripheral blood monocytes
FITC anti-human IL-1b (BioLegend Cat# 511705; RRID: AB_1236434); 1:100 Validated by manufacturer in LPS-stimulated peripheral blood mononuclear cells
Brilliant Violet 605 anti-human TNF-a Antibody (BioLegend Cat# 502936; RRID: AB_2563884); 1:100 Validated by manufacturer in PMA+ionomycin stimulated (6 hours) human peripheral blood lymphocytes
CXCL9 Recombinant Rabbit Monoclonal Antibody (11H1L14) Invitrogen Cat# 701117; RRID: AB_2532396; 1: 100 Validated by manufacturer in THP-1 cells
CXCL10 Recombinant Rabbit Monoclonal Antibody (10H11L3) Invitrogen Cat# 701225; RRID: AB_2532429; 1:300 Validated by manufacturer in HT-29 cells
Anti-CD68 antibody (SP251) Abcam Cat# ab192847; RRID: AB_3076701; 1:300 Validated by manufacturer in normal human tonsil tissue
Anti-CD163 antibody (EPR19518) Abcam Cat# ab182422; RRID: AB_2753196; 1:300 Validated by manufacturer in human breast carcinoma tissue
F4/80 (D2S9R) XP Rabbit mAb Cell Signaling Technology Cat# 70076; RRID: AB_2799771; 1: 200 Validated by manufacturer in mouse liver

Anti-Fibronectin antibody (F1) Abcam Cat# ab32419; RRID: AB_732379; 1:100 for FC; 1:1000 for WB; 1: 200 for mIHC Validated by manufacturer in HepG2 (Human liver hepatocellular carcinoma cell line) cells
Anti-PFKP antibody (OTI1D6) Abcam Cat# ab119796; RRID: AB_10903593; 1:100 for IF; 1:1000 for WB; 1: 200 for mIHC Validated by manufacturer in COS7 cells
Anti-PFKP antibody (EPR17314) Abcam Cat# ab204131; RRID: AB_2941850; 1:2000 for WB Validated by manufacturer in MCF7 (Human breast adenocarcinoma cell line)
Anti-SQSTM1/p62 antibody (EPR4811) Abcam Cat# ab109012; RRID: AB_2810880; 1:10000 for WB; 1: 200 for mIHC Validated by manufacturer in U-2 OS cells
Anti-CD20 antibody (SP32) Abcam Cat# ab64088; RRID: AB_1139386; 1:100 Validated by manufacturer in human tonsil tissue
CD8α (D4W2Z) XP Rabbit mAb Cell Signaling Technology Cat# 98941; RRID: AB_2756376; 1:200 Validated by manufacturer in mouse spleen
CD4 (D7D2Z) Rabbit mAb Cell Signaling Technology Cat# 25229; RRID: AB_2798898; 1:100 Validated by manufacturer in 4T1 metastatic tumor
Anti-NKR-P1C antibody (EPR22990-31) Abcam Cat# ab289542; RRID: AB_3094493; 1:100 Validated by manufacturer in mouse spleen cells
b-Actin (8H10D10) Mouse mAb Cell Signaling Technology Cat# 3700S; RRID: AB_2242334; 1:2000 Validated by manufacturer in HeLa cells
LC3A/B (D3U4C) XP Rabbit mAb Cell Signaling Technology Cat# 12741; RRID: AB_2617131; 1:1000 Validated by manufacturer in RD cells
LC3B (E7X4S) XP Rabbit mAb Cell Signaling Technology Cat# 43566; RRID: AB_2910544; 1:10000 for IF; 1:1000 for WB Validated by manufacturer in HCT 116
LC3B (E5Q2K) XP Mouse mAb Cell Signaling Technology Cat# 83506; RRID: AB_2800018; 1:400 for IF Validated by manufacturer in HCT 116
Phospho-mTOR (Ser2448) (D9c2) XP Rabbit mAb Cell Signaling Technology Cat# 5536; RRID: AB_10691552; 1:1000 Validated by manufacturer in NIH/3T3 cells
mTOR (7C10) Rabbit mAb Cell Signaling Technology Cat# 2983; RRID: AB_2105622; 1:1000 for WB; 1:200 for IF Validated by manufacturer in HeLa cells
Phospho-4E-BP1 (Thr37/46) (236B4) Rabbit mAb Cell Signaling Technology Cat# 2855; RRID: AB_2097838; 1:1000 Validated by manufacturer in 293T cells
4E-BP1 (53H11) Rabbit mAb Cell Signaling Technology Cat# 9644; RRID: AB_2097841; 1:1000 Validated by manufacturer in HeLa cells
Phospho-S6 Ribosomal Protein (Ser235/236) (D57.2.2E) XP Rabbit mAb Cell Signaling Technology Cat# 4858; RRID: AB_916156; 1:1000 Validated by manufacturer in
S6 Ribosomal Protein (5G10) Rabbit mAb Cell Signaling Technology Cat# 2217; RRID: AB_ 331355; 1:1000 Validated by manufacturer in MCF7 cells
Mouse anti-RAC1 Monoclonal antibody(1301CT276.121.104) Absin Bioscience Cat# abs100465; 1:1000 for WB; 1:200 for IF Validated by manufacturer in paraffin-embedded H.skin section
Anti-mouse CD3ε-InVivo (145-2C11) Selleck Cat# A2104
Anti-mouse CD28-InVivo (PV-1) Selleck Cat# A2108
Anti-mouse PD-1 (CD279)-InVivo (RMP1-14) Selleck Cat# A2122; RRID: AB_364424
Rat IgG2a isotype control-InVivo (2A3) Selleck Cat# A2123; RRID: AB_3644245
Rabbit anti-IL-1Beta Polycolon Antibody Absin Bioscience Cat# abs120224; 1:200
Goat Anti-Rabbit IgG H&L (Alexa Fluor 488) Abcam Cat# ab150077; RRID: AB_2630356

Eukaryotic cell lines

Policy information about cell lines and Sex and Gender in Research

Cell line source(s)
LLC (Wuhan Pricella Biotechnology Co., Ltd, CL-0140), THP-1 (National Collection of Authenticated Cell Cultures, TCHu57). CD14+ monocytes were sorted from peripheral blood of healthy donors. Mouse BMDMs were obtained by flushing the femurs of male and female C57BL/6 mice. Mouse CD8+ T cells were dissociated from spleens of female C57BL/6 mice.

Authentication
LLC, THP-1 were authenticated using STR profiling. Macrophages and CD8+ T cells were authenticated by morphology and expression of cell-associated markers using flow cytometry.

Mycoplasma contamination
All cell lines were tested negative for mycoplasma contamination.

Commonly misidentified lines
(See ICLAC register)
No commonly misidentified cell lines were used.

Palaeontology and Archaeology

Specimen provenance
Provide provenance information for specimens and describe permits that were obtained for the work (including the name of the issuing authority, the date of issue, and any identifying information). Permits should encompass collection and, where applicable, export.

Specimen deposition
Indicate where the specimens have been deposited to permit free access by other researchers.

Dating methods	*If new dates are provided, describe how they were obtained (e.g. collection, storage, sample pretreatment and measurement), where they were obtained (i.e. lab name), the calibration program and the protocol for quality assurance OR state that no new dates are provided.*

☐ Tick this box to confirm that the raw and calibrated dates are available in the paper or in Supplementary Information.

Ethics oversight	*Identify the organization(s) that approved or provided guidance on the study protocol, OR state that no ethical approval or guidance was required and explain why not.*

Note that full information on the approval of the study protocol must also be provided in the manuscript.

Animals and other research organisms

Policy information about studies involving animals; ARRIVE guidelines recommended for reporting animal research, and Sex and Gender in Research

Laboratory animals	C57BL/6 mice (Charles River, 219) aged 6–8 weeks were purchased from Charles River (Beijing, China). The FN1fl/fl and FN1Lyz2 mice were purchased from Cyagen (Suzhou, China). Macrophage-specific FN1 knockout mice (FN1Lyz2, heterozygous for Lyz2-Cre) were generated by crossing FN1fl/fl mice (Cyagen, S-CKO-02465) with Lyz2-cre mice (Cyagen, C001003), and their F1 heterozygotes were crossed with FN1fl/fl mice. To minimize environmental variables, we utilized littermate controls generated by crossing FN1fl/fl with FN1ΔLyz2 mice (heterozygous for Lyz2-Cre), yielding litters with approximately 50% FN1fl/fl and 50% FN1ΔLyz2 pups. All mice were genotyped by PCR and agarose gel electrophoresis using genomic DNA extracted from tail biopsy tissues. All experiments employed age-matched (6–8 weeks old) and sex-matched littermates housed in the same facility.
Wild animals	No wild animals were used in this study.
Reporting on sex	Female wild-type C57BL/6 mice were involved in the study. For conditional knockout mice, all experiments employed age-matched (6-8 weeks old) and sex-matched littermates housed in the same facility to ensure consistency. Figures 2–6 were conducted using female mice only; Figure 7 (combination therapy with anti-PD-1) were conducted twice using male and female mice separately. No sex-based analyses were performed due to uniform experimental conditions.
Field-collected samples	All mice were housed at 21 ± 2°C and 40–60% humidity, on a 12 h light/dark cycle in a specific pathogen-free facility. In accordance with the guidelines of the Laboratory Animal Welfare Ethics Committee of Zhengzhou University Laboratory Animal Center, the maximum allowable tumor size was 1,500 mm^3, and this limit was not exceeded in any of the experiments. Mice were humanely euthanized if tumor volume exceeded the ethical limit, if body weight loss exceeded 20%, or if animals showed signs of ulceration, impaired mobility, or severe distress. All euthanasia procedures were performed using CO_2 inhalation followed by cervical dislocation in accordance with animal welfare guidelines.
Ethics oversight	Animal protocols were approved by the Laboratory Animal Welfare Ethics Committee of Zhengzhou University Laboratory Animal Center.

Note that full information on the approval of the study protocol must also be provided in the manuscript.

Clinical data

Policy information about clinical studies

All manuscripts should comply with the ICMJE guidelines for publication of clinical research and a completed CONSORT checklist must be included with all submissions.

Clinical trial registration	*Provide the trial registration number from ClinicalTrials.gov or an equivalent agency.*
Study protocol	*Note where the full trial protocol can be accessed OR if not available, explain why.*
Data collection	*Describe the settings and locales of data collection, noting the time periods of recruitment and data collection.*
Outcomes	*Describe how you pre-defined primary and secondary outcome measures and how you assessed these measures.*

Dual use research of concern

Policy information about dual use research of concern

Hazards

Could the accidental, deliberate or reckless misuse of agents or technologies generated in the work, or the application of information presented in the manuscript, pose a threat to:

No	Yes	
☐	☐	Public health
☐	☐	National security
☐	☐	Crops and/or livestock
☐	☐	Ecosystems
☐	☐	Any other significant area

Experiments of concern

Does the work involve any of these experiments of concern:

No	Yes	
☐	☐	Demonstrate how to render a vaccine ineffective
☐	☐	Confer resistance to therapeutically useful antibiotics or antiviral agents
☐	☐	Enhance the virulence of a pathogen or render a nonpathogen virulent
☐	☐	Increase transmissibility of a pathogen
☐	☐	Alter the host range of a pathogen
☐	☐	Enable evasion of diagnostic/detection modalities
☐	☐	Enable the weaponization of a biological agent or toxin
☐	☐	Any other potentially harmful combination of experiments and agents

Plants

Seed stocks

Report on the source of all seed stocks or other plant material used. If applicable, state the seed stock centre and catalogue number. If plant specimens were collected from the field, describe the collection location, date and sampling procedures.

Novel plant genotypes

Describe the methods by which all novel plant genotypes were produced. This includes those generated by transgenic approaches, gene editing, chemical/radiation-based mutagenesis and hybridization. For transgenic lines, describe the transformation method, the number of independent lines analyzed and the generation upon which experiments were performed. For gene-edited lines, describe the editor used, the endogenous sequence targeted for editing, the targeting guide RNA sequence (if applicable) and how the editor was applied.

Authentication

Describe any authentication procedures for each seed stock used or novel genotype generated. Describe any experiments used to assess the effect of a mutation and, where applicable, how potential secondary effects (e.g. second site T-DNA insertions, mosiacism, off-target gene editing) were examined.

ChIP-seq

Data deposition

☐ Confirm that both raw and final processed data have been deposited in a public database such as GEO.

☐ Confirm that you have deposited or provided access to graph files (e.g. BED files) for the called peaks.

Data access links
May remain private before publication.

For "Initial submission" or "Revised version" documents, provide reviewer access links. For your "Final submission" document, provide a link to the deposited data.

Files in database submission

Provide a list of all files available in the database submission.

Genome browser session
(e.g. UCSC)

Provide a link to an anonymized genome browser session for "Initial submission" and "Revised version" documents only, to enable peer review. Write "no longer applicable" for "Final submission" documents.

Methodology

Replicates

Describe the experimental replicates, specifying number, type and replicate agreement.

Sequencing depth

Describe the sequencing depth for each experiment, providing the total number of reads, uniquely mapped reads, length of reads and whether they were paired- or single-end.

Antibodies

Describe the antibodies used for the ChIP-seq experiments; as applicable, provide supplier name, catalog number, clone name, and lot number.

Peak calling parameters

Specify the command line program and parameters used for read mapping and peak calling, including the ChIP, control and index files used.

Data quality

Describe the methods used to ensure data quality in full detail, including how many peaks are at FDR 5% and above 5-fold enrichment.

Software | *Describe the software used to collect and analyze the ChIP-seq data. For custom code that has been deposited into a community repository, provide accession details.*

Flow Cytometry

Plots

Confirm that:

- [x] The axis labels state the marker and fluorochrome used (e.g. CD4-FITC).
- [x] The axis scales are clearly visible. Include numbers along axes only for bottom left plot of group (a 'group' is an analysis of identical markers).
- [x] All plots are contour plots with outliers or pseudocolor plots.
- [x] A numerical value for number of cells or percentage (with statistics) is provided.

Methodology

Sample preparation	Details of sample preparation, including the source of cells and methods such as tissue processing have been described in detail in Methods.<br>Tumor samples were mechanically minced into small pieces with a sterile scalpel and further dissociated into single-cell suspensions using a Tumor Dissociation Kit (Miltenyi Biotec) following the manufacturer's instructions. The suspensions were filtered through a 70 um strainer and centrifuged at 300 ×g for 10 min at 4 °C. Cell count and viability were estimated using a fluorescence Cell Analyzer (Countstar Rigel S2) with AO/PI reagent after removal of erythrocytes (Miltenyi Biotec). Finally, the suspensions were washed twice with PBS and diluted to the appropriate concentrations for flow cytometry, library construction, and sequencing. Mouse tumor tissues were disrupted into pieces and incubated with a tumor dissociation kit (Miltenyi Biotec) for 30 min at 37 °C. Subsequently, the suspensions were filtered through a 70um strainer and centrifuged at 500 ×g for 5 min at 4 °C.<br>CD14+ cells were isolated from peripheral blood mononuclear cells using MACS with human CD14 microbeads (Miltenyi Biotec), according to the manufacturer's instructions.<br>Cell surface molecules were stained for 20 min at 4°C in the dark using saturating concentrations of antibodies. For intracellular staining, cells were fixed with 4% formalin for 30 min and incubated with permeabilization washing buffer for 30 min. The cells were then analyzed using flow cytometry.
Instrument	CytoFLEX (Moflo-XDP; Beckman Coulter), BD FACS Canto II(BD Biosciences), DxFLEX B5-R3-V5(Beckman Coulter)
Software	FlowJo v10.8.1 and CytExpert for DxFLEX (https://www.beckmancoulter.cn/bls/bls_CytoFLEX/)
Cell population abundance	The number of target cells was 100000 or at least 50000 to ensure the accuracy of the experimental results.
Gating strategy	The gating strategy used for all relevant experiments was showed in Supplemental Figure 1, 3 and 8.

- [x] Tick this box to confirm that a figure exemplifying the gating strategy is provided in the Supplementary Information.

Magnetic resonance imaging

Experimental design

Design type	*Indicate task or resting state; event-related or block design.*
Design specifications	*Specify the number of blocks, trials or experimental units per session and/or subject, and specify the length of each trial or block (if trials are blocked) and interval between trials.*
Behavioral performance measures	*State number and/or type of variables recorded (e.g. correct button press, response time) and what statistics were used to establish that the subjects were performing the task as expected (e.g. mean, range, and/or standard deviation across subjects).*

Acquisition

Imaging type(s)	*Specify: functional, structural, diffusion, perfusion.*
Field strength	*Specify in Tesla*
Sequence & imaging parameters	*Specify the pulse sequence type (gradient echo, spin echo, etc.), imaging type (EPI, spiral, etc.), field of view, matrix size, slice thickness, orientation and TE/TR/flip angle.*
Area of acquisition	*State whether a whole brain scan was used OR define the area of acquisition, describing how the region was determined.*

Diffusion MRI - [ ] Used - [ ] Not used

Preprocessing

Preprocessing software | *Provide detail on software version and revision number and on specific parameters (model/functions, brain extraction, segmentation, smoothing kernel size, etc.).*

Normalization | *If data were normalized/standardized, describe the approach(es): specify linear or non-linear and define image types used for transformation OR indicate that data were not normalized and explain rationale for lack of normalization.*

Normalization template | *Describe the template used for normalization/transformation, specifying subject space or group standardized space (e.g. original Talairach, MNI305, ICBM152) OR indicate that the data were not normalized.*

Noise and artifact removal | *Describe your procedure(s) for artifact and structured noise removal, specifying motion parameters, tissue signals and physiological signals (heart rate, respiration).*

Volume censoring | *Define your software and/or method and criteria for volume censoring, and state the extent of such censoring.*

Statistical modeling & inference

Model type and settings | *Specify type (mass univariate, multivariate, RSA, predictive, etc.) and describe essential details of the model at the first and second levels (e.g. fixed, random or mixed effects; drift or auto-correlation).*

Effect(s) tested | *Define precise effect in terms of the task or stimulus conditions instead of psychological concepts and indicate whether ANOVA or factorial designs were used.*

Specify type of analysis: ☐ Whole brain ☐ ROI-based ☐ Both

Statistic type for inference | *Specify voxel-wise or cluster-wise and report all relevant parameters for cluster-wise methods.*

(See Eklund et al. 2016)

Correction | *Describe the type of correction and how it is obtained for multiple comparisons (e.g. FWE, FDR, permutation or Monte Carlo).*

Models & analysis

n/a	Involved in the study
☐	☐ Functional and/or effective connectivity
☐	☐ Graph analysis
☐	☐ Multivariate modeling or predictive analysis

Functional and/or effective connectivity | *Report the measures of dependence used and the model details (e.g. Pearson correlation, partial correlation, mutual information).*

Graph analysis | *Report the dependent variable and connectivity measure, specifying weighted graph or binarized graph, subject- or group-level, and the global and/or node summaries used (e.g. clustering coefficient, efficiency, etc.).*

Multivariate modeling and predictive analysis | *Specify independent variables, features extraction and dimension reduction, model, training and evaluation metrics.*
