# Supplementary material for: Macrophage-derived fibronectin suppresses antitumor immunity via tissue stiffening and immunosuppressive cell induction in cancer mouse models
Source: Nat Commun. 2026 May 22;17:7296. doi: 10.1038/s41467-026-73287-7 (PMC13402297; doi:10.1038/s41467-026-73287-7)
Supplement: Supplementary file 5 — Transparent Peer Review file [file 41467_2026_73287_MOESM5_ESM.pdf]

nature portfolio

Peer Review File

Macrophages-derived fibronectin suppresses anti-tumor immunity via tissue stiffening and immunosuppression induction

Corresponding Author: Professor Li Yang

This file contains all reviewer reports in order by version, followed by all author rebuttals in order by version.

Version 0:

Reviewer comments:

Reviewer #1

(Remarks to the Author)
In the manuscript "FN1+ macrophages prevent anti-tumor immunity via ECM stiffening and autophagy-mediated immunosuppression", Li et al. investigates how fibronectin (FN1)-producing tumor-associated macrophages (TAMs) promote immune suppression in non-small cell lung cancer (NSCLC). Using single-cell RNA sequencing, they find that FN1+ TAMs comprise a specific immunosuppressive macrophage subset. The authors suggest that FN1+ macrophages stiffen the ECM, creating an immunosuppressive ECM. Deletion of FN1 in TAMs using conditional knockout mice changes the cells towards a less immunosuppressive phenotype.
Mechanistically, the authors suggest that FN1 activates cytoskeletal assembly and autophagy through inhibition of the RAC1–mTOR pathway, suppressing macrophage glycolysis and promoting an anti-inflammatory phenotype. Finally, FN1 deletion in TAMs or treatment with a FN1 inhibitor leads to delayed tumor growth.
The paper contains a large amount of data and several of the findings are very interesting. Many observations are also very surprising warranting particularly thorough control experiments. Unfortunately, the paper lacks important information necessary for a thorough evaluation of the data, and additional essential experiments are required to support the claims of the paper.
Critique points:
1) The drastic difference between the TAMs in FN1fl/fl mice and FNLyz2 mice and the differences in tumor growth, makes it essential to know the details of the experimental mice that were compared (background, age, gender, breeding). Also, an additional control group consisting of the Lyz2-Cre mice should be included.
2) Additional information on the fibronectin inhibitor should be provided, including data that supports its use for in vivo experiments. Control experiments (in vivo and in vitro) demonstrating a fibronectin "inhibiting" function should be included.
3) Critical information regarding the flow cytometry experiments is lacking, including antibody information and information on whether individual markers (such as FN1) were detected intracellularly or on the cell surface.
4) It is not described how the experiment presented in Fig 3C, indicating that FN1 from macrophages can alter the stiffness of collagen, is performed.
5) Although the ECM stiffening effect of FN1 is described by the authors as the reason for the observed effects, several experiments indicate that the effects is unrelated to matrix stiffness (Fig S2). This should be addressed.

Reviewer #2

(Remarks to the Author)
This paper provides insights into the role of stiffness and ECM remodeling in TAMs and their contribution to tumor progression in NSCLC. The authors further introduce a mechanistic pathway in which FN1 participates in the anti-inflammatory response of TAMs and demonstrate how its absence promotes a metabolic shift toward glycolysis. According to the model presented, FN1 deficiency is associated with a proinflammatory phenotype and increased glycolytic activity in TAMs. By establishing a link between FN1 and the mTOR pathway, the study suggests that FN1 regulates this metabolic switch through mTOR signaling. The possibility that FN1 may influence the metabolic shift via post-translational modifications of metabolic enzymes is particularly intriguing and merits further investigation.
I believe there are several critical areas where substantial clarification and additional experimentation are required.

1. The authors propose that FN1 deficiency leads to increased hypoxia in TAMs, which they claim could enhance the efficacy of immune checkpoint blockade (ICB). However, previous studies (e.g., PMID: 27482883 and PMID: 33986123) have demonstrated that hypoxia in TAMs and within the TME typically promotes a pro-tumor phenotype and resistance to immunotherapy. It would therefore be important for the authors to address this apparent paradox and clarify how hypoxia, in their model, results in improved ICB efficacy.
2. In Figure 1, the authors present scRNA-seq data from NSCLC tumor tissues (n = 10 patients). However, there is no clear cluster annotated as a tumor cell subpopulation. If the epithelial cell cluster is being considered as the tumor cell subpopulation, its representation is relatively low (approximately 14% of all cells). The authors should provide an explanation for this low proportion of tumor cells and discuss whether this may be due to technical limitations of the experiment or biological variability.
3. In Figure 2, the macrophage clustering annotation appears inconsistent when compared with the clustering presented in Figure 1. In particular, the cluster annotated as lipid metabolism associated TAMs, which displays high FN1 expression and is highlighted as a target subpopulation in Figure 1, is not annotated with sufficient clarity or consistency. The authors must provide a more rigorous justification for the annotation criteria and ensure consistent annotarion across figures to substantiate their conclusions.
4. Since Figures 2 and 7 present T cell exhaustion as a key outcome of FN1 inhibition, the current evidence is not sufficient to fully support this claim. To demonstrate a direct mechanistic link, an additional validation experiment is required. Specifically, using RAG1-deficient mice in combination with FN1 inhibition would be essential to confirm whether the observed T cell exhaustion is directly dependent on FN1 deficiency in macrophages. Without this experiment, the interpretation of FN1-mediated effects on T cell exhaustion remains incomplete.
5. In Figure 6H, FN1^fl/fl + RAPA treatment demonstrates a stronger antitumor effect in the size of the tumor compared with FN1^ΔLyz2 + RAPA, which instead shows a partial rescue of tumor growth in the absence of FN1. The authors should clarify how this finding fits with their proposed model and provide an explanation for this unexpected effect. To properly interpret the effects of mTOR inhibition in this study, it is critical to first clarify which biological process—glycolysis or autophagy—is being specifically targeted. Since inhibition of mTOR can lead to off-target effects, relying solely on rapamycin is not sufficient. Previous studies have demonstrated incomplete inhibition of 4E-BP1 by rapamycin and pathway-specific differences in sensitivity, particularly in relation to glycolysis. Therefore, the use of a more comprehensive inhibitor such as Torin 1 or Torin 2, which effectively suppress both mTORC1 and mTORC2, is required to ensure robust pathway inhibition and allow for a clear and reliable interpretation of the results. Without such an approach, the conclusions regarding mTOR-dependent regulation remain insufficiently supported.
6. The authors show that PFKP undergoes proteasome-mediated degradation, as PFKP upregulation in FN1-inhibited MDMs was reduced by MG-132 treatment. However, to confirm that this effect is indeed mediated through FN1 inhibition and proteasome activity, a ubiquitination assay is required. Without direct evidence of PFKP ubiquitination, the mechanistic link between FN1 inhibition, MG-132 treatment, and PFKP stability is not fully confirmed.
7. Finally, in Figures 5J–N, quantification of the images would greatly improve the interpretation and provide a more comprehensive overview of the validation experiments.

Reviewer #3

(Remarks to the Author)
The work by Li and colleagues describes the unexpected function of fibronectin (FN1) in tumor-associated macrophages (TAMs). The results are surprising as FN1 is typically considered a component of the extracellular matrix (ECM), and is normally produced by fibroblasts. While the authors show that the latter cells are indeed a major source of FN1, TAMs also express FN1, albeit at lower levels. The authors moreover show that FN1 impacts on the tumor microenvironment (TME), affecting T-cell invasion and TAM polarization. Part of this mechanism is through FN1 regulating the actin cytoskeleton of TAMs, modulating RAC-mTOR, and subsequently autophagy and glycolysis. In general, using a broad range of techniques, the manuscript contributes many novel aspects of crosstalk between ECM components and the cellular TME to the field, although the total and final impact on in vivo tumor growth and survival stay surprisingly modest. However, the manuscript has merits, but certain elements of the work require more validation:

Major points:
-A central point of the manuscript is that extracellular FN1 can evoke an intracellular signaling cascade (Actin-RAC-mTOR-Autophagy-Glycolysis). However, it remains unproven that FN1 is indeed extracellular. The authors should provide more experimental evidence on the localization of FN1. One way to address this would be by investigating whether FN-receptors (Integrins) play a crucial role in the proposed signal transduction cascade. Another approach might be the addition of FN-neutralizing antibodies.
-Figure 4: Is the PFKP protein also more stable in FN1deltaLyz2 conditions (or FI) as measured in a cycloheximide assay?
-Do FN-depleted or-inhibited cells display an autophagy defect which can be seen as altered LC3B speckles in microscopy?
-The authors claim to have found a novel concept between stiffness and autophagy, but a similar concept has already been described before in CAFs (Hupfer et al., 118(40), 2021, PNAS). This would argue that different TME cell types use a comparable mechanism for adaptation to stiffness and this should be discussed by the authors as it might be of therapeutic relevance.

Minor points:
Line 334: …plasma…(i.e. cytoplasm)
Figure legends: Sometimes the authors should add more info, for example figure 1k: which sample is the heatmap referring to? Human? Mouse? Public data?

Figure legends: e.g. figure 5: When showing one representative experiment (e.g. WB), add info on how many n were performed in total.
Figure 4B: What is shown on the x-axis of the panel? Significance? What is the scale?

Version 1:

Reviewer comments:

Reviewer #1

(Remarks to the Author)
The authors address several of my critique points satisfactorily. However, some important information still needs to be explicitly stated in the manuscript.

Referring to an RGD peptide as a “fibronectin inhibitor” is misleading. RGD peptides function as integrin-binding competitors that block the interaction between multiple integrins and multiple extracellular matrix ligands. They do not specifically inhibit fibronectin. This mechanism of action should be clearly described in the manuscript, and the term “fibronectin inhibitor” should not be used.

In addition, the information provided regarding the experimental setup of Figure 3C remains insufficient. The description stating that “macrophages were cultured in Collagen I and seeded on AFM-compatible glass-bottom dishes” lacks critical methodological details. It is unclear how the cells were cultured in collagen. Were they embedded in a 3D collagen matrix or cultured on top of a collagen coating? What was the collagen concentration? Was the collagen neutralized and polymerized? These details are necessary to understand and interpret the experimental conditions.

Reviewer #2

(Remarks to the Author)
The authors have addressed my comments appropriately, and I am satisfied with the revisions made to the manuscript.

Reviewer #3

(Remarks to the Author)
The authors have adequately addressed all of my previously raised concerns.

Open Access This Peer Review File is licensed under a Creative Commons Attribution 4.0 International License, which permits use, sharing, adaptation, distribution and reproduction in any medium or format, as long as you give appropriate credit to the original author(s) and the source, provide a link to the Creative Commons license, and indicate if changes were made.
In cases where reviewers are anonymous, credit should be given to 'Anonymous Referee' and the source.

The images or other third party material in this Peer Review File are included in the article's Creative Commons license, unless indicated otherwise in a credit line to the material. If material is not included in the article's Creative Commons license and your intended use is not permitted by statutory regulation or exceeds the permitted use, you will need to obtain permission directly from the copyright holder.
To view a copy of this license, visit https://creativecommons.org/licenses/by/4.0/

REVIEWER COMMENTS

Reviewer #1:

In the manuscript "FN1+ macrophages prevent anti-tumor immunity via ECM stiffening and autophagy-mediated immunosuppression", Li et al. investigates how fibronectin (FN1)-producing tumor-associated macrophages (TAMs) promote immune suppression in non-small cell lung cancer (NSCLC). Using single-cell RNA sequencing, they find that FN1+ TAMs comprise a specific immunosuppressive macrophage subset. The authors suggest that FN1+ macrophages stiffen the ECM, creating an immunosuppressive ECM. Deletion of FN1 in TAMs using conditional knockout mice changes the cells towards a less immunosuppressive phenotype.

Mechanistically, the authors suggest that FN1 activates cytoskeletal assembly and autophagy through inhibition of the RAC1–mTOR pathway, suppressing macrophage glycolysis and promoting an anti-inflammatory phenotype. Finally, FN1 deletion in TAMs or treatment with a FN1 inhibitor leads to delayed tumor growth.

The paper contains a large amount of data and several of the findings are very interesting. Many observations are also very surprising warranting particularly thorough control experiments. Unfortunately, the paper lacks important information necessary for a thorough evaluation of the data, and additional essential experiments are required to support the claims of the paper.

Critique points:

1. *The drastic difference between the TAMs in FN1fl/fl mice and FN1Lyz2 mice and the differences in tumor growth, makes it essential to know the details of the experimental mice that were compared (background, age, gender, breeding). Also, an additional control group consisting of the Lyz2-Cre mice should be included.*

Response:

Thank you for your insightful feedback. We have thoroughly revised the Methods to address your concerns about experimental rigor and transparency (page 14). Below is a detailed response:

The $FN1^{fl/fl}$ and $FN1^{\Delta Lyz2}$ mice were purchased from Cyagen Biosciences (Suzhou, China). Macrophage-specific FN1 knockout mice ($FN1^{\Delta Lyz2}$, heterozygous for Lyz2-Cre) were generated by crossing $FN1^{fl/fl}$ mice with Lyz2-cre mice, and their F1 heterozygotes were crossed with $FN1^{fl/fl}$ mice. The mice were housed under controlled, specific pathogen-free conditions and received humane care according to animal welfare guidelines throughout the study.

To minimize environmental variables, we utilized **littermate controls** generated by crossing $FN1^{fl/fl}$ with $FN1^{\Delta Lyz2}$ mice (heterozygous for Lyz2-Cre), yielding litters with approximately 50% $FN1^{fl/fl}$ and 50% $FN1^{\Delta Lyz2}$ pups (Mendelian inheritance confirmed). All experiments employed age-matched (6-8 weeks old) and sex-matched littermates housed in the same facility to ensure consistency. Figures 2–6 were conducted using female mice only; Figure 7 (combination therapy with anti-PD-1) were conducted twice using male and female mice separately.

We acknowledge your suggestion to include Lyz2-Cre controls. However, our breeding strategy and housing conditions render this approach less informative: Lyz2-Cre mice raised in separate cages would introduce environmental variability, potentially confounding comparisons. We performed tissue-specific validation to confirm Cre activity: PCR and RT-qPCR analysis of toe, tail, liver, spleen, and bone marrow tissues demonstrated Cre expression predominantly in macrophage-rich tissues (liver, spleen and bone marrow), with minimal activity in non-hematopoietic tissues (Figure a, see as below). Besides, scRNA-seq result (Figure 2B) confirmed target-specific FN1 ablation: FN1 expression was significantly reduced in macrophages from FN1$^{\Delta Lyz2}$ mice but remained unchanged in fibroblasts, confirming that observed tumor growth differences stem from FN1 deletion in macrophages rather than Cre toxicity. This approach aligns with recent studies (e.g., PMID:38744281, PMID:38838160) that prioritize littermate controls over isolated Cre-driver lines to mitigate environmental confounding.

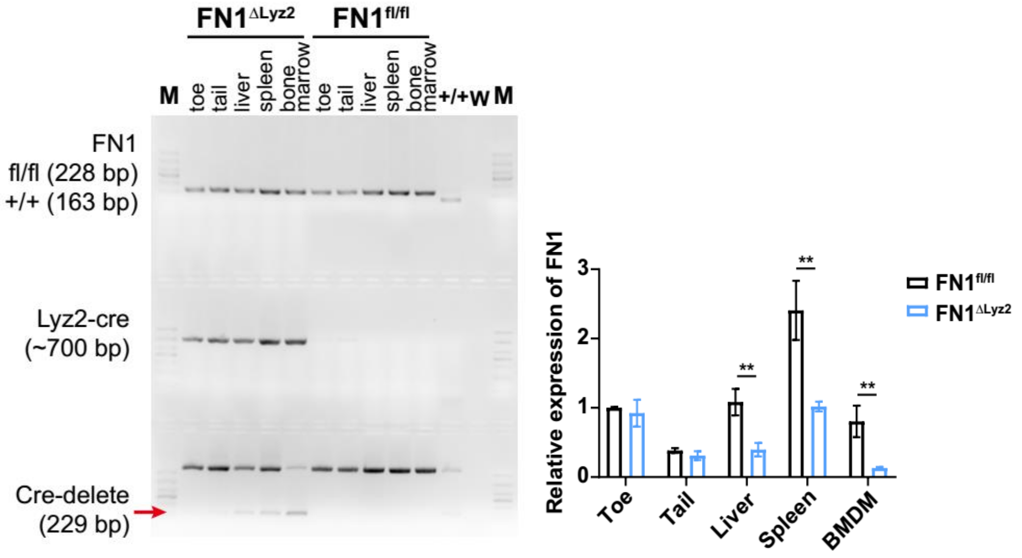

Figure a. Genotyping and qRT-PCR analysis of FN1 were performed in toe, tail, liver, spleen, and bone marrow tissues from homozygous knock-in (fl/fl) mice and myeloid-specific FN1-depleted (ΔLyz2) mice. $n = 3$ biologically independent samples. Data are displayed as the mean ± SD. **p < 0.01, two-tailed unpaired *t*-test.

2. *Additional information on the fibronectin inhibitor should be provided, including data that supports its use for in vivo experiments. Control experiments (in vivo and in vitro) demonstrating a fibronectin "inhibiting" function should be included.*

Response:

Thank you for your valuable feedback. The fibronectin inhibitor (Santa Cruz Biotechnology, CAS 91037-65-9, https://www.scbt.com/p/fibronectin-inhibitor-91037-65-9) is a synthetic tetrapeptide (sequence: H-Arg-Gly-Asp-Ser-OH) that blocks fibronectin binding to integrin receptors by targeting the RGD recognition motif (PMID: 3876125). This mechanism has been well-documented in previous studies to disrupt fibronectin-integrin interactions, thereby inhibiting integrin receptor function and associated cellular processes. Prior studies have also validated its efficacy *in vivo* (PMID: 19272161, 32377396). We have now included these references explicitly in the

revised manuscript to substantiate its suitability for *in vivo* and *in vitro* experiments (page 5 and page 9).

3. *Critical information regarding the flow cytometry experiments is lacking, including antibody information and information on whether individual markers (such as FN1) were detected intracellularly or on the cell surface.*

Response:

Thank you for highlighting the need for detailed flow cytometry methodology. All antibodies information including catalog numbers, suppliers are provided in the Nature Portfolio Reporting Summary.

FN1 is an intracellular secreted protein, necessitating intracellular staining protocols. Following the fixation/permeabilization steps, intracellular FN1 staining was performed using the validated primary antibody (Anti-Fibronectin antibody, Abcam, Cat# ab32419) and fluorescent secondary antibody (Alexa Fluor® 488, Abcam, Cat# ab150077), and Rabbit monoclonal IgG (Abcam, Cat# ab172730) was used as the isotype control. We have thoroughly revised the Methods section to address these critical points (page 19).

4. *It is not described how the experiment presented in Fig 3C, indicating that FN1 from macrophages can alter the stiffness of collagen, is performed.*

Response:

Thank you for your request for clarification regarding the experimental setup for Figure 3C. For ECM measurements, the macrophages (2×10^6 cells/mL) were cultured in Collagen I (Corning, CLS354249) and seeded on AFM-compatible glass-bottom dishes. After 48 h of incubation, cells were fixed with paraformaldehyde. The following day, the samples were quantified using AFM. The cantilevers of the AFM probes (NP-O, Bruker) were glued to a polystyrene bead (diameter: 8 μm, Beijing Dk Nano Technology Co., Ltd.) using an epoxy adhesive. The cantilevers were tapped onto the tumor sections or the flat top surface of the collagen hydrogel, and three 10 μm × 10 μm stiffness maps (20 × 20 raster series) were constructed for each sample. The indentations were performed at a loading force of 2 nN and a constant speed of 2 μm/s (NanoWizard IV, JPK). Young's modulus was determined by fitting a force–distance curve to the Hertz–Sneddon model. We have revised the Methods section to address these critical points (page 17).

5. *Although the ECM stiffening effect of FN1 is described by the authors as the reason for the observed effects, several experiments indicate that the effects is unrelated to matrix stiffness (Fig S2). This should be addressed.*

Response:

Thank you for your comment. We emphasize that FN1 functions through dual mechanistic pathways–both physical and biomechanical. For physical mechanism, macrophage-derived FN1 enhances ECM stiffness, forming a stiffened matrix that as a physical barrier, limiting T cell infiltration into tumor stroma. Simultaneously, FN1 serves as a biomechanical signal by binding to macrophage integrin receptors,

triggering F-actin cytoskeletal remodeling, directly inducing an immunosuppressive function.

The experiments in Figure S2 do not contradict the ECM stiffening hypothesis but rather complement it by revealing FN1's multifaceted effects: while ECM stiffening contributes to physical immune exclusion, FN1's integrin-driven signaling provides an additional biochemical layer of immune modulation. These pathways act synergistically to regulate tumor microenvironment.

Reviewer #2:

This paper provides insights into the role of stiffness and ECM remodeling in TAMs and their contribution to tumor progression in NSCLC. The authors further introduce a mechanistic pathway in which FN1 participates in the anti-inflammatory response of TAMs and demonstrate how its absence promotes a metabolic shift toward glycolysis. According to the model presented, FN1 deficiency is associated with a proinflammatory phenotype and increased glycolytic activity in TAMs. By establishing a link between FN1 and the mTOR pathway, the study suggests that FN1 regulates this metabolic switch through mTOR signaling. The possibility that FN1 may influence the metabolic shift via post-translational modifications of metabolic enzymes is particularly intriguing and merits further investigation.

I believe there are several critical areas where substantial clarification and additional experimentation are required.

1. *The authors propose that FN1 deficiency leads to increased hypoxia in TAMs, which they claim could enhance the efficacy of immune checkpoint blockade (ICB). However, previous studies (e.g., PMID: 27482883 and PMID: 33986123) have demonstrated that hypoxia in TAMs and within the TME typically promotes a pro-tumor phenotype and resistance to immunotherapy. It would therefore be important for the authors to address this apparent paradox and clarify how hypoxia, in their model, results in improved ICB efficacy.*

Response:

Thank you for your comment. Although hypoxia pathways were enriched in FN1-deficient TAMs in this study, FN1 deficiency upregulated glycolysis in macrophages, leading to the production of pro-inflammatory cytokines and reversal of the immunosuppressive microenvironment, which are the key points we are focusing on. Moreover, multiple previous studies have also reported that enhanced glycolysis in macrophages can improve anti-tumor efficacy. For example: Chimeric antigen receptor macrophages targeting prostate-specific membrane antigen undergo glycolytic reprogramming (enhanced glycolysis and suppressed oxidative phosphorylation) upon antigen recognition, supporting M1 polarization and enhancing phagocytosis and cytotoxicity (PMID: 41163070). Targeting mannosylated mitochondria transplantation resets the metabolic mode of TAMs from oxidative phosphorylation to glycolysis, promoting their M1 polarization, forming an anti-tumor pro-inflammatory TME, and significantly enhancing the efficacy of anti-PD-L1 therapy (PMID: 39119940).

Additionally, in this study FN1 deficiency in TAMs reduces tumor tissue stiffness

and promotes T-cell infiltration, thereby overall enhancing ICB efficacy. CyTOF results in Figure 7 showed that T cells in tumors from FN1$^{\Delta Lyz2}$ mice treated with anti-PD-1 exhibited the strongest cytotoxic function.

While FN1 deficiency in macrophages enhanced ICB efficacy, the increased glycolysis does lead to a hypoxic environment, which may also explain why tumor progression was not fully reversed. Based on this consideration, we have revised the description in the Limitations of the study accordingly (page 13).

2. *In Figure 1, the authors present scRNA-seq data from NSCLC tumor tissues (n = 10 patients). However, there is no clear cluster annotated as a tumor cell subpopulation. If the epithelial cell cluster is being considered as the tumor cell subpopulation, its representation is relatively low (approximately 14% of all cells). The authors should provide an explanation for this low proportion of tumor cells and discuss whether this may be due to technical limitations of the experiment or biological variability.*

Response:

Thank you for your comment. In single-cell RNA sequencing (scRNA-seq) studies of NSCLC, the proportion of tumor cells or epithelial cells in lung cancer tissues can vary significantly depending on sample origin (biopsy or resection), pathological type and stage, patient-specific biological variability (e.g., immune cell infiltration levels), and whether cell sorting was performed. Theoretically, epithelial cells (including tumor cells) constitute one of the major cellular populations; however, their actual representation in sequencing data can range widely.

Meanwhile, we have examined 17 NSCLC scRNA-seq datasets from the TISCH2 platform, of which 11 included epithelial cells alongside various immune cell populations. Statistical analysis of epithelial/tumor cell proportions across these datasets revealed a range of 2%-93% (Table 1). In our scRNA-seq data, the epithelial cell proportion is approximately 15%, which is comparable to the proportions observed in multiple datasets from the TISCH2 platform. Notably, 70% of the tissue samples in our study were derived from stage I patients, which may partially explain the relatively lower epithelial cell proportion observed in Figure 1.

Table 1. Epithelial cell proportion in 11 NSCLC single-cell datasets from the TISCH2 platform.

Dataset Name	Patients	Total Cells	Cell Type	Count	Proportion (%)	Platform	Pri/Meta	PMID
NSCLC_EMTAB6149	5	40,218	Malignant	5,728	14%	10x Genomics	Primary	29988129
NSCLC_GSE117570	4	11,453	Epithelial	501	4%	10x Genomics	Primary	31033233
			Malignant	2,721	24%			
NSCLC_GSE127465	7	31,179	Malignant	3,995	13%	Smart-seq2	Primary	30979687
NSCLC_GSE131907	44	203,298	Epithelial	36,145	18%	10x Genomics	Primary, Metastatic	32385277
NSCLC_GSE143423	3	12,193	Malignant	9,237	76%	10x Genomics	Metastatic	None
NSCLC_GSE146100	1	10,996	Epithelial	975	9%	10x Genomics	Primary	33820821
NSCLC_GSE148071	42	82,267	Alveolar	4,605	6%	GEXSCOPETM	Primary	33953163

			Epithelial	1,153	1%			
			Malignant	48,118	58%			
NSCLC_GSE149655	2	4,433	Alveolar	3,578	81%	10x Genomics	Primary	32891189
			Ciliated	330	7%			
			Club	211	5%			
NSCLC_GSE150660	2	5,407	Malignant	322	6%	10x Genomics	Metastatic	32675368
NSCLC_GSE153935	12	3,658	Alveolar	315	9%	10x Genomics	Primary	None
			Epithelial	192	5%			
NSCLC_GSE162498	11	68,255	Epithelial	1,255	2%	10x Genomics	Primary	33514641

3. *In Figure 2, the macrophage clustering annotation appears inconsistent when compared with the clustering presented in Figure 1. In particular, the cluster annotated as lipid metabolism associated TAMs, which displays high FN1 expression and is highlighted as a target subpopulation in Figure 1, is not annotated with sufficient clarity or consistency. The authors must provide a more rigorous justification for the annotation criteria and ensure consistent annotarion across figures to substantiate their conclusions.*

Response:

Thank you for your critical feedback. The macrophage clusters were annotated based on GSVA enrichment of pathways. This annotation reflects the functional state of these macrophages. In Figure 1 (human NSCLC tumor scRNA-seq), lipid metabolism-associated TAMs were enriched in cholesterol homeostasis, fatty acid metabolism, adipogenesis, and oxidative phosphorylation, with high expression of lipid metabolism-related genes (*FABP3, FABP4, MARCO*). For Figure 2 (mouse LLC subcutaneous tumor scRNA-seq), while the gene/pathway enrichment patterns differ from human data, we maintained consistent annotation logic. Common annotations across both figures included innate immune monocytes, hypoxia-TAM, and IFN-TAM subsets. The fatty acid metabolism-enriched cluster in mice was identified lysosome-TAM through prominent lysosomal pathway enrichment, aligning with state-based annotation principles (Figure 2D, 2E). To maintain consistency with Figure 1, we have revised the designation of "lysosome-TAM" in Figure 2 to "Lyso/Fatty acid-TAM".

Importantly, $FN1^{\Delta Lyz2}$ mice showed reduced proportion of Lyso/Fatty acid-TAM (Figure 2F), suggesting a positive correlation between FN1 and macrophage lipid metabolism/lysosomal function. Multiple studies have reported that macrophages with enriched lipid metabolism demonstrate enhanced immunosuppressive capabilities (PMID: 39357545, 37604643). This provides mechanistic insight into how FN1-mediated immunosuppressive functions in TAMs through the lysosomal pathway, which aligns with subsequent investigations exploring FN1's role in autophagy-mediated immunosuppressive pathways in TAMs. We have carefully revised the main text with yellow highlight (page 5).

4. *Since Figures 2 and 7 present T cell exhaustion as a key outcome of FN1 inhibition, the current evidence is not sufficient to fully support this claim. To demonstrate a direct mechanistic link, an additional validation experiment is required. Specifically, using*

RAG1-deficient mice in combination with FN1 inhibition would be essential to confirm whether the observed T cell exhaustion is directly dependent on FN1 deficiency in macrophages. Without this experiment, the interpretation of FN1-mediated effects on T cell exhaustion remains incomplete.

Response:

Thank you for your comment. We note that the question raised appears inconsistent with the core content and findings presented in our manuscript. According to editorial communication records, the editor made two separate attempts to contact you for clarification and verification of this specific issue. Unfortunately, we did not receive more response. Consequently, based on our comprehensive understanding of the study's context and experimental design, we addressed this concern through supplementary experiments and detailed explanations in the following text.

We note that your concern about Figures 2 and 7 framing T cell exhaustion as an outcome of FN1 inhibition does not align with our experimental findings. Our data consistently indicate that TAMs-derived FN1 induced an immunosuppressive microenvironment, while FN1 deficiency in macrophages enhanced T cell cytotoxic function. Specifically, scRNA-seq analysis revealed that in $FN1^{\Delta Lyz2}$ mice tumors, $CD4^+$ T cells exhibited significantly elevated expression of *TNF* and IFN-inducible genes (*Ifitm2* and *Ifit1*), while $CD8^+$ T cells and NK cells showed markedly higher levels of IFN-γ (*Ifng*), perforin (*Prf1*), and granzyme (*Gzm*) genes compared to littermate controls (Figure 2J). To validate these observations functionally, we co-cultured mouse $CD8^+$ T cells with BMDMs and found that $CD8^+$ T cells from the $FN1^{\Delta Lyz2}$ group produced substantially higher levels of IFN-γ and perforin (Figure S2O).

To further dissect the functional role of FN1, we blocked FN1-integrin interaction using the inhibitor ATN-161 in BMDM-$CD8^+$ T cell co-cultures. The results demonstrated that $CD8^+$ T cells co-cultured with ATN-161-pretreated BMDMs exhibited enhanced granzyme B and IFN-γ production (Figure b, see as below). These findings collectively support the conclusion that macrophage-derived FN1 promotes immunosuppressive functions, thereby suppressing T cell cytotoxicity and reinforcing the immunosuppressive tumor niche.

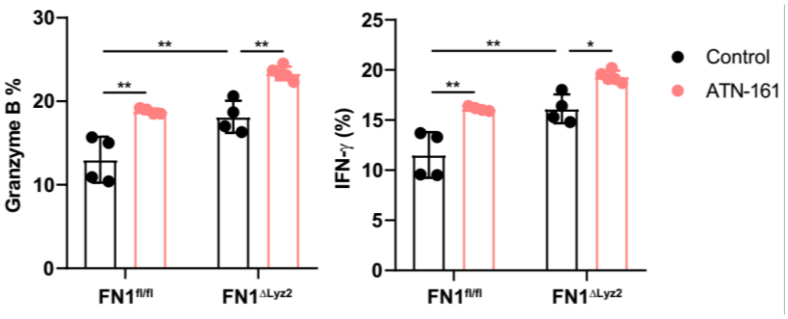

Figure b. Flow cytometry analysis of perforin and IFN-γ expression in $CD8^+$ T cells co-cultured with ATN-161 (10 μM) treated $FN1^{fl/fl}$ and $FN1^{\Delta Lyz2}$ BMDMs in a ratio of 1:2 for 24h. *n* = 4 biologically independent samples. Data are displayed as the mean ± SD. *$p < 0.05$, **$p < 0.01$, two-way ANOVA.

We appreciate the suggestion to include experiments with RAG1-deficient mice;

however, our current evidence—through multiple orthogonal approaches including genetic knockout, pharmacological inhibition, and functional assays—already provides robust mechanistic insights into how macrophage-specific FN1 deficiency modulates T cell function. The observed enhancement of cytotoxic markers and effector molecule production in FN1-deficient macrophage group or following FN1-integrin blockade directly contradicts the premise of T cell exhaustion as an outcome, instead indicating a reversal of immunosuppression and enhancement of anti-tumor immunity.

5. *In Figure 6H, FN1^fl/fl + RAPA treatment demonstrates a stronger antitumor effect in the size of the tumor compared with FN1^ΔLyz2 + RAPA, which instead shows a partial rescue of tumor growth in the absence of FN1. The authors should clarify how this finding fits with their proposed model and provide an explanation for this unexpected effect. To properly interpret the effects of mTOR inhibition in this study, it is critical to first clarify which biological process—glycolysis or autophagy—is being specifically targeted. Since inhibition of mTOR can lead to off-target effects, relying solely on rapamycin is not sufficient. Previous studies have demonstrated incomplete inhibition of 4E-BP1 by rapamycin and pathway-specific differences in sensitivity, particularly in relation to glycolysis. Therefore, the use of a more comprehensive inhibitor such as Torin 1 or Torin 2, which effectively suppress both mTORC1 and mTORC2, is required to ensure robust pathway inhibition and allow for a clear and reliable interpretation of the results. Without such an approach, the conclusions regarding mTOR-dependent regulation remain insufficiently supported.*

Response:

Thank you for your comments. For the first comment about tumor volume, our data showed that rapamycin (RAPA) alone suppressed tumor growth, this effect arises because rapamycin, as an mTOR inhibitor, can interfere with mRNA transcription, protein synthesis and cell cycle transition, inducing autophagy and apoptosis in tumor cells, and inhibiting pro-oncogenic pathways (e.g., EGFR/MET), thereby exerting anti-tumor effects (PMID: 27635236, 39762538). However, RAPA combined with FN1 blockade did not further impair tumor progression compared with the monotherapy groups (Figure 6B, 6C and 6H). This suggests that inhibiting FN1 may have an opposite effect on activating autophagy, which need further exploration. The discrepancy in tumor size between $FN1^{fl/fl}$ + RAPA and $FN1^{\Delta Lyz2}$ + RAPA groups may stem from the myelosuppressive side effects of rapamycin reducing macrophage populations within tumors. While the $FN1^{\Delta Lyz2}$ + RAPA group retained a higher macrophage count, rapamycin-induced autophagy activation in these macrophages triggered immunosuppressive functions, paradoxically promoting tumor growth.

The purpose we employed rapamycin *in vivo* is to verify that FN1 activated autophagy in TAMs, reduced glycolysis, and induced an immunosuppressive microenvironment. In our model, loss of FN1 in macrophages promoted pro-inflammatory cytokine production and enhanced T cell cytotoxicity, but RAPA co-treatment counteracts these effects. In addition, RAPA treatment alone significantly suppressed T cell functionality (Figure 6D, 6E, 6I and 6J). This might be because rapamycin itself can inhibit the activation and proliferation of $CD8^+$ T cells and reduce

the differentiation of T cells into effector phenotypes, thus can be used for immunosuppression after organ transplantation (PMID: 26122844, 33512760). This highlights a “double-edged sword”: while rapamycin kills tumor cells, it simultaneously impairs anti-tumor immunity, underscoring the need for cell-specific targeting. We have revised the Discussion to refine the aforementioned content and highlighted the relevant sections in yellow (page 12 and 13).

For the second concern about mTOR inhibition, we initially used rapamycin to probe the role of mTORC1-mediated autophagy in macrophage metabolic reprogramming, given its well-established role in autophagy induction. mTORC1 is a key regulator of autophagy, regulating different steps in the autophagy process, while mTORC2 also regulates glucose metabolism. Considering the reviewer’s suggestion, we performed additional experiments using Torin 1, a more comprehensive mTOR inhibitor that suppresses both mTORC1 and mTORC2 to validate the pathway. Consistent with our rapamycin results in Figure 5F, Torin 1 treatment reduced PKFP and p62 levels, enhanced LC3-II/I ratios, and decreased phosphorylation of RPS6/S6 and 4E-BP1 (Figure c, see as below). These findings confirm that FN1-driven autophagy activation in macrophages suppresses glycolysis, thereby promoting immunosuppression.

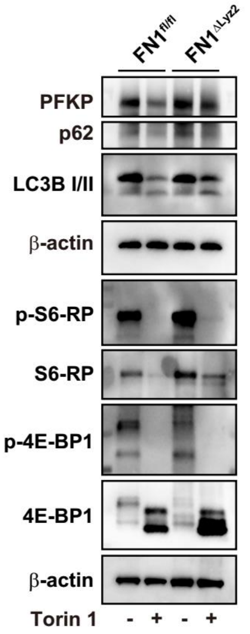

Figure c. Representative western blotting image of PFKP, p62, p-S6-RP, S6-RP, p-4E-BP1, 4E-BP1, LC3BI/II, and β-actin in BMDMs from $FN1^{fl/fl}$ and $FN1^{\Delta Lyz2}$ mice treated with Torin 1 for 12h (100 nM). Experiment was repeated three times independently with similar results.

6. *The authors show that PFKP undergoes proteasome-mediated degradation, as PFKP upregulation in FN1-inhibited MDMs was reduced by MG-132 treatment. However, to confirm that this effect is indeed mediated through FN1 inhibition and proteasome activity, a ubiquitination assay is required. Without direct evidence of PFKP ubiquitination, the mechanistic link between FN1 inhibition, MG-132 treatment, and PFKP stability is not fully confirmed.*

Response:

Thank you for your valuable feedback. Our results demonstrated that FN1 deficiency reduced PFKP degradation through autophagy inhibition rather than proteasome-mediated pathways. PFKP upregulation in FN1-inhibited MDMs was reduced by MG-132 treatment (Figure 5A), contradicting expectations for proteasomal degradation. Nevertheless, FN1 loss significantly enhanced PFKP, p62, and p-mTOR levels, with CQ (autophagy inhibitor) further amplifying these effects, while RAPA (autophagy activator) markedly impaired them even in FN1-deficient cells (Figures 5B–5F). This supported that FN1 deficiency inhibited autophagy-dependent PFKP degradation.

We have addressed your concern by performing ubiquitination assays to directly examine the mechanistic link between FN1 inhibition, MG-132 treatment, and PFKP stability. Ubiquitination assays confirmed that FN1$^{\Delta Lyz2}$ BMDMs exhibit higher PFKP ubiquitination compared to FN1$^{fl/fl}$ controls (Figure d, see as below), providing direct evidence that PFKP accumulation is not mediated by proteasomal pathways. These findings conclusively established that FN1 inhibition stabilized PFKP by suppressing autophagy rather than proteasomal degradation.

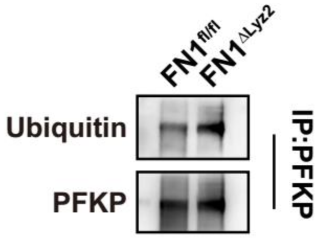

Figure d. Immunoprecipitation image of PFKP and ubiquitin in BMDMs from FN1$^{fl/fl}$ and FN1$^{\Delta Lyz2}$ mice. Experiment was repeated three times independently with similar results.

7. *Finally, in Figures 5J–N, quantification of the images would greatly improve the interpretation and provide a more comprehensive overview of the validation experiments.*

Response:

Thank you for your suggestion. The quantification of images in Figures 5J–N is provided in Figure S6A–S6C, S6E and S6F.

Reviewer #3:

The work by Li and colleagues describes the unexpected function of fibronectin (FN1) in tumor-associated macrophages (TAMs). The results are surprising as FN1 is typically considered a component of the extracellular matrix (ECM), and is normally produced by fibroblasts. While the authors show that the latter cells are indeed a major source of FN1, TAMs also express FN1, albeit at lower levels. The authors moreover show that FN1 impacts on the tumor microenvironment (TME), affecting T-cell invasion and TAM polarization. Part of this mechanism is through FN1 regulating the actin cytoskeleton of TAMs, modulating RAC-mTOR, and subsequently autophagy and

glycolysis. In general, using a broad range of techniques, the manuscript contributes many novel aspects of crosstalk between ECM components and the cellular TME to the field, although the total and final impact on in vivo tumor growth and survival stay surprisingly modest. However, the manuscript has merits, but certain elements of the work require more validation:

Major points:

1. *A central point of the manuscript is that extracellular FN1 can evoke an intracellular signaling cascade (Actin-RAC-mTOR-Autophagy-Glycolysis). However, it remains unproven that FN1 is indeed extracellular. The authors should provide more experimental evidence on the localization of FN1. One way to address this would be by investigating whether FN-receptors (Integrins) play a crucial role in the proposed signal transduction cascade. Another approach might be the addition of FN-neutralizing antibodies.*

Response:

Thank you for your critical feedback. We have strengthened our evidence by incorporating additional experiments targeting integrin-mediated signaling. To further validate the extracellular FN1 triggered signals through its receptor integrin, we employed ATN-161, an integrin α5β1 and αvβ3 antagonist that blocks FN1-integrin binding (PMID: 16985061).

Western blot analysis shows that ATN-161 treatment significantly increased PFKP, p62, phosphorylation of RPS6/S6 and 4E-BP1, and decreased LC3II/I (Figure e, Figure S5L, see as below), indicating that integrin blockade recapitulated the metabolic and autophagy phenotypes induced by FN1 knockout. Confocal microscopy revealed that ATN-161 treatment decreased RAC1 co-localization with F-actin while increasing its co-localization with mTOR (Figure f, see as below), which was added in this text (Figure S6D). This supports the model where extracellular FN1-integrin interaction drives the F-actin-RAC1-mTOR signaling axis.

These findings confirmed that extracellular FN1 initiated an intracellular signaling cascade (F-actin-RAC1-mTOR-autophagy) that modulated PFKP degradation. The integrin blockade experiments, combined with Fibronectin Inhibitor (also a blocker of the binding site between FN1 and integrin) data, provide direct experimental evidences that FN1 acts extracellularly to transduce signals through integrin receptors, thereby establishing the proposed pathway with robust mechanistic validation. We have revised the main text to refine the aforementioned content and highlighted the relevant sections in yellow (page 8 and 9).

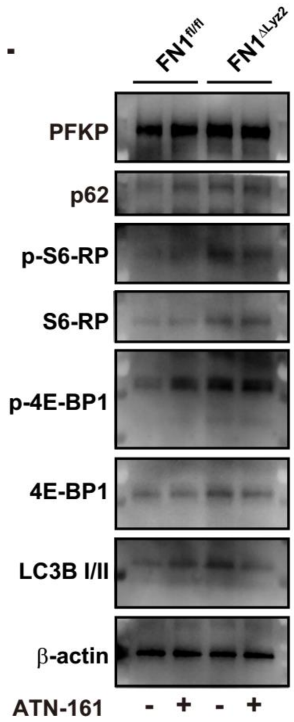

Figure e. Representative western blotting image of PFKP, p62, p-S6-RP, S6-RP, p-4E-BP1, 4E-BP1, LC3BI/II, and β-actin expression in BMDMs from FN1$^{fl/fl}$ and FN1$^{\Delta Lyz2}$ mice treated with ATN-161 (10 μM). Experiment was repeated three times independently with similar results.

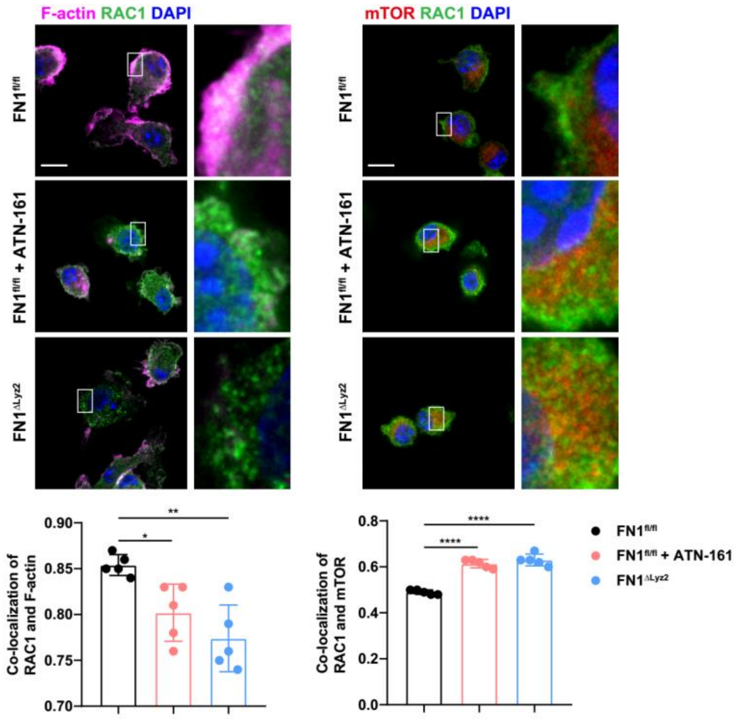

Figure f. Representative immunofluorescence image and quantification of co-localizaition of F-actin and RAC1, mTOR and RAC1 in BMDMs from FN1$^{fl/fl}$ and FN1$^{\Delta Lyz2}$ mice treated with ATN-161 (10 μM). Scale bar, 10 μm. *n* = 5 biologically independent samples. Data are displayed as the mean ± SD. *p < 0.05, **p < 0.01,

****p < 0.0001, one-way ANOVA.

2. *Figure 4: Is the PFKP protein also more stable in FN1deltaLyz2 conditions (or FI) as measured in a cycloheximide assay?*

Response:

Thank you for your suggestion. We performed cycloheximide chase assays to directly assess PFKP stability in $FN1^{\Delta Lyz2}$ BMDMs. Specifically, BMDMs were treated with cycloheximide (50 μg/mL) and harvested at 0, 4, 8, 12, and 20 hours for protein analysis. Western blot results demonstrated that PFKP degradation was significantly slower in $FN1^{\Delta Lyz2}$ BMDMs compared to $FN1^{fl/fl}$ controls during the early time points (0–12 hours), while cell viability markedly decreased by 20 hours (Figure g, see as below). This finding aligns with our observation that $FN1^{\Delta Lyz2}$ BMDMs exhibit reduced autophagy activity relative to $FN1^{fl/fl}$ BMDMs. The cycloheximide assay results provide direct evidence that FN1 deficiency stabilized PFKP by inhibiting its degradation, which is consistent with our model linking FN1 to autophagy-dependent protein turnover.

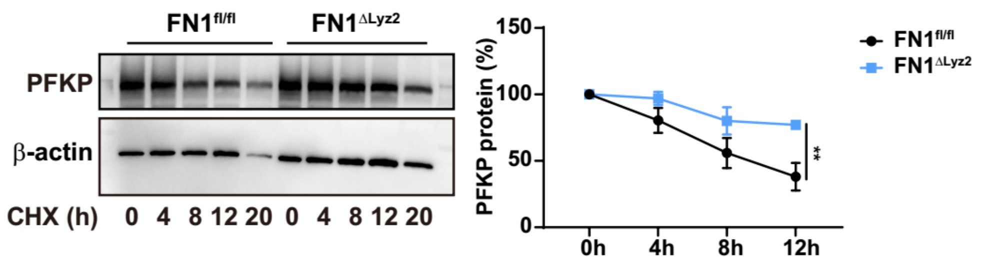

Figure g. Representative western blotting image and relative analysis of PFKP and β-actin expression in BMDMs from $FN1^{fl/fl}$ and $FN1^{\Delta Lyz2}$ mice treated with cycloheximide (CHX, 50 μg/mL) for 0, 4, 8, and 12 h. *n* = 4 biologically independent samples. Data are displayed as the mean ± SEM. **p < 0.01, two-way ANOVA.

3. *Do FN-depleted or-inhibited cells display an autophagy defect which can be seen as altered LC3B speckles in microscopy?*

Response:

Thank you for your valuable comment. We have performed quantitative analysis of LC3B puncta number and size in microscopy. Our results showed that FN1-deficient BMDMs exhibited an increased number and larger size of LC3B puncta (Figure h, see as below), indicating impaired autophagy. This microscopy data strengthens our conclusion that FN1 deficiency disrupts autophagy-dependent protein degradation pathways. These findings have been incorporated into the revised Figure S5H and the main text to provide direct visual evidence supporting the autophagy defect in FN1-depleted cells (page 8).

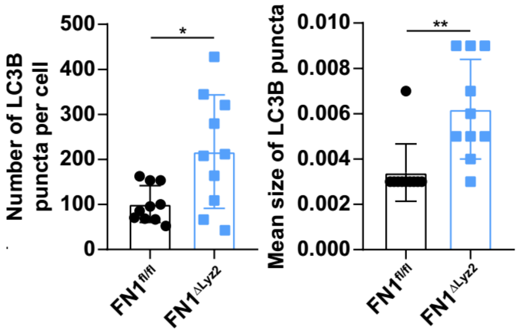

Figure h. Quantification of the number and mean size of LC3B puncta in $FN1^{fl/fl}$ and $FN1^{\Delta Lyz2}$ BMDMs within the confocal image in Figure S5H. *n* = 3 biologically independent samples. Each dot represents one field.

4. *The authors claim to have found a novel concept between stiffness and autophagy, but a similar concept has already been described before in CAFs (Hupfer et al., 118(40), 2021, PNAS). This would argue that different TME cell types use a comparable mechanism for adaptation to stiffness and this should be discussed by the authors as it might be of therapeutic relevance.*

Response:

Thank you for highlighting the important prior work on stiffness-induced autophagy in CAFs (Hupfer et al., PNAS 2021). We fully acknowledge that similar mechanisms have been described in other cell types of the TME. Specifically, Hupfer et al. demonstrated that CAFs sense matrix stiffness through the integrin αV-focal adhesion kinase module, leading to AMPKα stabilization at focal adhesions and subsequent autophagy induction to support cancer cell growth. In our study, CyTOF analysis revealed elevated glycolytic enzymes and inflammatory cytokines in macrophages, neutrophils, and DCs from $FN1^{\Delta Lyz2}$ mice, suggesting that stromal cells may share conserved stiffness-autophagy adaptation mechanisms. These provide a more comprehensive framework for understanding how matrix stiffness regulates autophagy and metabolism across distinct cellular populations, further highlighting the therapeutic potential of targeting shared stiffness-sensing pathways across TME cell types, offering new avenues for intervention in mechanobiology-driven disease processes. We have revised the related description in Discussion part with yellow highlight (page 12).

Minor points:

5. *Line 334: ...plasma...(i.e. cytoplasm)*

Response:

Thank you for your careful review and clarification. We have corrected the term as suggested to ensure precise terminology usage (page 9). Your feedback has been instrumental in improving the accuracy of our manuscript.

6. *Figure legends: Sometimes the authors should add more info, for example figure 1k: which sample is the heatmap referring to? Human? Mouse? Public data?*

Response:

Thank you for your valuable feedback on improving figure legends. We have carefully revised the figure legends to incorporate essential details about data sources (e.g., public datasets, specific cell types) to enhance clarity.

7. *Figure legends: e.g. figure 5: When showing one representative experiment (e.g. WB), add info on how many n were performed in total.*

Response:

Thank you for your insightful feedback. We have thoroughly revised all figure legends to explicitly include the number of data points and independent experiments performed for each representative experiment.

8. *Figure 4B: What is shown on the x-axis of the panel? Significance? What is the scale?*

Response:

Thank you for your careful review. We have added the x-axis in Figure 4B.

Thank you again for your kind help. Hope our revised manuscript is now publishable. Should further clarifications or revisions be required, we welcome the opportunity to refine our work. Do not hesitate to contact me if you have any more questions.

Sincerely,

Li Yang
Biotherapy Center and Cancer Center, The First Affiliated Hospital of Zhengzhou University
Zhengzhou 450052, Henan, China
Email: fccyangl1@zzu.edu.cn

Yi Zhang
Biotherapy Center and Cancer Center, The First Affiliated Hospital of Zhengzhou University
Zhengzhou 450052, Henan, China
Email: yizhang@zzu.edu.cn

Point-by-Point Response to Reviewer 1

REVIEWER COMMENTS

The authors address several of my critique points satisfactorily. However, some important information still needs to be explicitly stated in the manuscript.

1. *Referring to an RGD peptide as a "fibronectin inhibitor" is misleading. RGD peptides function as integrin-binding competitors that block the interaction between multiple integrins and multiple extracellular matrix ligands. They do not specifically inhibit fibronectin. This mechanism of action should be clearly described in the manuscript, and the term "fibronectin inhibitor" should not be used.*

Response:

Thank you for your insightful feedback. We have replaced all instances of "fibronectin inhibitor" with "fibronectin blocker" throughout the text, and added explicit explanation that RGD peptide competitively inhibit integrin-ECM interactions, including but not limited to fibronectin-integrin binding (page 5).

2. *In addition, the information provided regarding the experimental setup of Figure 3C remains insufficient. The description stating that "macrophages were cultured in Collagen I and seeded on AFM-compatible glass-bottom dishes" lacks critical methodological details. It is unclear how the cells were cultured in collagen. Were they embedded in a 3D collagen matrix or cultured on top of a collagen coating? What was the collagen concentration? Was the collagen neutralized and polymerized? These details are necessary to understand and interpret the experimental conditions.*

Response:

Thank you for your insightful feedback. Cells were embedded in a 3D collagen matrix. Macrophages were resuspended at 2×10^6 cells/mL in a mixture prepared on ice with a volume ratio of 1:1:8 of 3.7% Sodium Bicarbonate Solution (pH 9.5), 1M HEPES (Sigma, H0887), and Collagen I solution (Corning, CLS354249, concentration range 8–11 mg/mL). The pH of the mixture was confirmed to be between 7.0–7.4 using pH indicator paper. The cell suspension was supplemented with IL-4 (10 ng/mL) and IL-13 (10 ng/mL). A 10 µL aliquot of this mixture was seeded onto AFM-compatible glass-bottom dishes. The dishes were inverted and incubated at room temperature to allow solidification, followed by the addition of culture medium. We have revised the Methods to address your concerns (page 18).

Thank you again for your kind help. Hope our revised manuscript is now publishable. Should further clarifications or revisions be required, we welcome the opportunity to refine our work. Do not hesitate to contact me if you have any more questions.

Sincerely,

Li Yang
Biotherapy Center and Cancer Center, The First Affiliated Hospital of Zhengzhou University
Zhengzhou 450052, Henan, China
Email: fccyangl1@zzu.edu.cn

Yi Zhang
Biotherapy Center and Cancer Center, The First Affiliated Hospital of Zhengzhou University
Zhengzhou 450052, Henan, China
Email: yizhang@zzu.edu.cn
